# Systematic genetic analyses of GWAS data reveal an association between the immune system and insomnia

**DOI:** 10.1002/mgg3.742

**Published:** 2019-05-15

**Authors:** Bo Xiang, Kezhi Liu, Minglan Yu, Xuemei Liang, Chaohua Huang, Jin Zhang, Wenying He, Wei Lei, Jing Chen, Xiaochu Gu, Ke Gong

**Affiliations:** ^1^ Department of Psychiatry, Nuclear Medicine and Molecular Imaging Key Laboratory of Sichuan Province Affiliated Hospital of Southwest Medical University Luzhou Sichuan Province China; ^2^ Medical Laboratory Center Affiliated Hospital of Southwest Medical University Luzhou Sichuan Province China; ^3^ Clinical Laboratory Suzhou Guangji Hospital Suzhou Jiangsu Province China

**Keywords:** gene clusters, GWAS, hippocampus, immune system, insomnia

## Abstract

**Background:**

Previous studies have inferred a strong genetic component for insomnia. However, the etiology of insomnia is still unclear. The aim of the current study was to explore potential biological pathways, gene networks, and brain regions associated with insomnia.

**Methods:**

Using pathways (gene sets) from Reactome, we carried out a two‐stage gene set enrichment analysis strategy. From a large genome‐wide association studies (GWASs) of insomnia symptoms (32,155 cases/26,973 controls), significant gene sets were tested for replication in other large GWASs of insomnia complaints (32,384 cases/80,622 controls). After the network analysis of unique genes within the replicated pathways, a gene set analysis for genes in each cluster/module of the enhancing neuroimaging genetics through meta‐analysis GWAS data was performed for the volumes of the intracranial and seven subcortical regions.

**Results:**

A total of 31 of 1,816 Reactome pathways were identified and showed associations with insomnia risk. In addition, seven functionally and topologically interconnected clusters (clusters 0–6) and six gene modules (named Yellow, Blue, Brown, Green, Red, and Turquoise) were associated with insomnia. Moreover, significant associations were detected between common variants of the genes in Cluster 2 with hippocampal volume (*p* = 0.035; family wise error [FWE] correction) and the red module with intracranial volume (*p* = 0.047; FWE correction). Functional enrichment for genes in the Cluster 2 and the Red module revealed the involvement of immune responses, nervous system development, NIK/NF‐kappaB signaling, and I‐kappaB kinase/NF‐kappaB signaling. Core genes (*UBC*, *UBB*, and *UBA52*) in the interconnected functional network were found to be involved in regulating brain development.

**Conclusions:**

The current study demonstrates that the immune system and the hippocampus may play central roles in neurodevelopment and insomnia risk.

## 1 INTRODUCTION

The prevalence of insomnia is approximately 6%–10% in the general population (Morin & Jarrin, 2013). Several studies have identified an association between insomnia and a multitude of mental health issues, including post‐traumatic stress disorder (PTSD)(Yehuda et al., 2015) and suicide (Fernandez‐Mendoza & Vgontzas, 2013), as well as adverse long‐term health problems, including diabetes (Anothaisintawee, Reutrakul, Van Cauter, & Thakkinstian, 2016) and cardiovascular disease (Jackson, Redline, & Emmons, 2015). The need for further research into the etiology of insomnia is therefore indicated.

Twin studies have revealed that sleep characteristics and insomnia are highly heritable, with a heritability rate ranging from 22% to 59% in adults, and 14% to 71% in children (Hublin, Partinen, Koskenvuo, & Kaprio, 2011; Wing et al., 2012). Previous genome‐wide association studies (GWASs) have revealed a number of susceptible genetic variants (particularly single nucleotide polymorphisms [SNPs]) involved in the development of insomnia symptoms (Lane et al., 2017) and insomnia complaints (Hammerschlag et al., 2017). However, these results from GWASs do not directly provide any functional information on the mapped variants, and can barely help understand the biological mechanism of insomnia. Meanwhile, it is likely that many more common variants are linked to insomnia but have not achieved genome‐wide significance in GWASs because of small effect size or insufficient sample size. Several studies have indicated that the weakly associated variants may provide important information regarding the biological basis of disease when such variants cluster within a common functional module or pathway (Jia et al., 2012; Xiang et al., 2018). In addition, numerous methodologies have been developed to analyze associations between genes and gene pathways with a risk of disease development. For example, the gene set enrichment analysis (GSEA) was originally designed to handle and analyze gene data on genome‐wide expression; this analysis could currently be used in a common pathway‐based analysis. Wang et al. (Wang, Li, & Bucan, 2007) developed a modified version of the GSEA in 2007 to analyze genome‐wide SNP associations, and the analytic algorithm could identify the combined SNP/gene effects on interactions of multiple genetic markers of a disease. Thus, this algorithm may be used to explore the biological functions and mechanisms of genes and gene pathways at the system level. There are advantages to extending the pathway‐based approach into molecular networks and co‐expression network to reveal the true biology of insomnia. A molecule network analysis could identify the molecular network that interacts with biomolecules, such as genes, proteins, metabolites, etc., in various forms (e.g., in protein–protein interactions, gene regulation, and functional interactions). Notably, previous studies have shown that genes related with the same or similar disorders tend to cluster in the same gene network by means of physical or functional clusters (Feldman, Rzhetsky, & Vitkup, 2008; Oti & Brunner, 2007). Thus, further study of GWAS data using newly developed tools and software could help identify genes and gene pathways in insomnia development or risk.

For genetic disorders, the most harmful mutation may exist in all the cells of our body, but it only frequently occurs in a few tissues because the mutated protein has different functions within these tissues or has different tissue‐specific interacting proteins (Magger, Waldman, Ruppin, & Sharan, 2012). Some studies have found that the perturbation of a gene, or an interaction between two gene products, can lead to the disruption of a protein interaction network (Zhong et al., 2009). We do not know whether general protein–protein interactions still exist in our target tissue or are blocked by some tissue‐specific factors. Thus, in this study, we added the brain tissue‐specific co‐expression network, which could directly provide brain‐related information, to better understand the pathogenesis of insomnia.

In the present study, we assessed and systematically analyzed multiple results of GWASs to increase statistical power and investigate potential biological pathways, gene networks, and brain regions associated with insomnia.

## 2 MATERIALS AND METHODS

### 2.1 Ethical compliance

The ethics approval of insomnia studies can be found in the original articles (Hammerschlag et al., 2017; Lane et al., 2017).

### 2.2 Analytic flowchart

**Figure 1:**
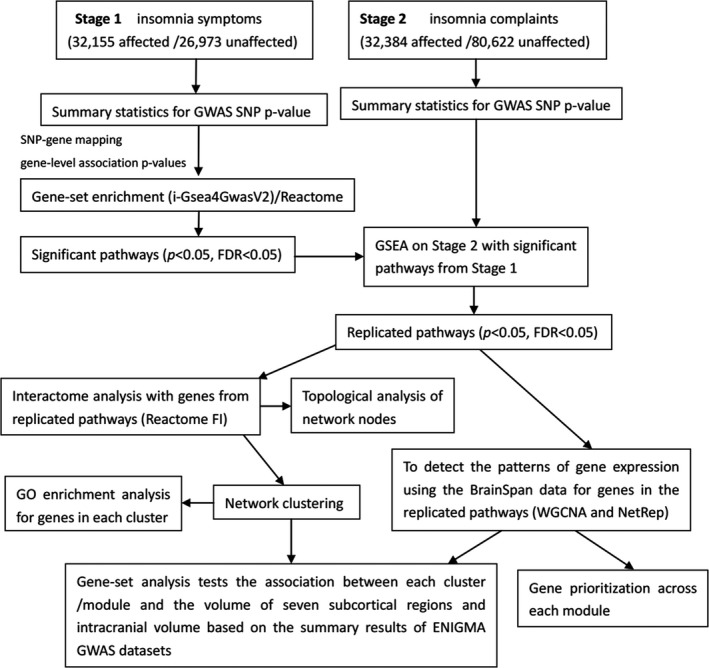
Illustration of our data analysis approach. GWAS, genome‐wide association study; FDR, false discovery rate; SNP, single‐nucleotide polymorphism; GSEA, gene‐set enrichment analysis; GO, gene ontology; WGCNA, weighted gene co‐expression network analysis; ENIGMA, enhancing neuroimaging genetics through meta‐analysis; NetRep, Fast permutation procedure for testing network module replication

In the present study, main analyses included: (a) integrate the summary statistics from insomnia symptoms, insomnia complaints and the Reactome pathway database to identify significant pathways; (b) The use of genes in the significant pathways to construct the functional interacting networks, gene co‐expression networks, and functional clusters/modules; (c) An analysis of the gene set association in each cluster/module with the volume of seven subcortical regions (accumbens, amygdala, caudate, hippocampus, pallidum, putamen, and thalamus) and intracranial volume (ICV) (Hibar et al., 2015). The schematic of our analytic approach is shown in Figure 1.

### 2.3 Retrieval of summary statistics for insomnia GWAS data

Summary statistics for GWAS data were obtained from subjects experiencing insomnia symptoms (*n* = 32,155) and controls (*n* = 26,973) (Lane et al., 2017), and those reporting insomnia complaints (*n* = 32,384) and controls (*n* = 80,622) (Hammerschlag et al., 2017). To assess insomnia symptoms, subjects were asked, “Do you have trouble falling asleep at night or do you wake up in the middle of the night?” with responses “never/rarely,” “sometimes,” “usually” and “prefer not to answer.” Based on response, subjects were dichotomized into controls (“never/rarely”) and cases (“usually”). GWAS analyses were run for insomnia symptoms using linear/logistic regression, with sex, age, 10 principal components and genotyping array as covariates (Lane et al., 2017). To assess insomnia complaints, subjects were asked, “Do you have trouble falling asleep at night or do you wake up in the middle of the night?” with four possible responses: “never/rarely,” “sometimes,” “usually,” or “prefer not to answer.” Responses of “usually” were considered complaints, while responses of “never/rarely” or “sometimes” were analyzed as controls. Genome‐wide association studies analyses were run for insomnia complaints, which are predictive of insomnia disorder with little confounding from comorbidity, using logistic regression adjusting for sex, age, top five principal components, and genotyping array (Hammerschlag et al., 2017).

### 2.4 Retrieval of enhancing neuroimaging genetics through meta‐analysis GWAS data

Summary datasets on GWASs of enhancing neuroimaging genetics through meta‐analysis (ENIGMA) conducted by Hibar et al. (Hibar et al., 2015) were downloaded. The GWASs included 13,171 samples from subjects of European ancestry and identified common genetic variants contributing to the volume differences in seven subcortical regions and to ICV. The data were then analyzed using various software programs (see below for more details).

### 2.5 Pathway‐based analysis

i‐Gsea4GwasV2 software (http://gsea4gwas-v2.psych.ac.cn/) (Zhang, Chang, Guo, & Wang, 2015) was applied to the Reactome pathway dataset for the summary statistics from insomnia symptoms/complaints, to perform a pathway‐based GSEA. The analysis procedure was as follows: (a) The program obtained a gene across the max‐log (*p*‐value) from all SNPs within a 20‐kb interval; (b) Genes were ranked via the *p*‐value for the association test; (c) The list of ranked genes was used to calculate enrichment scores (ES) of the pathways, and the ES showed a trend in which the genes in the pathways were located from the top of the list of entire ranked genes; (d) A phenotype label permutation and normalization were performed to obtain the ES distribution, and correct the gene and gene set variations; (e) False discovery rate (FDR) was performed for multiple tests based on the ES distributions generated by permutation tests.

### 2.6 Network and network clustering analyses of genes associated with insomnia

A pathway‐based analysis can identify genes and pathways that are associated with insomnia. However, obtaining the genes in each pathway may not allow for sufficient comprehension of the topological and functional relationships of each gene in the pathway. It may be necessary for some genes to form a cluster to construct molecular networks and modulate the biological mechanism of a disease such as insomnia. Therefore, the Reactome functional interactions (FI) software (Wu, Feng, & Stein, 2010) was used to form gene and gene pathway networks. The Reactome FI dataset contained the Reactome, Panther, BioCyc, kyoto encyclopedia of genes and genomes, Pathway Interaction Database, Cancer Cell Map, and other pair‐wise interactions collected from physical protein–protein interactions, protein domain–domain interactions, gene co‐expression data, gene ontology (GO) annotation, and text mining.

A large molecular network may comprise diverse small modules in which the edges among nodes within a module are tight, while the connections among modules are loose. Therefore, a network cluster analysis was used to identify the architecture of each module in a large molecular network based on the algorithm as described in a prior study (Girvan & Newman, 2002). Each cluster may be involved in different biological functions and provide novel insights into the understanding of the pathogenesis of insomnia (Xiang et al., 2018).

### 2.7 Gene and pathway prioritization across network topology

Network topology can provide vital information to the understanding of the structure of a network across identification of the core node (gene). The CentiScaPe 2.0 program (Scardoni, Petterlini, & Laudanna, 2009) was used to research topological characteristics of the networks originating from the gene networks. Two key node centrality measures for gene networks, degree and betweenness, were addressed due to their importance in biological networks as drivers for gene/protein essentiality (Yu, Kim, Sprecher, Trifonov, & Gerstein, 2007). Genes from significant pathways were imported into Reactome FI networks and then the core nodes (genes) were analyzed based on the two node centrality measurements.

### 2.8 The construction of gene co‐expression networks in 15 brain regions

The gene co‐expression networks in 15 brain regions were constructed based on BrainSpan whole‐genome transcriptomic data, collected by RNA‐seq (http://www.brainspan.org/). The 15 brain regions included 11 neocortical regions (primary auditory cortex [A1C], primary motor cortex [M1C], primary somatosensory cortex [S1C], primary visual cortex [V1C], dorsolateral prefrontal cortex [DFC], medial prefrontal cortex [MFC], orbital frontal cortex [OFC], ventrolateral prefrontal cortex [VFC], inferolateral temporal cortex [ITC], superior temporal cortex [STC], inferior parietal cortex [IPC]), the striatum (STR), the hippocampus (HIP), the thalamus (MD), and the amygdaloid (AMY) (Table S1). Gene expression was defined by a normalized reads per kilobase million (RPKM) value of 1 in at least one region at one time point for 80% of the available samples (Parikshak et al., 2013). The R package WGCNA (Langfelder & Horvath, 2008) was used to construct gene co‐expression networks. To further explore which co‐expression modules is preservation, we used the NetRep program (Ritchie et al., 2016) to replicate these modules in each brain region.

### 2.9 Protein–protein interaction analysis

Prior studies have identified disease‐associated genes that tend to interact more with each other than with random proteins in the protein–protein interaction (PPI) network, while protein‐coding genes situated at the same genomic locus, tend to interact within the PPI network (Oti, Snel, Huynen, & Brunner, 2006). In the current study, we carried out a permutation test using the Disease Association Protein‐Protein Link Evaluator (DAPPLE, http://www.broadinstitute.org/mpg/dapple/dapple.php) (Rossin et al., 2011) and evaluated whether genes in each cluster had significant physical interactions with each other or with other proteins across the network connectivity parameters (degree and number of edges) versus random networks with a similar size and degree distribution.

### 2.10 Gene set association analysis of each cluster with seven subcortical regions

To determine associations between genes in each cluster with the volume of seven subcortical regions (accumbens, amygdala, caudate, hippocampus, pallidum, putamen, and thalamus) and total ICV (Hibar et al., 2015), all SNPs mapped to genes in each cluster were extracted, and obtained cumulative evidence for each cluster with the volume of the subcortical regions. A gene set analysis of the ENIGMA GWAS dataset (Hibar et al., 2015) was performed with magma software (de Leeuw, Mooij, Heskes, & Posthuma, 2015). Individual SNPs in each gene were analyzed and the *p*‐value of the resulting SNPs was integrated into a statistical gene test. The linkage disequilibrium among the SNPs in the gene was then estimated according to reference data with similar ancestry (1000 genomes) and the *p*‐value of a gene during the gene set analysis.

### 2.11 Gene ontology enrichment analysis

ConsensusPathDB (Kamburov, Stelzl, Lehrach, & Herwig, 2013) was used to perform GO enrichment analysis of genes in each module. A hypergeometric test implemented in ConsensusPathDB computed the enrichment *p*‐value, followed by a FDR correction *p* < 0.05 for multiple testing.

## 3 RESULTS

**Figure 2:**
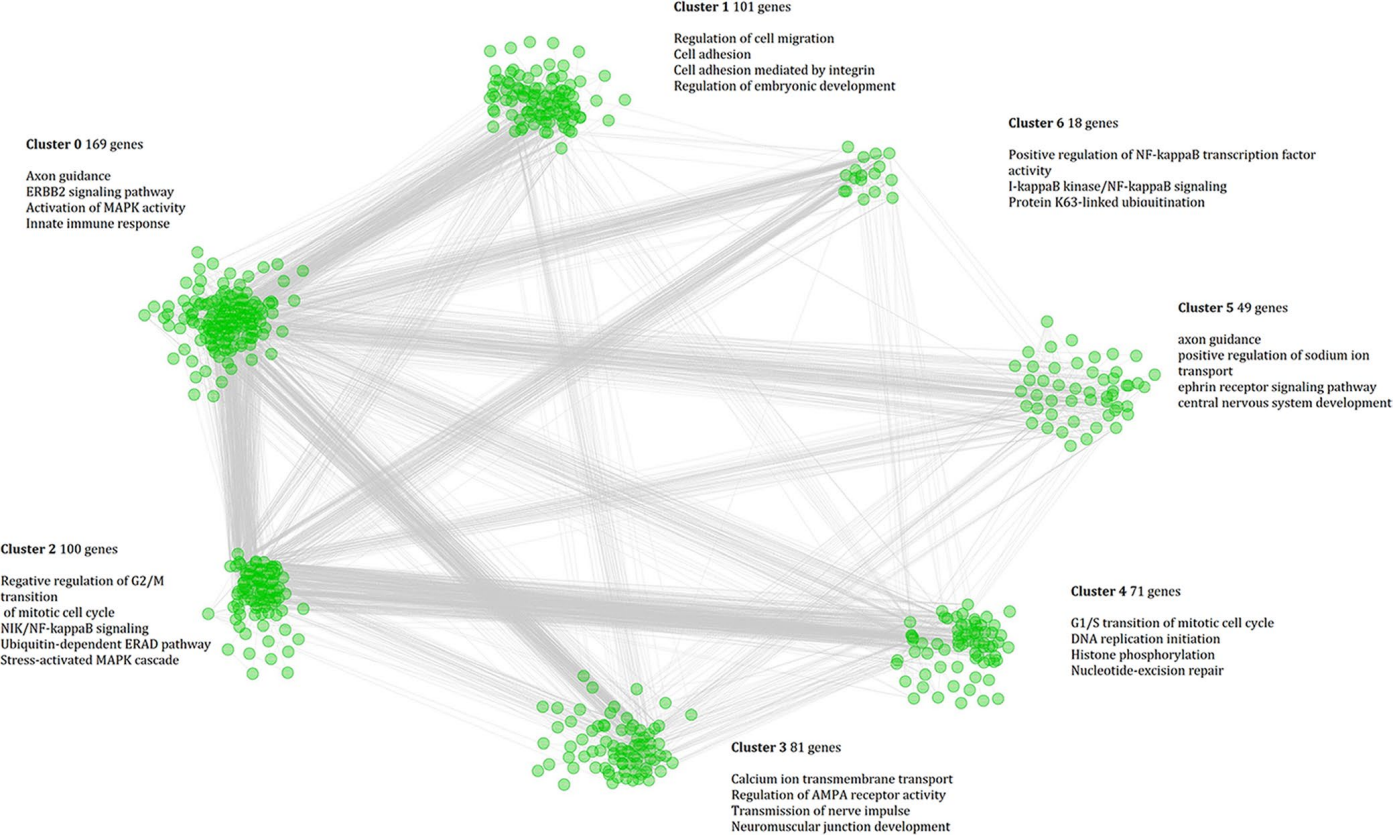
Functionally interacting network modules. These were constructed from genes in the significant pathways, and functional interactions between the genes from significant pathways were analyzed and clustered using the Reactome FI tool and visualized in Cytoscape. Genes are represented as nodes, while the interactions between genes are represented as edges. The parent network was further analyzed to yield sub‐network clusters, and each cluster is separately shown and the color encoded for clarity

A pathway‐based analysis identified 41 of the 1,816 Reactome pathways for insomnia symptoms to have a gene set enrichment (*p* < 0.05, *p*_FDR_ < 0.05), and 31 of these 41 pathways were further identified for insomnia complaints (*p* < 0.05, *p*_FDR_ < 0.05; Table 1). Thirty‐one significant pathways included 634 genes (Table S2), which were imported into the Reactome FI program, and a larger molecular network of 598 genes was obtained. This network was then clustered into several sub‐networks. Seven clusters (clusters 0–6) containing ≥18 gene members (Figure 2; Table S3) were identified, and a series of gene interactions through the edge connection of the genes (nodes) within each cluster (Figure 2) was detected. *UBC*, *UBB*, *UBA52* (degree) and *UBC*, *EP300*, *PRKACA*, *MAPK1*, and *SRC* (betweenness) were identified as core genes in the linking of clusters (Table S4). Using the GO dataset, an enrichment analysis for genes in each cluster was conducted and several interesting biological pathways were discovered, such as the axon guidance and ERBB2 signaling pathway (Clusters 0 and 5), the NIK/NF‐kappaB signaling pathway, the innate immune response activating cell surface receptor signaling pathway and stress‐activated MAPK cascade (Cluster 2), and the calcium ion transmembrane transport (Cluster 3). A full list of all significantly enriched biological processes with an FDR < 0.001 is shown in the Table S5. The gene set analysis also showed significant associations among common variants of the genes in Cluster 2 with the hippocampal volume (*p* = 0.035, family wise error correction).

**Table 1:** Significant pathways enriched for association with insomnia

Pathways	*p*	FDR
Hedgehog ligand biogenesis	0.007	0.016
Ubiquitin‐dependent degradation of Cyclin D1	0.017	0.016
Ubiquitin‐dependent degradation of Cyclin D	0.017	0.016
Phase 2—plateau phase	0.005	0.017
Cross‐presentation of soluble exogenous antigens (endosomes)	0.011	0.017
GPVI‐mediated activation cascade	0.004	0.018
L1CAM interactions	0.003	0.019
Cardiac conduction	0.003	0.019
Cyclin E associated events during G1/S transition	0.011	0.019
Negative regulation of the PI3K/AKT network	0.005	0.020
Regulation of ornithine decarboxylase (ODC)	0.009	0.020
Orc1 removal from chromatin	0.011	0.022
Switching of origins to a post‐replicative state	0.011	0.022
Cyclin A:Cdk2‐associated events at S phase entry	0.010	0.025
CDT1 association with the CDC6:ORC:origin complex	0.011	0.026
G1/S transition	0.003	0.028
CDK‐mediated phosphorylation and removal of Cdc6	0.010	0.028
Hh mutants abrogate ligand secretion	0.025	0.031
E2F mediated regulation of DNA replication	0.022	0.031
Constitutive signaling by aberrant PI3K in cancer	0.001	0.033
Removal of licensing factors from origins	0.011	0.033
Hh mutants that do not undergo autocatalytic processing are degraded by ERAD	0.027	0.034
PI5P, PP2A and IER3 regulate PI3K/AKT signaling	0.001	0.035
Phase 1—inactivation of fast Na+ channels	0.017	0.037
Ubiquitin mediated degradation of phosphorylated Cdc25A	0.034	0.038
p53‐independent DNA damage response	0.034	0.038
p53‐independent G1/S DNA damage checkpoint	0.034	0.038
Muscle contraction	0.001	0.038
C‐type lectin receptors (CLRs)	0.002	0.041
Phase 0—rapid depolarisation	0.020	0.042
Non‐integrin membrane‐ECM interactions	0.029	0.046

Note

Abbreviation: FDR, false discovery rate.

The co‐expression network analysis found that 532 out of 634 genes were co‐expressed in six gene modules (named Yellow, Blue, Brown, Green, Red, and Turquoise; Table S6). Based on the NetRep program, Yellow, Blue, Red, and Turquoise modules were replicated in 15 brain regions (Table S7). The gene set analysis also identified significant associations among common variants of genes in the red module with ICV (*p* = 0.047, family wise error correction). Functional enrichment of these genes in the red module revealed interesting biological processes, such as immune response, nervous system development, axon guidance, NIK/NF‐kappaB signaling, and I‐kappaB kinase/NF‐kappaB signaling (Table S8). In addition, these genes were found to be highly enriched in the thick corpus callosum (*p* = 0.0001; *p*_adj_ = 0.0083).

In order to explore the PPI network for genes in the red module, 54 genes were imported into the InWeb PPI network. The resulting network was significantly different random networks (Figure S1), for example, there were 37 direct edges in the network compared with only 13.91 edges expected by chance alone (*p* < 0.001). Moreover, the observed average connectivity per gene was 2.74 compared with an expected 1.57 from random networks (*p* < 0.001). These findings suggest that the constructed networks did not occur by chance alone.

## 4 DISCUSSION

Despite the recent success of a large GWAS in the identification of several common genetic variations associated with insomnia, the etiology of insomnia remains poorly understood. Thus, the current study integrated the GWAS data on insomnia with pathway data, gene functional interaction networks, and co‐expression networks to determine the potential causes of insomnia. The analyses obtained 31 core human pathways as being most etiologically relevant to insomnia or insomnia susceptibility. Functional and bioinformatic studies are needed to further understand the relationship between these 31 core human pathways and insomnia.

In the present study, pathway and network analyses revealed that *NF‐κB* and immune responses play important roles in the regulation of biological mechanisms associated with insomnia. During sleep disturbance, the sympathetic nervous system releases norepinephrine into primary and secondary lymphoid organs and stimulates the adrenal gland to release epinephrine into the systemic circulation (Irwin & Cole, 2011; Irwin & Opp, 2017). Neuromodulators, such as epinephrine, acidic fibroblast growth factor, and epidermal growth factor, stimulate leukocyte adrenergic receptors to further activate *NF‐κB* which subsequently regulates immune response gene transcription, such as *TNF*, *IL‐6*, and *IL‐1*, ultimately leading to the translation and production of pro‐inflammatory cytokines that serve to regulate the inflammatory response (Cole, 2010; Irwin & Opp, 2017; Karin, 2006). Furthermore, pro‐inflammatory cytokines (such as *TNF* and *IL‐6*) act within a complex biochemical network, leading to the stimulation of *NF‐κB* and the modification of the transcription of hundreds of gene products, each of which promotes sleep (Krueger, 2008), and promotes a positive feedback loop (Irwin & Opp, 2017).

A large molecular network constructed from 598 genes that may play a key role in the etiology of insomnia was identified. Moreover, *UBC*, *UBB*, *UBA52* were identified as core genes that link seven clusters and included in the Cluster 2. Several studies have found that ubiquitin (Ub) has diverse functions in eukaryotic cells, including the targeting of proteins for modulation of signaling pathways, receptor endocytosis, and proteasomal degradation (Ravid & Hochstrasser, 2008). There are two classes of UB genes: monomeric Ub‐ribosomal fusion genes (*UBA52* and *UBA80*) and stress‐inducible polyubiquitin genes (*UBB* and *UBC*) (Komander, Clague, & Urbé, 2009). UB genes are known to play key roles during neuronal development, including neuritogenesis, neurogenesis, and synaptogenesis (Kawabe & Brose, 2011). Ryu et al. (Ryu, Park, & Ryu, 2014) found that neuronal morphology, neurite outgrowth, and synaptic development were impaired in UBB^−/−^ neurons. *UBC* and *UBA52* may play a critical role in compensating for disruption of *UBB* in neurons and astrocytes (Sinnar et al., 2011), and disruption of the *UBB* gene can cause hypothalamic neurodegeneration and sleep abnormalities in mice (Ryu et al., 2010). These findings suggest that immune response gene which interacts with *UBC*, *UBB*, and *UBA52* regulate neuronal development and sleep.

In addition to the identification of pathways involved in the immune response, which may form a positive feedback loop with neuronal development to influence insomnia, the genes in Cluster 2/red module were associated with the hippocampal/ICV, Guzman‐Marin et al. (Guzman‐Marin et al., 2005) and Hairston et al. (Hairston et al., 2005) also provided the evidence of suppressed hippocampal neurogenesis in sleep‐deprived rats. Riemann et al. (Riemann et al., 2007) and Winkelan et al. (Winkelman et al., 2010) found that the hippocampal volume was significantly reduced in individuals with primary insomnia (PI) compared to good/normal sleepers. Chao et al. (Chao, Mohlenhoff, Weiner, & Neylan, 2014) also identified that poorer subjective sleep quality was associated with reduced total cortical volumes. The above results indicate that the inflammatory biological pathways may regulate the development of hippocampus and further lead to insomnia.

While the current study contributes novel data to our understanding of the etiology of insomnia, there are some limitations. First, analyses were conducted on genes collected from insomnia symptoms in a European population and may need to replicate in other samples, or across other sleep phenotypes. Second, the insomnia symptoms/complaints are the subjective nature of the insomnia item, and not a diagnosis. Third, the current study does not explore how the relationship among *UBC*, *UBB*, *UBA52*, *TNF‐kB*, and hippocampus may modulate sleep.

In summary, through an integrated analysis of genetic data from the summary statistics for insomnia GWASs, pathway and brain co‐expression networks, the current results indicate that dysregulation of genes involved in the immune system has an important role in the pathogenesis of insomnia, and may provide a theoretical basis for future research.

## CONFLICT OF INTEREST

None declared.
