# Supplementary material for: Systematic genetic analyses of GWAS data reveal an association between the immune system and insomnia
Source: Mol Genet Genomic Med. 2019 May 15;7(7):e00742. doi: 10.1002/mgg3.742 (PMC6625127; doi:10.1002/mgg3.742)
Supplement: Supplementary file 1 [file MGG3-7-e00742-s001.docx]

**Supplementary Figure 1.** Protein-Protein interaction analysis for red module


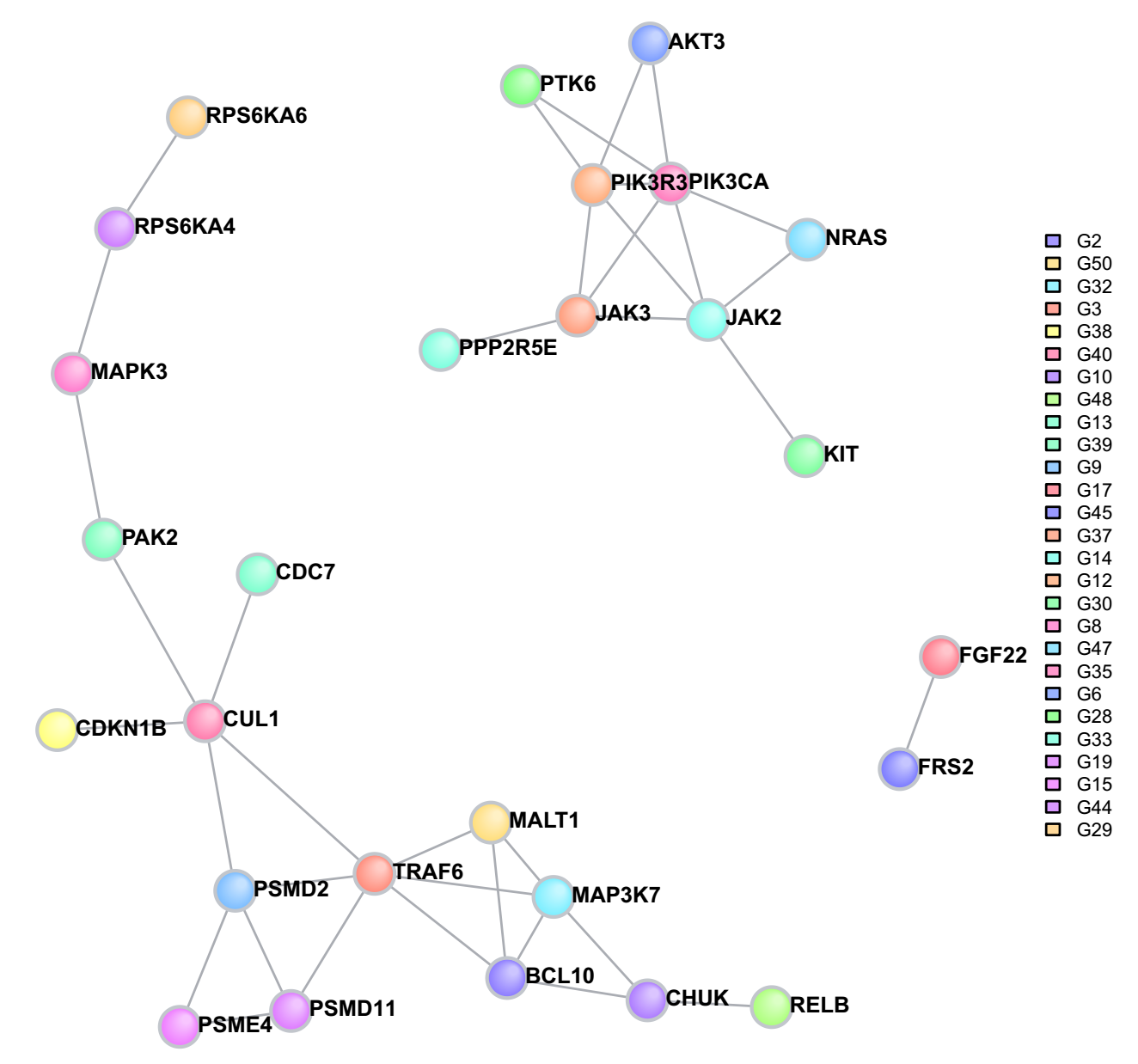


**Supplementary Table1** 15 brain regions were used to compute the

coexpression network

| STR | Striatum |  |
| --- | --- | --- |
| MD | Mediodorsal nucleus of thalamus |  |
| AMY | Amygdaloid complex |  |
| HIP | Hippocampus |  |
| A1C | Primary auditory cortex | Neocortical regions |
| M1C | Primary motor cortex |  |
| S1C | Primary somatosensory cortex |  |
| V1C | Primary visual cortex |  |
| DFC | Dorsolateral prefrontal cortex |  |
| MFC | Anterior (rostral) cingulate (medial prefrontal) cortex |  |
| OFC | Orbital frontal cortex |  |
| VFC | Ventrolateral prefrontal cortex |  |
| ITC | Inferolateral temporal cortex |  |
| STC | Posterior (caudal) superior temporal cortex |  |
| IPC | Posteroinferior (ventral) parietal cortex |  |

**Supplementary Table2.** 31 significant pathways included 634 genes

| DERL2 | PSME1 | ERBB3 | NRG4 | PPP2R3B | TTR | CACNB4 | NUMB |
| --- | --- | --- | --- | --- | --- | --- | --- |
| DHH | PSME2 | ERBB4 | PDGFA | PRIM1 | VTN | CACNG1 | RANBP9 |
| ERLEC1 | PSME3 | EREG | PDGFB | PRIM2 | AZIN1 | CACNG2 | RDX |
| IHH | PSME4 | FGF1 | PDGFRA | RRM2 | NQO1 | CACNG3 | RPS6KA1 |
| OS9 | PSMF1 | FGF10 | PDGFRB | TFDP1 | OAZ1 | CACNG4 | RPS6KA2 |
| PSMA1 | RPS27A | FGF16 | PIK3CA | TK2 | OAZ2 | CACNG5 | RPS6KA3 |
| PSMA2 | SEL1L | FGF17 | PIK3CB | TYMS | OAZ3 | CACNG6 | RPS6KA4 |
| PSMA3 | SHH | FGF18 | PIK3CD | ACTN1 | ODC1 | CACNG7 | RPS6KA6 |
| PSMA4 | SYVN1 | FGF19 | PIK3R1 | AGRN | ADAM17 | CACNG8 | SDCBP |
| PSMA5 | UBA52 | FGF2 | PIK3R2 | CASK | DISP2 | CALM1 | SH3GL2 |
| PSMA6 | UBB | FGF20 | PIK3R3 | DAG1 | GPC5 | CAMK2A | SHTN1 |
| PSMA7 | UBC | FGF22 | PIP4K2A | DDR1 | HHAT | CAMK2B | SPTA1 |
| PSMA8 | VCP | FGF23 | PIP4K2B | DDR2 | NOTUM | CAMK2D | SPTAN1 |
| PSMB1 | CCNA1 | FGF3 | PIP4K2C | DMD | P4HB | CAMK2G | SPTB |
| PSMB10 | CCNA2 | FGF4 | PIP5K1A | HSPG2 | SCUBE2 | CLIC2 | SPTBN1 |
| PSMB11 | CDC6 | FGF5 | PIP5K1B | ITGA2 | ABCC9 | CORIN | SPTBN2 |
| PSMB2 | CDK2 | FGF6 | PIP5K1C | ITGA6 | AHCYL1 | DMPK | SPTBN4 |
| PSMB3 | CDKN1A | FGF7 | PPP2CA | ITGAV | AKAP9 | FGF11 | SPTBN5 |
| PSMB4 | CDKN1B | FGF8 | PPP2CB | ITGB1 | ASPH | FGF12 |  |
| PSMB5 | CDT1 | FGF9 | PPP2R1A | ITGB3 | ATP1A1 | FGF13 |  |
| PSMB6 | FZR1 | FGFR1 | PPP2R1B | ITGB4 | ATP1A2 | FGF14 |  |
| PSMB7 | MCM2 | FGFR2 | PPP2R5A | ITGB5 | ATP1A3 | FKBP1B |  |
| PSMB8 | MCM3 | FGFR3 | PPP2R5B | LAMA1 | ATP1A4 | FXYD1 |  |
| PSMB9 | MCM4 | FGFR4 | PPP2R5C | LAMA2 | ATP1B1 | FXYD2 |  |
| PSMC1 | MCM5 | FRS2 | PPP2R5D | LAMA3 | ATP1B2 | FXYD3 |  |
| PSMC2 | MCM6 | FYN | PPP2R5E | LAMA4 | ATP1B3 | FXYD4 |  |
| PSMC3 | MCM7 | GAB1 | PTPN11 | LAMA5 | ATP2A1 | FXYD6 |  |
| PSMC4 | MCM8 | GRB2 | SRC | LAMB1 | ATP2A2 | FXYD7 |  |
| PSMC5 | ORC1 | HBEGF | TRAT1 | LAMB2 | ATP2A3 | GATA4 |  |
| PSMC6 | ORC2 | IER3 | VAV1 | LAMB3 | ATP2B1 | HIPK1 |  |
| PSMD1 | ORC3 | INS | ATM | LAMC1 | ATP2B2 | HIPK2 |  |
| PSMD10 | ORC4 | INSR | CDC25A | LAMC2 | ATP2B3 | ITPR1 |  |
| PSMD11 | ORC5 | IRS1 | CHEK1 | LAMC3 | ATP2B4 | ITPR2 |  |
| PSMD12 | ORC6 | IRS2 | CHEK2 | NRXN1 | CACNA1C | ITPR3 |  |
| PSMD13 | RB1 | KIT | CCNB1 | NTN4 | CACNA1D | KAT2B |  |
| PSMD14 | AKT1 | KITLG | CCNE1 | PRKCA | CACNA1F | KCND1 |  |
| PSMD2 | BTC | KL | CDC45 | SDC1 | CACNA1S | KCND2 |  |
| PSMD3 | CD19 | KLB | CDK1 | SDC2 | CACNA2D1 | KCND3 |  |
| PSMD4 | CD28 | LCK | DHFR | SDC3 | CACNA2D2 | KCNE1 |  |
| PSMD5 | CD80 | MAPK1 | E2F1 | SDC4 | CACNA2D3 | KCNE2 |  |
| PSMD6 | CD86 | MAPK3 | FBXO5 | TGFB1 | CACNA2D4 | KCNE3 |  |
| PSMD7 | EGF | NRG1 | PCNA | THBS1 | CACNB1 | KCNE4 |  |
| PSMD8 | EGFR | NRG2 | POLA1 | TNC | CACNB2 | KCNE5 |  |
| PSMD9 | ERBB2 | NRG3 | POLA2 | TRAPPC4 | CACNB3 | KCNH2 |  |
| KCNIP1 | SCN3A | MYL3 | PKMYT1 | SHC1 | MAP3K7 | AP2A1 |  |
| KCNIP2 | SCN3B | MYL4 | PTK6 | SYK | NFATC1 | AP2A2 |  |
| KCNIP3 | SCN4A | MYL5 | SKP1 | VAV2 | NFATC2 | AP2B1 |  |
| KCNIP4 | SCN4B | MYL6 | SKP2 | VAV3 | NFATC3 | AP2M1 |  |
| KCNJ11 | SCN5A | MYL6B | WEE1 | GMNN | NFKB1 | AP2S1 |  |
| KCNJ12 | SCN7A | MYL7 | CDC25B | MCM10 | NFKB2 | CD24 |  |
| KCNJ14 | SCN8A | MYL9 | AKT2 | PHLPP1 | NFKBIA | CHL1 |  |
| KCNJ2 | SCN9A | MYLK | AKT3 | PHLPP2 | NRAS | CLTA |  |
| KCNJ4 | SLC8A1 | MYLPF | CDC42 | PTEN | PAK1 | CNTN1 |  |
| KCNK1 | SLC8A2 | NEB | CLEC1B | THEM4 | PAK2 | CNTN2 |  |
| KCNK10 | SLC8A3 | PXN | CSF2 | TRIB3 | PAK3 | CNTN6 |  |
| KCNK12 | SLN | SORBS1 | CSF2RA | CD207 | PPP3CA | CNTNAP1 |  |
| KCNK13 | SRI | SORBS3 | CSF2RB | FCGR1A | PPP3CB | CSNK2A1 |  |
| KCNK15 | STIM1 | TCAP | FCER1G | FCGR1B | PPP3R1 | CSNK2A2 |  |
| KCNK16 | TBX5 | TLN1 | G6B | MRC1 | PRKACB | CSNK2B |  |
| KCNK17 | TNNI3 | TMOD1 | GAB2 | MRC2 | PRKACG | DCX |  |
| KCNK18 | TRDN | TMOD2 | GP6 | BCL10 | PRKCD | DLG1 |  |
| KCNK2 | TRPC1 | TMOD3 | IL2 | BTRC | PYCARD | DLG3 |  |
| KCNK3 | WWTR1 | TMOD4 | IL2RA | CARD11 | RAF1 | DLG4 |  |
| KCNK4 | ACTA2 | TNNC1 | IL2RB | CARD9 | RELA | DNM1 |  |
| KCNK5 | ACTG2 | TNNC2 | IL2RG | CASP8 | RELB | DNM2 |  |
| KCNK6 | ACTN2 | TNNI1 | IL3 | CCL17 | RPS6KA5 | DNM3 |  |
| KCNK7 | ACTN3 | TNNI2 | IL3RA | CCL22 | TAB1 | DPYSL2 |  |
| KCNK9 | ANXA1 | TNNT1 | IL5 | CD209 | TAB2 | EPHB2 |  |
| KCNQ1 | ANXA2 | TNNT2 | IL5RA | CDC34 | TAB3 | EZR |  |
| NKX2-5 | ANXA6 | TNNT3 | JAK1 | CHUK | TRAF6 | HSPA8 |  |
| NOS1 | CALD1 | TPM1 | JAK2 | CLEC4A | UBA3 | ITGA10 |  |
| NPPA | CAV3 | TPM2 | JAK3 | CLEC4C | UBE2D1 | ITGA2B |  |
| NPPC | DES | TPM3 | LAT | CLEC4D | UBE2D2 | ITGA5 |  |
| NPR1 | DYSF | TPM4 | LCP2 | CLEC4E | UBE2M | ITGA9 |  |
| NPR2 | ITGA1 | TRIM72 | LYN | CLEC6A | UBE2N | KCNQ2 |  |
| ORAI1 | LMOD1 | TTN | PDPK1 | CLEC7A | UBE2V1 | KCNQ3 |  |
| PLN | MYBPC1 | VCL | PDPN | CREBBP | CDC7 | KIF4A |  |
| PRKACA | MYBPC2 | VIM | PIK3CG | EP300 | DBF4 | KIF4B |  |
| RANGRF | MYBPC3 | CCND1 | PIK3R5 | FBXW11 | POLE | L1CAM |  |
| RYR1 | MYH11 | CCNE2 | PIK3R6 | HRAS | POLE2 | MAP2K1 |  |
| RYR2 | MYH3 | CCNH | PLCG2 | ICAM2 | RPA1 | MAP2K2 |  |
| RYR3 | MYH6 | CDK4 | PRKCZ | ICAM3 | RPA2 | MSN |  |
| SCN10A | MYH8 | CDK7 | PTPN6 | IKBKB | RPA3 | NCAM1 |  |
| SCN11A | MYL1 | CKS1B | RAC1 | IKBKG | RPA4 | NCAN |  |
| SCN1A | MYL10 | CUL1 | RAC2 | IL1B | ALCAM | NFASC |  |
| SCN1B | MYL12A | MAX | RHOA | KRAS | ANK1 | NRCAM |  |
| SCN2A | MYL12B | MNAT1 | RHOB | MALT1 | ANK2 | NRP1 |  |
| SCN2B | MYL2 | MYC | RHOG | MAP3K14 | ANK3 | NRP2 |  |

**Supplementary Table3.** 598 genes clustered into several sub-networks.

| **Module** | **0** | **1** | **2** | **3** | **4** | **5** | **6** | **7** | **8** | **9** |
| --- | --- | --- | --- | --- | --- | --- | --- | --- | --- | --- |
| Nodes | 169 | 101 | 100 | 81 | 71 | 49 | 18 | 4 | 3 | 2 |
| Node List | ADAM17 | ACTA2 | AZIN1 | ABCC9 | ATM | ALCAM | BCL10 | KCND2 | KCND1 | KCNK3 |
|  | AKT1 | ACTG2 | BTRC | AHCYL1 | CCNA1 | ANK1 | CARD11 | KCND3 | KCNIP2 | KCNK9 |
|  | AKT2 | ACTN1 | CCND1 | AKAP9 | CCNA2 | ANK2 | CARD9 | KCNIP1 | KCNIP4 |  |
|  | AKT3 | ACTN2 | CCNE2 | ASPH | CCNB1 | ANK3 | CASP8 | KCNIP3 |  |  |
|  | ATP1A1 | ACTN3 | CDC25A | ATP2A1 | CCNE1 | AP2A1 | CHUK |  |  |  |
|  | ATP1A2 | AGRN | CDC34 | ATP2A2 | CCNH | AP2A2 | HSPA8 |  |  |  |
|  | ATP1A3 | ANXA1 | CDKN1A | ATP2A3 | CDC25B | AP2B1 | IKBKB |  |  |  |
|  | ATP1A4 | ANXA2 | CDKN1B | ATP2B1 | CDC45 | AP2M1 | IKBKG |  |  |  |
|  | ATP1B1 | ANXA6 | CHEK2 | ATP2B2 | CDC6 | AP2S1 | IL1B |  |  |  |
|  | ATP1B2 | CALD1 | CKS1B | ATP2B3 | CDC7 | CD24 | MALT1 |  |  |  |
|  | ATP1B3 | CAV3 | CUL1 | ATP2B4 | CDK1 | CLTA | MAP3K7 |  |  |  |
|  | BTC | CHL1 | DERL2 | CACNA1C | CDK2 | CNTN1 | PYCARD |  |  |  |
|  | CD19 | CNTN6 | DHH | CACNA1D | CDK4 | CNTN2 | TAB1 |  |  |  |
|  | CD209 | DAG1 | DISP2 | CACNA1F | CDK7 | CNTNAP1 | TAB2 |  |  |  |
|  | CD28 | DES | ERLEC1 | CACNA1S | CDT1 | DCX | TAB3 |  |  |  |
|  | CD80 | DMD | FBXO5 | CACNA2D1 | CHEK1 | DNM1 | TRAF6 |  |  |  |
|  | CD86 | DYSF | FBXW11 | CACNA2D2 | CORIN | DNM2 | UBE2N |  |  |  |
|  | CDC42 | HSPG2 | FZR1 | CACNA2D3 | CREBBP | DPYSL2 | UBE2V1 |  |  |  |
|  | CLEC1B | ICAM2 | GMNN | CACNA2D4 | CSNK2A1 | INS |  |  |  |  |
|  | CLEC4C | ITGA1 | GPC5 | CACNB1 | CSNK2A2 | KCNQ2 |  |  |  |  |
|  | CLEC4D | ITGA10 | HHAT | CACNB2 | CSNK2B | KCNQ3 |  |  |  |  |
|  | CLEC4E | ITGA2 | ICAM3 | CACNB3 | DBF4 | KIF4A |  |  |  |  |
|  | CLEC6A | ITGA2B | IER3 | CACNB4 | DHFR | KIF4B |  |  |  |  |
|  | CLEC7A | ITGA5 | MAP3K14 | CACNG1 | E2F1 | L1CAM |  |  |  |  |
|  | CSF2 | ITGA6 | MYC | CACNG2 | EP300 | NCAM1 |  |  |  |  |
|  | CSF2RA | ITGA9 | NFKB1 | CACNG3 | GATA4 | NCAN |  |  |  |  |
|  | CSF2RB | ITGAV | NFKB2 | CACNG4 | HIPK1 | NFASC |  |  |  |  |
|  | DDR2 | ITGB1 | NFKBIA | CACNG5 | HIPK2 | NRCAM |  |  |  |  |
|  | EGF | ITGB3 | NOTUM | CACNG6 | KAT2B | NRP1 |  |  |  |  |
|  | EGFR | ITGB4 | NQO1 | CACNG7 | MAX | NRP2 |  |  |  |  |
|  | EPHB2 | ITGB5 | NUMB | CACNG8 | MCM10 | RANGRF |  |  |  |  |
|  | ERBB2 | KCNH2 | OAZ1 | CAMK2A | MCM2 | RPS6KA1 |  |  |  |  |
|  | ERBB3 | KCNJ12 | OAZ2 | CAMK2B | MCM3 | RPS6KA2 |  |  |  |  |
|  | ERBB4 | KCNJ2 | OAZ3 | CAMK2D | MCM4 | RPS6KA3 |  |  |  |  |
|  | EREG | KCNJ4 | ODC1 | CAMK2G | MCM5 | RPS6KA4 |  |  |  |  |
|  | EZR | LAMA1 | OS9 | CASK | MCM6 | RPS6KA5 |  |  |  |  |
|  | FCER1G | LAMA2 | P4HB | CLIC2 | MCM7 | RPS6KA6 |  |  |  |  |
|  | FCGR1A | LAMA3 | PAK2 | DLG1 | MCM8 | SCN1B |  |  |  |  |
|  | FGF1 | LAMA4 | PSMA1 | DLG3 | MNAT1 | SCN2B |  |  |  |  |
|  | FGF10 | LAMA5 | PSMA2 | DLG4 | NKX2-5 | SCN5A |  |  |  |  |
|  | FGF11 | LAMB1 | PSMA3 | DMPK | NPPA | SH3GL2 |  |  |  |  |
|  | FGF12 | LAMB2 | PSMA4 | FKBP1B | NPPC | SHTN1 |  |  |  |  |
|  | FGF13 | LAMB3 | PSMA5 | ITPR1 | NPR1 | SPTA1 |  |  |  |  |
|  | FGF14 | LAMC1 | PSMA6 | ITPR2 | NPR2 | SPTAN1 |  |  |  |  |
|  | FGF16 | LAMC2 | PSMA7 | ITPR3 | ORC1 | SPTB |  |  |  |  |
|  | FGF17 | LAMC3 | PSMA8 | KCNE1 | ORC2 | SPTBN1 |  |  |  |  |
|  | FGF18 | LMOD1 | PSMB1 | KCNE2 | ORC3 | SPTBN2 |  |  |  |  |
|  | FGF19 | MRC1 | PSMB10 | KCNJ11 | ORC4 | SPTBN4 |  |  |  |  |
|  | FGF2 | MRC2 | PSMB11 | KCNK5 | ORC5 | SPTBN5 |  |  |  |  |
|  | FGF20 | MYBPC1 | PSMB2 | KCNQ1 | ORC6 |  |  |  |  |  |
|  | FGF22 | MYBPC2 | PSMB3 | NOS1 | PCNA |  |  |  |  |  |
|  | FGF23 | MYBPC3 | PSMB4 | NRXN1 | PKMYT1 |  |  |  |  |  |
|  | FGF3 | MYH11 | PSMB5 | ORAI1 | POLA1 |  |  |  |  |  |
|  | FGF4 | MYH3 | PSMB6 | PLN | POLA2 |  |  |  |  |  |
|  | FGF5 | MYH6 | PSMB7 | PPP2CA | POLE |  |  |  |  |  |
|  | FGF6 | MYH8 | PSMB8 | PPP2CB | POLE2 |  |  |  |  |  |
|  | FGF7 | MYL1 | PSMB9 | PPP2R1A | PRIM1 |  |  |  |  |  |
|  | FGF8 | MYL10 | PSMC1 | PPP2R1B | PRIM2 |  |  |  |  |  |
|  | FGF9 | MYL12A | PSMC2 | PPP2R3B | RB1 |  |  |  |  |  |
|  | FGFR1 | MYL12B | PSMC3 | PPP2R5A | RPA1 |  |  |  |  |  |
|  | FGFR2 | MYL2 | PSMC4 | PPP2R5B | RPA2 |  |  |  |  |  |
|  | FGFR3 | MYL3 | PSMC5 | PPP2R5C | RPA3 |  |  |  |  |  |
|  | FGFR4 | MYL4 | PSMC6 | PPP2R5D | RPA4 |  |  |  |  |  |
|  | FRS2 | MYL5 | PSMD1 | PPP2R5E | RRM2 |  |  |  |  |  |
|  | FXYD1 | MYL6 | PSMD10 | PRKACA | SCN8A |  |  |  |  |  |
|  | FXYD2 | MYL6B | PSMD11 | PRKACB | TBX5 |  |  |  |  |  |
|  | FXYD3 | MYL7 | PSMD12 | PRKACG | TFDP1 |  |  |  |  |  |
|  | FXYD4 | MYL9 | PSMD13 | RYR1 | TRIB3 |  |  |  |  |  |
|  | FXYD6 | MYLK | PSMD14 | RYR2 | TYMS |  |  |  |  |  |
|  | FXYD7 | MYLPF | PSMD2 | RYR3 | WEE1 |  |  |  |  |  |
|  | FYN | NEB | PSMD3 | SCN1A | WWTR1 |  |  |  |  |  |
|  | GAB1 | NTN4 | PSMD4 | SCN2A |  |  |  |  |  |  |
|  | GAB2 | PXN | PSMD5 | SCN4B |  |  |  |  |  |  |
|  | GP6 | SDC1 | PSMD6 | SCN7A |  |  |  |  |  |  |
|  | GRB2 | SDC4 | PSMD7 | SLC8A2 |  |  |  |  |  |  |
|  | HBEGF | SORBS1 | PSMD8 | SLC8A3 |  |  |  |  |  |  |
|  | HRAS | SORBS3 | PSMD9 | SLN |  |  |  |  |  |  |
|  | IL2 | TCAP | PSME1 | SRI |  |  |  |  |  |  |
|  | IL2RA | TGFB1 | PSME2 | STIM1 |  |  |  |  |  |  |
|  | IL2RB | THBS1 | PSME3 | TRDN |  |  |  |  |  |  |
|  | IL2RG | TLN1 | PSME4 | TRPC1 |  |  |  |  |  |  |
|  | IL3 | TMOD1 | PSMF1 |  |  |  |  |  |  |  |
|  | IL3RA | TNC | PTEN |  |  |  |  |  |  |  |
|  | IL5 | TNNC1 | RELA |  |  |  |  |  |  |  |
|  | IL5RA | TNNC2 | RELB |  |  |  |  |  |  |  |
|  | INSR | TNNI1 | RPS27A |  |  |  |  |  |  |  |
|  | IRS1 | TNNI2 | SCUBE2 |  |  |  |  |  |  |  |
|  | IRS2 | TNNI3 | SEL1L |  |  |  |  |  |  |  |
|  | JAK1 | TNNT1 | SHH |  |  |  |  |  |  |  |
|  | JAK2 | TNNT2 | SKP1 |  |  |  |  |  |  |  |
|  | JAK3 | TNNT3 | SKP2 |  |  |  |  |  |  |  |
|  | KIT | TPM1 | SYVN1 |  |  |  |  |  |  |  |
|  | KITLG | TPM2 | UBA3 |  |  |  |  |  |  |  |
|  | KL | TPM3 | UBA52 |  |  |  |  |  |  |  |
|  | KLB | TPM4 | UBB |  |  |  |  |  |  |  |
|  | KRAS | TRIM72 | UBC |  |  |  |  |  |  |  |
|  | LAT | TTN | UBE2D1 |  |  |  |  |  |  |  |
|  | LCK | TTR | UBE2D2 |  |  |  |  |  |  |  |
|  | LCP2 | VCL | UBE2M |  |  |  |  |  |  |  |
|  | LYN | VIM | VCP |  |  |  |  |  |  |  |
|  | MAP2K1 | VTN |  |  |  |  |  |  |  |  |
|  | MAP2K2 |  |  |  |  |  |  |  |  |  |
|  | MAPK1 |  |  |  |  |  |  |  |  |  |
|  | MAPK3 |  |  |  |  |  |  |  |  |  |
|  | MSN |  |  |  |  |  |  |  |  |  |
|  | NFATC1 |  |  |  |  |  |  |  |  |  |
|  | NFATC2 |  |  |  |  |  |  |  |  |  |
|  | NFATC3 |  |  |  |  |  |  |  |  |  |
|  | NRAS |  |  |  |  |  |  |  |  |  |
|  | NRG1 |  |  |  |  |  |  |  |  |  |
|  | NRG2 |  |  |  |  |  |  |  |  |  |
|  | NRG3 |  |  |  |  |  |  |  |  |  |
|  | NRG4 |  |  |  |  |  |  |  |  |  |
|  | PAK1 |  |  |  |  |  |  |  |  |  |
|  | PAK3 |  |  |  |  |  |  |  |  |  |
|  | PDGFA |  |  |  |  |  |  |  |  |  |
|  | PDGFB |  |  |  |  |  |  |  |  |  |
|  | PDGFRA |  |  |  |  |  |  |  |  |  |
|  | PDGFRB |  |  |  |  |  |  |  |  |  |
|  | PDPK1 |  |  |  |  |  |  |  |  |  |
|  | PDPN |  |  |  |  |  |  |  |  |  |
|  | PHLPP1 |  |  |  |  |  |  |  |  |  |
|  | PHLPP2 |  |  |  |  |  |  |  |  |  |
|  | PIK3CA |  |  |  |  |  |  |  |  |  |
|  | PIK3CB |  |  |  |  |  |  |  |  |  |
|  | PIK3CD |  |  |  |  |  |  |  |  |  |
|  | PIK3CG |  |  |  |  |  |  |  |  |  |
|  | PIK3R1 |  |  |  |  |  |  |  |  |  |
|  | PIK3R2 |  |  |  |  |  |  |  |  |  |
|  | PIK3R3 |  |  |  |  |  |  |  |  |  |
|  | PIK3R5 |  |  |  |  |  |  |  |  |  |
|  | PIK3R6 |  |  |  |  |  |  |  |  |  |
|  | PIP4K2A |  |  |  |  |  |  |  |  |  |
|  | PIP4K2B |  |  |  |  |  |  |  |  |  |
|  | PIP4K2C |  |  |  |  |  |  |  |  |  |
|  | PIP5K1A |  |  |  |  |  |  |  |  |  |
|  | PIP5K1B |  |  |  |  |  |  |  |  |  |
|  | PIP5K1C |  |  |  |  |  |  |  |  |  |
|  | PLCG2 |  |  |  |  |  |  |  |  |  |
|  | PPP3CA |  |  |  |  |  |  |  |  |  |
|  | PPP3CB |  |  |  |  |  |  |  |  |  |
|  | PPP3R1 |  |  |  |  |  |  |  |  |  |
|  | PRKCA |  |  |  |  |  |  |  |  |  |
|  | PRKCD |  |  |  |  |  |  |  |  |  |
|  | PRKCZ |  |  |  |  |  |  |  |  |  |
|  | PTK6 |  |  |  |  |  |  |  |  |  |
|  | PTPN11 |  |  |  |  |  |  |  |  |  |
|  | PTPN6 |  |  |  |  |  |  |  |  |  |
|  | RAC1 |  |  |  |  |  |  |  |  |  |
|  | RAC2 |  |  |  |  |  |  |  |  |  |
|  | RAF1 |  |  |  |  |  |  |  |  |  |
|  | RANBP9 |  |  |  |  |  |  |  |  |  |
|  | RDX |  |  |  |  |  |  |  |  |  |
|  | RHOA |  |  |  |  |  |  |  |  |  |
|  | RHOB |  |  |  |  |  |  |  |  |  |
|  | RHOG |  |  |  |  |  |  |  |  |  |
|  | SDC2 |  |  |  |  |  |  |  |  |  |
|  | SDC3 |  |  |  |  |  |  |  |  |  |
|  | SDCBP |  |  |  |  |  |  |  |  |  |
|  | SHC1 |  |  |  |  |  |  |  |  |  |
|  | SLC8A1 |  |  |  |  |  |  |  |  |  |
|  | SRC |  |  |  |  |  |  |  |  |  |
|  | SYK |  |  |  |  |  |  |  |  |  |
|  | THEM4 |  |  |  |  |  |  |  |  |  |
|  | TRAPPC4 |  |  |  |  |  |  |  |  |  |
|  | TRAT1 |  |  |  |  |  |  |  |  |  |
|  | VAV1 |  |  |  |  |  |  |  |  |  |
|  | VAV2 |  |  |  |  |  |  |  |  |  |
|  | VAV3 |  |  |  |  |  |  |  |  |  |

**Supplementary Table4.** To identify the core genes.

| **Pathway** | **Gene** | **Betweenness** | **Degree** |
| --- | --- | --- | --- |
| Hedgehog ligand biogenesis | UBC | 41594.13907 | 229 |
| Ubiquitin-dependent degradation of Cyclin D1 | UBC | 41594.13907 | 229 |
| Ubiquitin-dependent degradation of Cyclin D | UBC | 41594.13907 | 229 |
| Cyclin E associated events during G1/S transition | UBC | 41594.13907 | 229 |
| Orc1 removal from chromatin | UBC | 41594.13907 | 229 |
| Switching of origins to a post-replicative state | UBC | 41594.13907 | 229 |
| Cyclin A:Cdk2-associated events at S phase entry | UBC | 41594.13907 | 229 |
| CDT1 association with the CDC6:ORC:origin complex | UBC | 41594.13907 | 229 |
| G1/S Transition | UBC | 41594.13907 | 229 |
| CDK-mediated phosphorylation and removal of Cdc6 | UBC | 41594.13907 | 229 |
| Hh mutants abrogate ligand secretion | UBC | 41594.13907 | 229 |
| Removal of licensing factors from origins | UBC | 41594.13907 | 229 |
| Hh mutants that don't undergo autocatalytic processing are degraded by ERAD | UBC | 41594.13907 | 229 |
| Ubiquitin Mediated Degradation of Phosphorylated Cdc25A | UBC | 41594.13907 | 229 |
| p53-Independent DNA Damage Response | UBC | 41594.13907 | 229 |
| p53-Independent G1/S DNA damage checkpoint | UBC | 41594.13907 | 229 |
| C-type lectin receptors (CLRs) | UBC | 41594.13907 | 229 |
| Hedgehog ligand biogenesis | UBB | 9036.253838 | 166 |
| Ubiquitin-dependent degradation of Cyclin D1 | UBB | 9036.253838 | 166 |
| Ubiquitin-dependent degradation of Cyclin D | UBB | 9036.253838 | 166 |
| Cyclin E associated events during G1/S transition | UBB | 9036.253838 | 166 |
| Orc1 removal from chromatin | UBB | 9036.253838 | 166 |
| Switching of origins to a post-replicative state | UBB | 9036.253838 | 166 |
| Cyclin A:Cdk2-associated events at S phase entry | UBB | 9036.253838 | 166 |
| CDT1 association with the CDC6:ORC:origin complex | UBB | 9036.253838 | 166 |
| G1/S Transition | UBB | 9036.253838 | 166 |
| CDK-mediated phosphorylation and removal of Cdc6 | UBB | 9036.253838 | 166 |
| Hh mutants abrogate ligand secretion | UBB | 9036.253838 | 166 |
| Removal of licensing factors from origins | UBB | 9036.253838 | 166 |
| Hh mutants that don't undergo autocatalytic processing are degraded by ERAD | UBB | 9036.253838 | 166 |
| Ubiquitin Mediated Degradation of Phosphorylated Cdc25A | UBB | 9036.253838 | 166 |
| p53-Independent DNA Damage Response | UBB | 9036.253838 | 166 |
| p53-Independent G1/S DNA damage checkpoint | UBB | 9036.253838 | 166 |
| C-type lectin receptors (CLRs) | UBB | 9036.253838 | 166 |
| Hedgehog ligand biogenesis | UBA52 | 7884.714111 | 163 |
| Ubiquitin-dependent degradation of Cyclin D1 | UBA52 | 7884.714111 | 163 |
| Ubiquitin-dependent degradation of Cyclin D | UBA52 | 7884.714111 | 163 |
| Cyclin E associated events during G1/S transition | UBA52 | 7884.714111 | 163 |
| Orc1 removal from chromatin | UBA52 | 7884.714111 | 163 |
| Switching of origins to a post-replicative state | UBA52 | 7884.714111 | 163 |
| Cyclin A:Cdk2-associated events at S phase entry | UBA52 | 7884.714111 | 163 |
| CDT1 association with the CDC6:ORC:origin complex | UBA52 | 7884.714111 | 163 |
| G1/S Transition | UBA52 | 7884.714111 | 163 |
| CDK-mediated phosphorylation and removal of Cdc6 | UBA52 | 7884.714111 | 163 |
| Hh mutants abrogate ligand secretion | UBA52 | 7884.714111 | 163 |
| Removal of licensing factors from origins | UBA52 | 7884.714111 | 163 |
| Hh mutants that don't undergo autocatalytic processing are degraded by ERAD | UBA52 | 7884.714111 | 163 |
| Ubiquitin Mediated Degradation of Phosphorylated Cdc25A | UBA52 | 7884.714111 | 163 |
| p53-Independent DNA Damage Response | UBA52 | 7884.714111 | 163 |
| p53-Independent G1/S DNA damage checkpoint | UBA52 | 7884.714111 | 163 |
| C-type lectin receptors (CLRs) | UBA52 | 7884.714111 | 163 |
| Hedgehog ligand biogenesis | RPS27A | 6068.988543 | 153 |
| Ubiquitin-dependent degradation of Cyclin D1 | RPS27A | 6068.988543 | 153 |
| Ubiquitin-dependent degradation of Cyclin D | RPS27A | 6068.988543 | 153 |
| Cyclin E associated events during G1/S transition | RPS27A | 6068.988543 | 153 |
| Orc1 removal from chromatin | RPS27A | 6068.988543 | 153 |
| Switching of origins to a post-replicative state | RPS27A | 6068.988543 | 153 |
| Cyclin A:Cdk2-associated events at S phase entry | RPS27A | 6068.988543 | 153 |
| CDT1 association with the CDC6:ORC:origin complex | RPS27A | 6068.988543 | 153 |
| G1/S Transition | RPS27A | 6068.988543 | 153 |
| CDK-mediated phosphorylation and removal of Cdc6 | RPS27A | 6068.988543 | 153 |
| Hh mutants abrogate ligand secretion | RPS27A | 6068.988543 | 153 |
| Removal of licensing factors from origins | RPS27A | 6068.988543 | 153 |
| Hh mutants that don't undergo autocatalytic processing are degraded by ERAD | RPS27A | 6068.988543 | 153 |
| Ubiquitin Mediated Degradation of Phosphorylated Cdc25A | RPS27A | 6068.988543 | 153 |
| p53-Independent DNA Damage Response | RPS27A | 6068.988543 | 153 |
| p53-Independent G1/S DNA damage checkpoint | RPS27A | 6068.988543 | 153 |
| C-type lectin receptors (CLRs) | RPS27A | 6068.988543 | 153 |
| GPVI-mediated activation cascade | GRB2 | 5514.867532 | 147 |
| Negative regulation of the PI3K/AKT network | GRB2 | 5514.867532 | 147 |
| Constitutive Signaling by Aberrant PI3K in Cancer | GRB2 | 5514.867532 | 147 |
| PI5P, PP2A and IER3 Regulate PI3K/AKT Signaling | GRB2 | 5514.867532 | 147 |
| L1CAM interactions | MAPK1 | 11697.73492 | 144 |
| Negative regulation of the PI3K/AKT network | MAPK1 | 11697.73492 | 144 |
| PI5P, PP2A and IER3 Regulate PI3K/AKT Signaling | MAPK1 | 11697.73492 | 144 |
| GPVI-mediated activation cascade | PIK3R1 | 4994.059946 | 144 |
| Negative regulation of the PI3K/AKT network | PIK3R1 | 4994.059946 | 144 |
| Constitutive Signaling by Aberrant PI3K in Cancer | PIK3R1 | 4994.059946 | 144 |
| PI5P, PP2A and IER3 Regulate PI3K/AKT Signaling | PIK3R1 | 4994.059946 | 144 |
| GPVI-mediated activation cascade | PIK3CA | 4369.183105 | 142 |
| Negative regulation of the PI3K/AKT network | PIK3CA | 4369.183105 | 142 |
| Constitutive Signaling by Aberrant PI3K in Cancer | PIK3CA | 4369.183105 | 142 |
| PI5P, PP2A and IER3 Regulate PI3K/AKT Signaling | PIK3CA | 4369.183105 | 142 |
| C-type lectin receptors (CLRs) | HRAS | 5457.584496 | 133 |
| L1CAM interactions | MAPK3 | 7615.198171 | 132 |
| Negative regulation of the PI3K/AKT network | MAPK3 | 7615.198171 | 132 |
| PI5P, PP2A and IER3 Regulate PI3K/AKT Signaling | MAPK3 | 7615.198171 | 132 |
| L1CAM interactions | SRC | 10802.17374 | 130 |
| Negative regulation of the PI3K/AKT network | SRC | 10802.17374 | 130 |
| PI5P, PP2A and IER3 Regulate PI3K/AKT Signaling | SRC | 10802.17374 | 130 |
| C-type lectin receptors (CLRs) | SRC | 10802.17374 | 130 |
| L1CAM interactions | EGFR | 7523.471938 | 127 |
| Negative regulation of the PI3K/AKT network | EGFR | 7523.471938 | 127 |
| Constitutive Signaling by Aberrant PI3K in Cancer | EGFR | 7523.471938 | 127 |
| PI5P, PP2A and IER3 Regulate PI3K/AKT Signaling | EGFR | 7523.471938 | 127 |
| C-type lectin receptors (CLRs) | KRAS | 5685.635777 | 126 |
| C-type lectin receptors (CLRs) | NRAS | 4745.749131 | 126 |
| C-type lectin receptors (CLRs) | NFKB1 | 8984.889356 | 125 |
| C-type lectin receptors (CLRs) | TRAF6 | 4368.283547 | 125 |
| C-type lectin receptors (CLRs) | RELA | 4937.229284 | 116 |
| GPVI-mediated activation cascade | PIK3CB | 1790.253316 | 114 |
| Negative regulation of the PI3K/AKT network | PIK3CB | 1790.253316 | 114 |
| Constitutive Signaling by Aberrant PI3K in Cancer | PIK3CB | 1790.253316 | 114 |
| PI5P, PP2A and IER3 Regulate PI3K/AKT Signaling | PIK3CB | 1790.253316 | 114 |
| GPVI-mediated activation cascade | PTPN11 | 2978.865485 | 112 |
| Negative regulation of the PI3K/AKT network | PTPN11 | 2978.865485 | 112 |
| Constitutive Signaling by Aberrant PI3K in Cancer | PTPN11 | 2978.865485 | 112 |
| PI5P, PP2A and IER3 Regulate PI3K/AKT Signaling | PTPN11 | 2978.865485 | 112 |
| GPVI-mediated activation cascade | SHC1 | 1262.539179 | 111 |
| GPVI-mediated activation cascade | JAK2 | 6573.541039 | 109 |
| GPVI-mediated activation cascade | PIK3R2 | 1752.100812 | 109 |
| Negative regulation of the PI3K/AKT network | PIK3R2 | 1752.100812 | 109 |
| Constitutive Signaling by Aberrant PI3K in Cancer | PIK3R2 | 1752.100812 | 109 |
| PI5P, PP2A and IER3 Regulate PI3K/AKT Signaling | PIK3R2 | 1752.100812 | 109 |
| Cyclin E associated events during G1/S transition | CDK2 | 4184.235248 | 106 |
| Orc1 removal from chromatin | CDK2 | 4184.235248 | 106 |
| Switching of origins to a post-replicative state | CDK2 | 4184.235248 | 106 |
| Cyclin A:Cdk2-associated events at S phase entry | CDK2 | 4184.235248 | 106 |
| G1/S Transition | CDK2 | 4184.235248 | 106 |
| CDK-mediated phosphorylation and removal of Cdc6 | CDK2 | 4184.235248 | 106 |
| Removal of licensing factors from origins | CDK2 | 4184.235248 | 106 |
| G1/S Transition | CDK1 | 6163.820776 | 103 |
| E2F mediated regulation of DNA replication | CDK1 | 6163.820776 | 103 |
| Cyclin E associated events during G1/S transition | CDKN1B | 4766.595926 | 101 |
| Orc1 removal from chromatin | CDKN1B | 4766.595926 | 101 |
| Switching of origins to a post-replicative state | CDKN1B | 4766.595926 | 101 |
| Cyclin A:Cdk2-associated events at S phase entry | CDKN1B | 4766.595926 | 101 |
| G1/S Transition | CDKN1B | 4766.595926 | 101 |
| Removal of licensing factors from origins | CDKN1B | 4766.595926 | 101 |
| GPVI-mediated activation cascade | AKT1 | 5438.305862 | 99 |
| Negative regulation of the PI3K/AKT network | AKT1 | 5438.305862 | 99 |
| PI5P, PP2A and IER3 Regulate PI3K/AKT Signaling | AKT1 | 5438.305862 | 99 |
| C-type lectin receptors (CLRs) | PAK2 | 6540.309663 | 97 |
| C-type lectin receptors (CLRs) | NFKBIA | 2826.507612 | 97 |
| GPVI-mediated activation cascade | FYN | 5266.882531 | 94 |
| Negative regulation of the PI3K/AKT network | FYN | 5266.882531 | 94 |
| Constitutive Signaling by Aberrant PI3K in Cancer | FYN | 5266.882531 | 94 |
| PI5P, PP2A and IER3 Regulate PI3K/AKT Signaling | FYN | 5266.882531 | 94 |
| C-type lectin receptors (CLRs) | FYN | 5266.882531 | 94 |
| Cardiac conduction | PRKACA | 12563.78046 | 92 |
| C-type lectin receptors (CLRs) | PRKACA | 12563.78046 | 92 |
| Muscle contraction | PRKACA | 12563.78046 | 92 |
| GPVI-mediated activation cascade | RHOA | 7039.577467 | 90 |
| Orc1 removal from chromatin | CDC6 | 1813.513393 | 90 |
| Switching of origins to a post-replicative state | CDC6 | 1813.513393 | 90 |
| CDT1 association with the CDC6:ORC:origin complex | CDC6 | 1813.513393 | 90 |
| G1/S Transition | CDC6 | 1813.513393 | 90 |
| CDK-mediated phosphorylation and removal of Cdc6 | CDC6 | 1813.513393 | 90 |
| E2F mediated regulation of DNA replication | CDC6 | 1813.513393 | 90 |
| Removal of licensing factors from origins | CDC6 | 1813.513393 | 90 |
| Cyclin E associated events during G1/S transition | CDKN1A | 1780.823547 | 90 |
| Orc1 removal from chromatin | CDKN1A | 1780.823547 | 90 |
| Switching of origins to a post-replicative state | CDKN1A | 1780.823547 | 90 |
| Cyclin A:Cdk2-associated events at S phase entry | CDKN1A | 1780.823547 | 90 |
| G1/S Transition | CDKN1A | 1780.823547 | 90 |
| Removal of licensing factors from origins | CDKN1A | 1780.823547 | 90 |
| GPVI-mediated activation cascade | PIK3CD | 1091.982559 | 90 |
| Negative regulation of the PI3K/AKT network | PIK3CD | 1091.982559 | 90 |
| Constitutive Signaling by Aberrant PI3K in Cancer | PIK3CD | 1091.982559 | 90 |
| PI5P, PP2A and IER3 Regulate PI3K/AKT Signaling | PIK3CD | 1091.982559 | 90 |
| C-type lectin receptors (CLRs) | EP300 | 19184.53494 | 88 |
| GPVI-mediated activation cascade | RAC1 | 4649.551823 | 86 |
| L1CAM interactions | RAC1 | 4649.551823 | 86 |
| Orc1 removal from chromatin | ORC1 | 1488.016215 | 85 |
| Switching of origins to a post-replicative state | ORC1 | 1488.016215 | 85 |
| CDT1 association with the CDC6:ORC:origin complex | ORC1 | 1488.016215 | 85 |
| G1/S Transition | ORC1 | 1488.016215 | 85 |
| E2F mediated regulation of DNA replication | ORC1 | 1488.016215 | 85 |
| Removal of licensing factors from origins | ORC1 | 1488.016215 | 85 |
| Hedgehog ligand biogenesis | PSMC5 | 497.6540773 | 85 |
| Ubiquitin-dependent degradation of Cyclin D1 | PSMC5 | 497.6540773 | 85 |
| Ubiquitin-dependent degradation of Cyclin D | PSMC5 | 497.6540773 | 85 |
| Cross-presentation of soluble exogenous antigens (endosomes) | PSMC5 | 497.6540773 | 85 |
| Cyclin E associated events during G1/S transition | PSMC5 | 497.6540773 | 85 |
| Regulation of ornithine decarboxylase (ODC) | PSMC5 | 497.6540773 | 85 |
| Orc1 removal from chromatin | PSMC5 | 497.6540773 | 85 |
| Switching of origins to a post-replicative state | PSMC5 | 497.6540773 | 85 |
| Cyclin A:Cdk2-associated events at S phase entry | PSMC5 | 497.6540773 | 85 |
| CDT1 association with the CDC6:ORC:origin complex | PSMC5 | 497.6540773 | 85 |
| G1/S Transition | PSMC5 | 497.6540773 | 85 |
| CDK-mediated phosphorylation and removal of Cdc6 | PSMC5 | 497.6540773 | 85 |
| Hh mutants abrogate ligand secretion | PSMC5 | 497.6540773 | 85 |
| Removal of licensing factors from origins | PSMC5 | 497.6540773 | 85 |
| Hh mutants that don't undergo autocatalytic processing are degraded by ERAD | PSMC5 | 497.6540773 | 85 |
| Ubiquitin Mediated Degradation of Phosphorylated Cdc25A | PSMC5 | 497.6540773 | 85 |
| p53-Independent DNA Damage Response | PSMC5 | 497.6540773 | 85 |
| p53-Independent G1/S DNA damage checkpoint | PSMC5 | 497.6540773 | 85 |
| C-type lectin receptors (CLRs) | PSMC5 | 497.6540773 | 85 |
| Hedgehog ligand biogenesis | PSMC2 | 1209.383793 | 84 |
| Ubiquitin-dependent degradation of Cyclin D1 | PSMC2 | 1209.383793 | 84 |
| Ubiquitin-dependent degradation of Cyclin D | PSMC2 | 1209.383793 | 84 |
| Cross-presentation of soluble exogenous antigens (endosomes) | PSMC2 | 1209.383793 | 84 |
| Cyclin E associated events during G1/S transition | PSMC2 | 1209.383793 | 84 |
| Regulation of ornithine decarboxylase (ODC) | PSMC2 | 1209.383793 | 84 |
| Orc1 removal from chromatin | PSMC2 | 1209.383793 | 84 |
| Switching of origins to a post-replicative state | PSMC2 | 1209.383793 | 84 |
| Cyclin A:Cdk2-associated events at S phase entry | PSMC2 | 1209.383793 | 84 |
| CDT1 association with the CDC6:ORC:origin complex | PSMC2 | 1209.383793 | 84 |
| G1/S Transition | PSMC2 | 1209.383793 | 84 |
| CDK-mediated phosphorylation and removal of Cdc6 | PSMC2 | 1209.383793 | 84 |
| Hh mutants abrogate ligand secretion | PSMC2 | 1209.383793 | 84 |
| Removal of licensing factors from origins | PSMC2 | 1209.383793 | 84 |
| Hh mutants that don't undergo autocatalytic processing are degraded by ERAD | PSMC2 | 1209.383793 | 84 |
| Ubiquitin Mediated Degradation of Phosphorylated Cdc25A | PSMC2 | 1209.383793 | 84 |
| p53-Independent DNA Damage Response | PSMC2 | 1209.383793 | 84 |
| p53-Independent G1/S DNA damage checkpoint | PSMC2 | 1209.383793 | 84 |
| C-type lectin receptors (CLRs) | PSMC2 | 1209.383793 | 84 |
| Hedgehog ligand biogenesis | PSMA1 | 655.022451 | 84 |
| Ubiquitin-dependent degradation of Cyclin D1 | PSMA1 | 655.022451 | 84 |
| Ubiquitin-dependent degradation of Cyclin D | PSMA1 | 655.022451 | 84 |
| Cross-presentation of soluble exogenous antigens (endosomes) | PSMA1 | 655.022451 | 84 |
| Cyclin E associated events during G1/S transition | PSMA1 | 655.022451 | 84 |
| Regulation of ornithine decarboxylase (ODC) | PSMA1 | 655.022451 | 84 |
| Orc1 removal from chromatin | PSMA1 | 655.022451 | 84 |
| Switching of origins to a post-replicative state | PSMA1 | 655.022451 | 84 |
| Cyclin A:Cdk2-associated events at S phase entry | PSMA1 | 655.022451 | 84 |
| CDT1 association with the CDC6:ORC:origin complex | PSMA1 | 655.022451 | 84 |
| G1/S Transition | PSMA1 | 655.022451 | 84 |
| CDK-mediated phosphorylation and removal of Cdc6 | PSMA1 | 655.022451 | 84 |
| Hh mutants abrogate ligand secretion | PSMA1 | 655.022451 | 84 |
| Removal of licensing factors from origins | PSMA1 | 655.022451 | 84 |
| Hh mutants that don't undergo autocatalytic processing are degraded by ERAD | PSMA1 | 655.022451 | 84 |
| Ubiquitin Mediated Degradation of Phosphorylated Cdc25A | PSMA1 | 655.022451 | 84 |
| p53-Independent DNA Damage Response | PSMA1 | 655.022451 | 84 |
| p53-Independent G1/S DNA damage checkpoint | PSMA1 | 655.022451 | 84 |
| C-type lectin receptors (CLRs) | PSMA1 | 655.022451 | 84 |
| Hedgehog ligand biogenesis | PSMA7 | 463.6084808 | 84 |
| Ubiquitin-dependent degradation of Cyclin D1 | PSMA7 | 463.6084808 | 84 |
| Ubiquitin-dependent degradation of Cyclin D | PSMA7 | 463.6084808 | 84 |
| Cross-presentation of soluble exogenous antigens (endosomes) | PSMA7 | 463.6084808 | 84 |
| Cyclin E associated events during G1/S transition | PSMA7 | 463.6084808 | 84 |
| Regulation of ornithine decarboxylase (ODC) | PSMA7 | 463.6084808 | 84 |
| Orc1 removal from chromatin | PSMA7 | 463.6084808 | 84 |
| Switching of origins to a post-replicative state | PSMA7 | 463.6084808 | 84 |
| Cyclin A:Cdk2-associated events at S phase entry | PSMA7 | 463.6084808 | 84 |
| CDT1 association with the CDC6:ORC:origin complex | PSMA7 | 463.6084808 | 84 |
| G1/S Transition | PSMA7 | 463.6084808 | 84 |
| CDK-mediated phosphorylation and removal of Cdc6 | PSMA7 | 463.6084808 | 84 |
| Hh mutants abrogate ligand secretion | PSMA7 | 463.6084808 | 84 |
| Removal of licensing factors from origins | PSMA7 | 463.6084808 | 84 |
| Hh mutants that don't undergo autocatalytic processing are degraded by ERAD | PSMA7 | 463.6084808 | 84 |
| Ubiquitin Mediated Degradation of Phosphorylated Cdc25A | PSMA7 | 463.6084808 | 84 |
| p53-Independent DNA Damage Response | PSMA7 | 463.6084808 | 84 |
| p53-Independent G1/S DNA damage checkpoint | PSMA7 | 463.6084808 | 84 |
| C-type lectin receptors (CLRs) | PSMA7 | 463.6084808 | 84 |
| Hedgehog ligand biogenesis | PSMC3 | 361.5732512 | 84 |
| Hedgehog ligand biogenesis | PSMC6 | 361.5732512 | 84 |
| Ubiquitin-dependent degradation of Cyclin D1 | PSMC3 | 361.5732512 | 84 |
| Ubiquitin-dependent degradation of Cyclin D1 | PSMC6 | 361.5732512 | 84 |
| Ubiquitin-dependent degradation of Cyclin D | PSMC3 | 361.5732512 | 84 |
| Ubiquitin-dependent degradation of Cyclin D | PSMC6 | 361.5732512 | 84 |
| Cross-presentation of soluble exogenous antigens (endosomes) | PSMC3 | 361.5732512 | 84 |
| Cross-presentation of soluble exogenous antigens (endosomes) | PSMC6 | 361.5732512 | 84 |
| Cyclin E associated events during G1/S transition | PSMC3 | 361.5732512 | 84 |
| Cyclin E associated events during G1/S transition | PSMC6 | 361.5732512 | 84 |
| Regulation of ornithine decarboxylase (ODC) | PSMC3 | 361.5732512 | 84 |
| Regulation of ornithine decarboxylase (ODC) | PSMC6 | 361.5732512 | 84 |
| Orc1 removal from chromatin | PSMC3 | 361.5732512 | 84 |
| Orc1 removal from chromatin | PSMC6 | 361.5732512 | 84 |
| Switching of origins to a post-replicative state | PSMC3 | 361.5732512 | 84 |
| Switching of origins to a post-replicative state | PSMC6 | 361.5732512 | 84 |
| Cyclin A:Cdk2-associated events at S phase entry | PSMC3 | 361.5732512 | 84 |
| Cyclin A:Cdk2-associated events at S phase entry | PSMC6 | 361.5732512 | 84 |
| CDT1 association with the CDC6:ORC:origin complex | PSMC3 | 361.5732512 | 84 |
| CDT1 association with the CDC6:ORC:origin complex | PSMC6 | 361.5732512 | 84 |
| G1/S Transition | PSMC3 | 361.5732512 | 84 |
| G1/S Transition | PSMC6 | 361.5732512 | 84 |
| CDK-mediated phosphorylation and removal of Cdc6 | PSMC3 | 361.5732512 | 84 |
| CDK-mediated phosphorylation and removal of Cdc6 | PSMC6 | 361.5732512 | 84 |
| Hh mutants abrogate ligand secretion | PSMC3 | 361.5732512 | 84 |
| Hh mutants abrogate ligand secretion | PSMC6 | 361.5732512 | 84 |
| Removal of licensing factors from origins | PSMC3 | 361.5732512 | 84 |
| Removal of licensing factors from origins | PSMC6 | 361.5732512 | 84 |
| Hh mutants that don't undergo autocatalytic processing are degraded by ERAD | PSMC3 | 361.5732512 | 84 |
| Hh mutants that don't undergo autocatalytic processing are degraded by ERAD | PSMC6 | 361.5732512 | 84 |
| Ubiquitin Mediated Degradation of Phosphorylated Cdc25A | PSMC3 | 361.5732512 | 84 |
| Ubiquitin Mediated Degradation of Phosphorylated Cdc25A | PSMC6 | 361.5732512 | 84 |
| p53-Independent DNA Damage Response | PSMC3 | 361.5732512 | 84 |
| p53-Independent DNA Damage Response | PSMC6 | 361.5732512 | 84 |
| p53-Independent G1/S DNA damage checkpoint | PSMC3 | 361.5732512 | 84 |
| p53-Independent G1/S DNA damage checkpoint | PSMC6 | 361.5732512 | 84 |
| C-type lectin receptors (CLRs) | PSMC3 | 361.5732512 | 84 |
| C-type lectin receptors (CLRs) | PSMC6 | 361.5732512 | 84 |
| Hedgehog ligand biogenesis | PSMB1 | 771.6614124 | 83 |
| Ubiquitin-dependent degradation of Cyclin D1 | PSMB1 | 771.6614124 | 83 |
| Ubiquitin-dependent degradation of Cyclin D | PSMB1 | 771.6614124 | 83 |
| Cross-presentation of soluble exogenous antigens (endosomes) | PSMB1 | 771.6614124 | 83 |
| Cyclin E associated events during G1/S transition | PSMB1 | 771.6614124 | 83 |
| Regulation of ornithine decarboxylase (ODC) | PSMB1 | 771.6614124 | 83 |
| Orc1 removal from chromatin | PSMB1 | 771.6614124 | 83 |
| Switching of origins to a post-replicative state | PSMB1 | 771.6614124 | 83 |
| Cyclin A:Cdk2-associated events at S phase entry | PSMB1 | 771.6614124 | 83 |
| CDT1 association with the CDC6:ORC:origin complex | PSMB1 | 771.6614124 | 83 |
| G1/S Transition | PSMB1 | 771.6614124 | 83 |
| CDK-mediated phosphorylation and removal of Cdc6 | PSMB1 | 771.6614124 | 83 |
| Hh mutants abrogate ligand secretion | PSMB1 | 771.6614124 | 83 |
| Removal of licensing factors from origins | PSMB1 | 771.6614124 | 83 |
| Hh mutants that don't undergo autocatalytic processing are degraded by ERAD | PSMB1 | 771.6614124 | 83 |
| Ubiquitin Mediated Degradation of Phosphorylated Cdc25A | PSMB1 | 771.6614124 | 83 |
| p53-Independent DNA Damage Response | PSMB1 | 771.6614124 | 83 |
| p53-Independent G1/S DNA damage checkpoint | PSMB1 | 771.6614124 | 83 |
| C-type lectin receptors (CLRs) | PSMB1 | 771.6614124 | 83 |
| Hedgehog ligand biogenesis | PSMA6 | 694.7096053 | 83 |
| Ubiquitin-dependent degradation of Cyclin D1 | PSMA6 | 694.7096053 | 83 |
| Ubiquitin-dependent degradation of Cyclin D | PSMA6 | 694.7096053 | 83 |
| Cross-presentation of soluble exogenous antigens (endosomes) | PSMA6 | 694.7096053 | 83 |
| Cyclin E associated events during G1/S transition | PSMA6 | 694.7096053 | 83 |
| Regulation of ornithine decarboxylase (ODC) | PSMA6 | 694.7096053 | 83 |
| Orc1 removal from chromatin | PSMA6 | 694.7096053 | 83 |
| Switching of origins to a post-replicative state | PSMA6 | 694.7096053 | 83 |
| Cyclin A:Cdk2-associated events at S phase entry | PSMA6 | 694.7096053 | 83 |
| CDT1 association with the CDC6:ORC:origin complex | PSMA6 | 694.7096053 | 83 |
| G1/S Transition | PSMA6 | 694.7096053 | 83 |
| CDK-mediated phosphorylation and removal of Cdc6 | PSMA6 | 694.7096053 | 83 |
| Hh mutants abrogate ligand secretion | PSMA6 | 694.7096053 | 83 |
| Removal of licensing factors from origins | PSMA6 | 694.7096053 | 83 |
| Hh mutants that don't undergo autocatalytic processing are degraded by ERAD | PSMA6 | 694.7096053 | 83 |
| Ubiquitin Mediated Degradation of Phosphorylated Cdc25A | PSMA6 | 694.7096053 | 83 |
| p53-Independent DNA Damage Response | PSMA6 | 694.7096053 | 83 |
| p53-Independent G1/S DNA damage checkpoint | PSMA6 | 694.7096053 | 83 |
| C-type lectin receptors (CLRs) | PSMA6 | 694.7096053 | 83 |
| Hedgehog ligand biogenesis | PSMB3 | 676.6158054 | 83 |
| Ubiquitin-dependent degradation of Cyclin D1 | PSMB3 | 676.6158054 | 83 |
| Ubiquitin-dependent degradation of Cyclin D | PSMB3 | 676.6158054 | 83 |
| Cross-presentation of soluble exogenous antigens (endosomes) | PSMB3 | 676.6158054 | 83 |
| Cyclin E associated events during G1/S transition | PSMB3 | 676.6158054 | 83 |
| Regulation of ornithine decarboxylase (ODC) | PSMB3 | 676.6158054 | 83 |
| Orc1 removal from chromatin | PSMB3 | 676.6158054 | 83 |
| Switching of origins to a post-replicative state | PSMB3 | 676.6158054 | 83 |
| Cyclin A:Cdk2-associated events at S phase entry | PSMB3 | 676.6158054 | 83 |
| CDT1 association with the CDC6:ORC:origin complex | PSMB3 | 676.6158054 | 83 |
| G1/S Transition | PSMB3 | 676.6158054 | 83 |
| CDK-mediated phosphorylation and removal of Cdc6 | PSMB3 | 676.6158054 | 83 |
| Hh mutants abrogate ligand secretion | PSMB3 | 676.6158054 | 83 |
| Removal of licensing factors from origins | PSMB3 | 676.6158054 | 83 |
| Hh mutants that don't undergo autocatalytic processing are degraded by ERAD | PSMB3 | 676.6158054 | 83 |
| Ubiquitin Mediated Degradation of Phosphorylated Cdc25A | PSMB3 | 676.6158054 | 83 |
| p53-Independent DNA Damage Response | PSMB3 | 676.6158054 | 83 |
| p53-Independent G1/S DNA damage checkpoint | PSMB3 | 676.6158054 | 83 |
| C-type lectin receptors (CLRs) | PSMB3 | 676.6158054 | 83 |
| Hedgehog ligand biogenesis | PSMD4 | 404.5454653 | 83 |
| Ubiquitin-dependent degradation of Cyclin D1 | PSMD4 | 404.5454653 | 83 |
| Ubiquitin-dependent degradation of Cyclin D | PSMD4 | 404.5454653 | 83 |
| Cross-presentation of soluble exogenous antigens (endosomes) | PSMD4 | 404.5454653 | 83 |
| Cyclin E associated events during G1/S transition | PSMD4 | 404.5454653 | 83 |
| Regulation of ornithine decarboxylase (ODC) | PSMD4 | 404.5454653 | 83 |
| Orc1 removal from chromatin | PSMD4 | 404.5454653 | 83 |
| Switching of origins to a post-replicative state | PSMD4 | 404.5454653 | 83 |
| Cyclin A:Cdk2-associated events at S phase entry | PSMD4 | 404.5454653 | 83 |
| CDT1 association with the CDC6:ORC:origin complex | PSMD4 | 404.5454653 | 83 |
| G1/S Transition | PSMD4 | 404.5454653 | 83 |
| CDK-mediated phosphorylation and removal of Cdc6 | PSMD4 | 404.5454653 | 83 |
| Hh mutants abrogate ligand secretion | PSMD4 | 404.5454653 | 83 |
| Removal of licensing factors from origins | PSMD4 | 404.5454653 | 83 |
| Hh mutants that don't undergo autocatalytic processing are degraded by ERAD | PSMD4 | 404.5454653 | 83 |
| Ubiquitin Mediated Degradation of Phosphorylated Cdc25A | PSMD4 | 404.5454653 | 83 |
| p53-Independent DNA Damage Response | PSMD4 | 404.5454653 | 83 |
| p53-Independent G1/S DNA damage checkpoint | PSMD4 | 404.5454653 | 83 |
| C-type lectin receptors (CLRs) | PSMD4 | 404.5454653 | 83 |
| Hedgehog ligand biogenesis | PSMC1 | 376.1207906 | 83 |
| Ubiquitin-dependent degradation of Cyclin D1 | PSMC1 | 376.1207906 | 83 |
| Ubiquitin-dependent degradation of Cyclin D | PSMC1 | 376.1207906 | 83 |
| Cross-presentation of soluble exogenous antigens (endosomes) | PSMC1 | 376.1207906 | 83 |
| Cyclin E associated events during G1/S transition | PSMC1 | 376.1207906 | 83 |
| Regulation of ornithine decarboxylase (ODC) | PSMC1 | 376.1207906 | 83 |
| Orc1 removal from chromatin | PSMC1 | 376.1207906 | 83 |
| Switching of origins to a post-replicative state | PSMC1 | 376.1207906 | 83 |
| Cyclin A:Cdk2-associated events at S phase entry | PSMC1 | 376.1207906 | 83 |
| CDT1 association with the CDC6:ORC:origin complex | PSMC1 | 376.1207906 | 83 |
| G1/S Transition | PSMC1 | 376.1207906 | 83 |
| CDK-mediated phosphorylation and removal of Cdc6 | PSMC1 | 376.1207906 | 83 |
| Hh mutants abrogate ligand secretion | PSMC1 | 376.1207906 | 83 |
| Removal of licensing factors from origins | PSMC1 | 376.1207906 | 83 |
| Hh mutants that don't undergo autocatalytic processing are degraded by ERAD | PSMC1 | 376.1207906 | 83 |
| Ubiquitin Mediated Degradation of Phosphorylated Cdc25A | PSMC1 | 376.1207906 | 83 |
| p53-Independent DNA Damage Response | PSMC1 | 376.1207906 | 83 |
| p53-Independent G1/S DNA damage checkpoint | PSMC1 | 376.1207906 | 83 |
| C-type lectin receptors (CLRs) | PSMC1 | 376.1207906 | 83 |
| Hedgehog ligand biogenesis | PSMA4 | 200.1444949 | 83 |
| Ubiquitin-dependent degradation of Cyclin D1 | PSMA4 | 200.1444949 | 83 |
| Ubiquitin-dependent degradation of Cyclin D | PSMA4 | 200.1444949 | 83 |
| Cross-presentation of soluble exogenous antigens (endosomes) | PSMA4 | 200.1444949 | 83 |
| Cyclin E associated events during G1/S transition | PSMA4 | 200.1444949 | 83 |
| Regulation of ornithine decarboxylase (ODC) | PSMA4 | 200.1444949 | 83 |
| Orc1 removal from chromatin | PSMA4 | 200.1444949 | 83 |
| Switching of origins to a post-replicative state | PSMA4 | 200.1444949 | 83 |
| Cyclin A:Cdk2-associated events at S phase entry | PSMA4 | 200.1444949 | 83 |
| CDT1 association with the CDC6:ORC:origin complex | PSMA4 | 200.1444949 | 83 |
| G1/S Transition | PSMA4 | 200.1444949 | 83 |
| CDK-mediated phosphorylation and removal of Cdc6 | PSMA4 | 200.1444949 | 83 |
| Hh mutants abrogate ligand secretion | PSMA4 | 200.1444949 | 83 |
| Removal of licensing factors from origins | PSMA4 | 200.1444949 | 83 |
| Hh mutants that don't undergo autocatalytic processing are degraded by ERAD | PSMA4 | 200.1444949 | 83 |
| Ubiquitin Mediated Degradation of Phosphorylated Cdc25A | PSMA4 | 200.1444949 | 83 |
| p53-Independent DNA Damage Response | PSMA4 | 200.1444949 | 83 |
| p53-Independent G1/S DNA damage checkpoint | PSMA4 | 200.1444949 | 83 |
| C-type lectin receptors (CLRs) | PSMA4 | 200.1444949 | 83 |
| Negative regulation of the PI3K/AKT network | PTEN | 2423.032327 | 82 |
| Hedgehog ligand biogenesis | PSMC4 | 332.6351755 | 82 |
| Ubiquitin-dependent degradation of Cyclin D1 | PSMC4 | 332.6351755 | 82 |
| Ubiquitin-dependent degradation of Cyclin D | PSMC4 | 332.6351755 | 82 |
| Cross-presentation of soluble exogenous antigens (endosomes) | PSMC4 | 332.6351755 | 82 |
| Cyclin E associated events during G1/S transition | PSMC4 | 332.6351755 | 82 |
| Regulation of ornithine decarboxylase (ODC) | PSMC4 | 332.6351755 | 82 |
| Orc1 removal from chromatin | PSMC4 | 332.6351755 | 82 |
| Switching of origins to a post-replicative state | PSMC4 | 332.6351755 | 82 |
| Cyclin A:Cdk2-associated events at S phase entry | PSMC4 | 332.6351755 | 82 |
| CDT1 association with the CDC6:ORC:origin complex | PSMC4 | 332.6351755 | 82 |
| G1/S Transition | PSMC4 | 332.6351755 | 82 |
| CDK-mediated phosphorylation and removal of Cdc6 | PSMC4 | 332.6351755 | 82 |
| Hh mutants abrogate ligand secretion | PSMC4 | 332.6351755 | 82 |
| Removal of licensing factors from origins | PSMC4 | 332.6351755 | 82 |
| Hh mutants that don't undergo autocatalytic processing are degraded by ERAD | PSMC4 | 332.6351755 | 82 |
| Ubiquitin Mediated Degradation of Phosphorylated Cdc25A | PSMC4 | 332.6351755 | 82 |
| p53-Independent DNA Damage Response | PSMC4 | 332.6351755 | 82 |
| p53-Independent G1/S DNA damage checkpoint | PSMC4 | 332.6351755 | 82 |
| C-type lectin receptors (CLRs) | PSMC4 | 332.6351755 | 82 |
| Hedgehog ligand biogenesis | PSMD2 | 314.8906776 | 82 |
| Ubiquitin-dependent degradation of Cyclin D1 | PSMD2 | 314.8906776 | 82 |
| Ubiquitin-dependent degradation of Cyclin D | PSMD2 | 314.8906776 | 82 |
| Cross-presentation of soluble exogenous antigens (endosomes) | PSMD2 | 314.8906776 | 82 |
| Cyclin E associated events during G1/S transition | PSMD2 | 314.8906776 | 82 |
| Regulation of ornithine decarboxylase (ODC) | PSMD2 | 314.8906776 | 82 |
| Orc1 removal from chromatin | PSMD2 | 314.8906776 | 82 |
| Switching of origins to a post-replicative state | PSMD2 | 314.8906776 | 82 |
| Cyclin A:Cdk2-associated events at S phase entry | PSMD2 | 314.8906776 | 82 |
| CDT1 association with the CDC6:ORC:origin complex | PSMD2 | 314.8906776 | 82 |
| G1/S Transition | PSMD2 | 314.8906776 | 82 |
| CDK-mediated phosphorylation and removal of Cdc6 | PSMD2 | 314.8906776 | 82 |
| Hh mutants abrogate ligand secretion | PSMD2 | 314.8906776 | 82 |
| Removal of licensing factors from origins | PSMD2 | 314.8906776 | 82 |
| Hh mutants that don't undergo autocatalytic processing are degraded by ERAD | PSMD2 | 314.8906776 | 82 |
| Ubiquitin Mediated Degradation of Phosphorylated Cdc25A | PSMD2 | 314.8906776 | 82 |
| p53-Independent DNA Damage Response | PSMD2 | 314.8906776 | 82 |
| p53-Independent G1/S DNA damage checkpoint | PSMD2 | 314.8906776 | 82 |
| C-type lectin receptors (CLRs) | PSMD2 | 314.8906776 | 82 |
| Hedgehog ligand biogenesis | PSMA3 | 199.9181072 | 82 |
| Ubiquitin-dependent degradation of Cyclin D1 | PSMA3 | 199.9181072 | 82 |
| Ubiquitin-dependent degradation of Cyclin D | PSMA3 | 199.9181072 | 82 |
| Cross-presentation of soluble exogenous antigens (endosomes) | PSMA3 | 199.9181072 | 82 |
| Cyclin E associated events during G1/S transition | PSMA3 | 199.9181072 | 82 |
| Regulation of ornithine decarboxylase (ODC) | PSMA3 | 199.9181072 | 82 |
| Orc1 removal from chromatin | PSMA3 | 199.9181072 | 82 |
| Switching of origins to a post-replicative state | PSMA3 | 199.9181072 | 82 |
| Cyclin A:Cdk2-associated events at S phase entry | PSMA3 | 199.9181072 | 82 |
| CDT1 association with the CDC6:ORC:origin complex | PSMA3 | 199.9181072 | 82 |
| G1/S Transition | PSMA3 | 199.9181072 | 82 |
| CDK-mediated phosphorylation and removal of Cdc6 | PSMA3 | 199.9181072 | 82 |
| Hh mutants abrogate ligand secretion | PSMA3 | 199.9181072 | 82 |
| Removal of licensing factors from origins | PSMA3 | 199.9181072 | 82 |
| Hh mutants that don't undergo autocatalytic processing are degraded by ERAD | PSMA3 | 199.9181072 | 82 |
| Ubiquitin Mediated Degradation of Phosphorylated Cdc25A | PSMA3 | 199.9181072 | 82 |
| p53-Independent DNA Damage Response | PSMA3 | 199.9181072 | 82 |
| p53-Independent G1/S DNA damage checkpoint | PSMA3 | 199.9181072 | 82 |
| C-type lectin receptors (CLRs) | PSMA3 | 199.9181072 | 82 |
| C-type lectin receptors (CLRs) | BTRC | 2004.099414 | 81 |
| Ubiquitin-dependent degradation of Cyclin D1 | CCND1 | 1698.672452 | 81 |
| Ubiquitin-dependent degradation of Cyclin D | CCND1 | 1698.672452 | 81 |
| Cyclin E associated events during G1/S transition | CCND1 | 1698.672452 | 81 |
| Cyclin A:Cdk2-associated events at S phase entry | CCND1 | 1698.672452 | 81 |
| G1/S Transition | CCND1 | 1698.672452 | 81 |
| Hedgehog ligand biogenesis | PSMB9 | 748.3233726 | 81 |
| Ubiquitin-dependent degradation of Cyclin D1 | PSMB9 | 748.3233726 | 81 |
| Ubiquitin-dependent degradation of Cyclin D | PSMB9 | 748.3233726 | 81 |
| Cross-presentation of soluble exogenous antigens (endosomes) | PSMB9 | 748.3233726 | 81 |
| Cyclin E associated events during G1/S transition | PSMB9 | 748.3233726 | 81 |
| Regulation of ornithine decarboxylase (ODC) | PSMB9 | 748.3233726 | 81 |
| Orc1 removal from chromatin | PSMB9 | 748.3233726 | 81 |
| Switching of origins to a post-replicative state | PSMB9 | 748.3233726 | 81 |
| Cyclin A:Cdk2-associated events at S phase entry | PSMB9 | 748.3233726 | 81 |
| CDT1 association with the CDC6:ORC:origin complex | PSMB9 | 748.3233726 | 81 |
| G1/S Transition | PSMB9 | 748.3233726 | 81 |
| CDK-mediated phosphorylation and removal of Cdc6 | PSMB9 | 748.3233726 | 81 |
| Hh mutants abrogate ligand secretion | PSMB9 | 748.3233726 | 81 |
| Removal of licensing factors from origins | PSMB9 | 748.3233726 | 81 |
| Hh mutants that don't undergo autocatalytic processing are degraded by ERAD | PSMB9 | 748.3233726 | 81 |
| Ubiquitin Mediated Degradation of Phosphorylated Cdc25A | PSMB9 | 748.3233726 | 81 |
| p53-Independent DNA Damage Response | PSMB9 | 748.3233726 | 81 |
| p53-Independent G1/S DNA damage checkpoint | PSMB9 | 748.3233726 | 81 |
| C-type lectin receptors (CLRs) | PSMB9 | 748.3233726 | 81 |
| Hedgehog ligand biogenesis | PSMD14 | 675.5396999 | 81 |
| Ubiquitin-dependent degradation of Cyclin D1 | PSMD14 | 675.5396999 | 81 |
| Ubiquitin-dependent degradation of Cyclin D | PSMD14 | 675.5396999 | 81 |
| Cross-presentation of soluble exogenous antigens (endosomes) | PSMD14 | 675.5396999 | 81 |
| Cyclin E associated events during G1/S transition | PSMD14 | 675.5396999 | 81 |
| Regulation of ornithine decarboxylase (ODC) | PSMD14 | 675.5396999 | 81 |
| Orc1 removal from chromatin | PSMD14 | 675.5396999 | 81 |
| Switching of origins to a post-replicative state | PSMD14 | 675.5396999 | 81 |
| Cyclin A:Cdk2-associated events at S phase entry | PSMD14 | 675.5396999 | 81 |
| CDT1 association with the CDC6:ORC:origin complex | PSMD14 | 675.5396999 | 81 |
| G1/S Transition | PSMD14 | 675.5396999 | 81 |
| CDK-mediated phosphorylation and removal of Cdc6 | PSMD14 | 675.5396999 | 81 |
| Hh mutants abrogate ligand secretion | PSMD14 | 675.5396999 | 81 |
| Removal of licensing factors from origins | PSMD14 | 675.5396999 | 81 |
| Hh mutants that don't undergo autocatalytic processing are degraded by ERAD | PSMD14 | 675.5396999 | 81 |
| Ubiquitin Mediated Degradation of Phosphorylated Cdc25A | PSMD14 | 675.5396999 | 81 |
| p53-Independent DNA Damage Response | PSMD14 | 675.5396999 | 81 |
| p53-Independent G1/S DNA damage checkpoint | PSMD14 | 675.5396999 | 81 |
| C-type lectin receptors (CLRs) | PSMD14 | 675.5396999 | 81 |
| Hedgehog ligand biogenesis | PSMB4 | 312.3197266 | 81 |
| Ubiquitin-dependent degradation of Cyclin D1 | PSMB4 | 312.3197266 | 81 |
| Ubiquitin-dependent degradation of Cyclin D | PSMB4 | 312.3197266 | 81 |
| Cross-presentation of soluble exogenous antigens (endosomes) | PSMB4 | 312.3197266 | 81 |
| Cyclin E associated events during G1/S transition | PSMB4 | 312.3197266 | 81 |
| Regulation of ornithine decarboxylase (ODC) | PSMB4 | 312.3197266 | 81 |
| Orc1 removal from chromatin | PSMB4 | 312.3197266 | 81 |
| Switching of origins to a post-replicative state | PSMB4 | 312.3197266 | 81 |
| Cyclin A:Cdk2-associated events at S phase entry | PSMB4 | 312.3197266 | 81 |
| CDT1 association with the CDC6:ORC:origin complex | PSMB4 | 312.3197266 | 81 |
| G1/S Transition | PSMB4 | 312.3197266 | 81 |
| CDK-mediated phosphorylation and removal of Cdc6 | PSMB4 | 312.3197266 | 81 |
| Hh mutants abrogate ligand secretion | PSMB4 | 312.3197266 | 81 |
| Removal of licensing factors from origins | PSMB4 | 312.3197266 | 81 |
| Hh mutants that don't undergo autocatalytic processing are degraded by ERAD | PSMB4 | 312.3197266 | 81 |
| Ubiquitin Mediated Degradation of Phosphorylated Cdc25A | PSMB4 | 312.3197266 | 81 |
| p53-Independent DNA Damage Response | PSMB4 | 312.3197266 | 81 |
| p53-Independent G1/S DNA damage checkpoint | PSMB4 | 312.3197266 | 81 |
| C-type lectin receptors (CLRs) | PSMB4 | 312.3197266 | 81 |
| Hedgehog ligand biogenesis | PSMD6 | 221.7062802 | 81 |
| Ubiquitin-dependent degradation of Cyclin D1 | PSMD6 | 221.7062802 | 81 |
| Ubiquitin-dependent degradation of Cyclin D | PSMD6 | 221.7062802 | 81 |
| Cross-presentation of soluble exogenous antigens (endosomes) | PSMD6 | 221.7062802 | 81 |
| Cyclin E associated events during G1/S transition | PSMD6 | 221.7062802 | 81 |
| Regulation of ornithine decarboxylase (ODC) | PSMD6 | 221.7062802 | 81 |
| Orc1 removal from chromatin | PSMD6 | 221.7062802 | 81 |
| Switching of origins to a post-replicative state | PSMD6 | 221.7062802 | 81 |
| Cyclin A:Cdk2-associated events at S phase entry | PSMD6 | 221.7062802 | 81 |
| CDT1 association with the CDC6:ORC:origin complex | PSMD6 | 221.7062802 | 81 |
| G1/S Transition | PSMD6 | 221.7062802 | 81 |
| CDK-mediated phosphorylation and removal of Cdc6 | PSMD6 | 221.7062802 | 81 |
| Hh mutants abrogate ligand secretion | PSMD6 | 221.7062802 | 81 |
| Removal of licensing factors from origins | PSMD6 | 221.7062802 | 81 |
| Hh mutants that don't undergo autocatalytic processing are degraded by ERAD | PSMD6 | 221.7062802 | 81 |
| Ubiquitin Mediated Degradation of Phosphorylated Cdc25A | PSMD6 | 221.7062802 | 81 |
| p53-Independent DNA Damage Response | PSMD6 | 221.7062802 | 81 |
| p53-Independent G1/S DNA damage checkpoint | PSMD6 | 221.7062802 | 81 |
| C-type lectin receptors (CLRs) | PSMD6 | 221.7062802 | 81 |
| Hedgehog ligand biogenesis | PSMB10 | 180.2773897 | 81 |
| Ubiquitin-dependent degradation of Cyclin D1 | PSMB10 | 180.2773897 | 81 |
| Ubiquitin-dependent degradation of Cyclin D | PSMB10 | 180.2773897 | 81 |
| Cross-presentation of soluble exogenous antigens (endosomes) | PSMB10 | 180.2773897 | 81 |
| Cyclin E associated events during G1/S transition | PSMB10 | 180.2773897 | 81 |
| Regulation of ornithine decarboxylase (ODC) | PSMB10 | 180.2773897 | 81 |
| Orc1 removal from chromatin | PSMB10 | 180.2773897 | 81 |
| Switching of origins to a post-replicative state | PSMB10 | 180.2773897 | 81 |
| Cyclin A:Cdk2-associated events at S phase entry | PSMB10 | 180.2773897 | 81 |
| CDT1 association with the CDC6:ORC:origin complex | PSMB10 | 180.2773897 | 81 |
| G1/S Transition | PSMB10 | 180.2773897 | 81 |
| CDK-mediated phosphorylation and removal of Cdc6 | PSMB10 | 180.2773897 | 81 |
| Hh mutants abrogate ligand secretion | PSMB10 | 180.2773897 | 81 |
| Removal of licensing factors from origins | PSMB10 | 180.2773897 | 81 |
| Hh mutants that don't undergo autocatalytic processing are degraded by ERAD | PSMB10 | 180.2773897 | 81 |
| Ubiquitin Mediated Degradation of Phosphorylated Cdc25A | PSMB10 | 180.2773897 | 81 |
| p53-Independent DNA Damage Response | PSMB10 | 180.2773897 | 81 |
| p53-Independent G1/S DNA damage checkpoint | PSMB10 | 180.2773897 | 81 |
| C-type lectin receptors (CLRs) | PSMB10 | 180.2773897 | 81 |
| Hedgehog ligand biogenesis | PSMA5 | 170.2285154 | 81 |
| Ubiquitin-dependent degradation of Cyclin D1 | PSMA5 | 170.2285154 | 81 |
| Ubiquitin-dependent degradation of Cyclin D | PSMA5 | 170.2285154 | 81 |
| Cross-presentation of soluble exogenous antigens (endosomes) | PSMA5 | 170.2285154 | 81 |
| Cyclin E associated events during G1/S transition | PSMA5 | 170.2285154 | 81 |
| Regulation of ornithine decarboxylase (ODC) | PSMA5 | 170.2285154 | 81 |
| Orc1 removal from chromatin | PSMA5 | 170.2285154 | 81 |
| Switching of origins to a post-replicative state | PSMA5 | 170.2285154 | 81 |
| Cyclin A:Cdk2-associated events at S phase entry | PSMA5 | 170.2285154 | 81 |
| CDT1 association with the CDC6:ORC:origin complex | PSMA5 | 170.2285154 | 81 |
| G1/S Transition | PSMA5 | 170.2285154 | 81 |
| CDK-mediated phosphorylation and removal of Cdc6 | PSMA5 | 170.2285154 | 81 |
| Hh mutants abrogate ligand secretion | PSMA5 | 170.2285154 | 81 |
| Removal of licensing factors from origins | PSMA5 | 170.2285154 | 81 |
| Hh mutants that don't undergo autocatalytic processing are degraded by ERAD | PSMA5 | 170.2285154 | 81 |
| Ubiquitin Mediated Degradation of Phosphorylated Cdc25A | PSMA5 | 170.2285154 | 81 |
| p53-Independent DNA Damage Response | PSMA5 | 170.2285154 | 81 |
| p53-Independent G1/S DNA damage checkpoint | PSMA5 | 170.2285154 | 81 |
| C-type lectin receptors (CLRs) | PSMA5 | 170.2285154 | 81 |
| Hedgehog ligand biogenesis | PSME1 | 693.6211816 | 80 |
| Ubiquitin-dependent degradation of Cyclin D1 | PSME1 | 693.6211816 | 80 |
| Ubiquitin-dependent degradation of Cyclin D | PSME1 | 693.6211816 | 80 |
| Cross-presentation of soluble exogenous antigens (endosomes) | PSME1 | 693.6211816 | 80 |
| Cyclin E associated events during G1/S transition | PSME1 | 693.6211816 | 80 |
| Regulation of ornithine decarboxylase (ODC) | PSME1 | 693.6211816 | 80 |
| Orc1 removal from chromatin | PSME1 | 693.6211816 | 80 |
| Switching of origins to a post-replicative state | PSME1 | 693.6211816 | 80 |
| Cyclin A:Cdk2-associated events at S phase entry | PSME1 | 693.6211816 | 80 |
| CDT1 association with the CDC6:ORC:origin complex | PSME1 | 693.6211816 | 80 |
| G1/S Transition | PSME1 | 693.6211816 | 80 |
| CDK-mediated phosphorylation and removal of Cdc6 | PSME1 | 693.6211816 | 80 |
| Hh mutants abrogate ligand secretion | PSME1 | 693.6211816 | 80 |
| Removal of licensing factors from origins | PSME1 | 693.6211816 | 80 |
| Hh mutants that don't undergo autocatalytic processing are degraded by ERAD | PSME1 | 693.6211816 | 80 |
| Ubiquitin Mediated Degradation of Phosphorylated Cdc25A | PSME1 | 693.6211816 | 80 |
| p53-Independent DNA Damage Response | PSME1 | 693.6211816 | 80 |
| p53-Independent G1/S DNA damage checkpoint | PSME1 | 693.6211816 | 80 |
| C-type lectin receptors (CLRs) | PSME1 | 693.6211816 | 80 |
| Hedgehog ligand biogenesis | PSMD7 | 563.5725209 | 80 |
| Ubiquitin-dependent degradation of Cyclin D1 | PSMD7 | 563.5725209 | 80 |
| Ubiquitin-dependent degradation of Cyclin D | PSMD7 | 563.5725209 | 80 |
| Cross-presentation of soluble exogenous antigens (endosomes) | PSMD7 | 563.5725209 | 80 |
| Cyclin E associated events during G1/S transition | PSMD7 | 563.5725209 | 80 |
| Regulation of ornithine decarboxylase (ODC) | PSMD7 | 563.5725209 | 80 |
| Orc1 removal from chromatin | PSMD7 | 563.5725209 | 80 |
| Switching of origins to a post-replicative state | PSMD7 | 563.5725209 | 80 |
| Cyclin A:Cdk2-associated events at S phase entry | PSMD7 | 563.5725209 | 80 |
| CDT1 association with the CDC6:ORC:origin complex | PSMD7 | 563.5725209 | 80 |
| G1/S Transition | PSMD7 | 563.5725209 | 80 |
| CDK-mediated phosphorylation and removal of Cdc6 | PSMD7 | 563.5725209 | 80 |
| Hh mutants abrogate ligand secretion | PSMD7 | 563.5725209 | 80 |
| Removal of licensing factors from origins | PSMD7 | 563.5725209 | 80 |
| Hh mutants that don't undergo autocatalytic processing are degraded by ERAD | PSMD7 | 563.5725209 | 80 |
| Ubiquitin Mediated Degradation of Phosphorylated Cdc25A | PSMD7 | 563.5725209 | 80 |
| p53-Independent DNA Damage Response | PSMD7 | 563.5725209 | 80 |
| p53-Independent G1/S DNA damage checkpoint | PSMD7 | 563.5725209 | 80 |
| C-type lectin receptors (CLRs) | PSMD7 | 563.5725209 | 80 |
| Hedgehog ligand biogenesis | PSMD1 | 295.9396359 | 80 |
| Ubiquitin-dependent degradation of Cyclin D1 | PSMD1 | 295.9396359 | 80 |
| Ubiquitin-dependent degradation of Cyclin D | PSMD1 | 295.9396359 | 80 |
| Cross-presentation of soluble exogenous antigens (endosomes) | PSMD1 | 295.9396359 | 80 |
| Cyclin E associated events during G1/S transition | PSMD1 | 295.9396359 | 80 |
| Regulation of ornithine decarboxylase (ODC) | PSMD1 | 295.9396359 | 80 |
| Orc1 removal from chromatin | PSMD1 | 295.9396359 | 80 |
| Switching of origins to a post-replicative state | PSMD1 | 295.9396359 | 80 |
| Cyclin A:Cdk2-associated events at S phase entry | PSMD1 | 295.9396359 | 80 |
| CDT1 association with the CDC6:ORC:origin complex | PSMD1 | 295.9396359 | 80 |
| G1/S Transition | PSMD1 | 295.9396359 | 80 |
| CDK-mediated phosphorylation and removal of Cdc6 | PSMD1 | 295.9396359 | 80 |
| Hh mutants abrogate ligand secretion | PSMD1 | 295.9396359 | 80 |
| Removal of licensing factors from origins | PSMD1 | 295.9396359 | 80 |
| Hh mutants that don't undergo autocatalytic processing are degraded by ERAD | PSMD1 | 295.9396359 | 80 |
| Ubiquitin Mediated Degradation of Phosphorylated Cdc25A | PSMD1 | 295.9396359 | 80 |
| p53-Independent DNA Damage Response | PSMD1 | 295.9396359 | 80 |
| p53-Independent G1/S DNA damage checkpoint | PSMD1 | 295.9396359 | 80 |
| C-type lectin receptors (CLRs) | PSMD1 | 295.9396359 | 80 |
| Hedgehog ligand biogenesis | PSMD9 | 173.767773 | 80 |
| Ubiquitin-dependent degradation of Cyclin D1 | PSMD9 | 173.767773 | 80 |
| Ubiquitin-dependent degradation of Cyclin D | PSMD9 | 173.767773 | 80 |
| Cross-presentation of soluble exogenous antigens (endosomes) | PSMD9 | 173.767773 | 80 |
| Cyclin E associated events during G1/S transition | PSMD9 | 173.767773 | 80 |
| Regulation of ornithine decarboxylase (ODC) | PSMD9 | 173.767773 | 80 |
| Orc1 removal from chromatin | PSMD9 | 173.767773 | 80 |
| Switching of origins to a post-replicative state | PSMD9 | 173.767773 | 80 |
| Cyclin A:Cdk2-associated events at S phase entry | PSMD9 | 173.767773 | 80 |
| CDT1 association with the CDC6:ORC:origin complex | PSMD9 | 173.767773 | 80 |
| G1/S Transition | PSMD9 | 173.767773 | 80 |
| CDK-mediated phosphorylation and removal of Cdc6 | PSMD9 | 173.767773 | 80 |
| Hh mutants abrogate ligand secretion | PSMD9 | 173.767773 | 80 |
| Removal of licensing factors from origins | PSMD9 | 173.767773 | 80 |
| Hh mutants that don't undergo autocatalytic processing are degraded by ERAD | PSMD9 | 173.767773 | 80 |
| Ubiquitin Mediated Degradation of Phosphorylated Cdc25A | PSMD9 | 173.767773 | 80 |
| p53-Independent DNA Damage Response | PSMD9 | 173.767773 | 80 |
| p53-Independent G1/S DNA damage checkpoint | PSMD9 | 173.767773 | 80 |
| C-type lectin receptors (CLRs) | PSMD9 | 173.767773 | 80 |
| Hedgehog ligand biogenesis | PSMD13 | 164.130392 | 80 |
| Ubiquitin-dependent degradation of Cyclin D1 | PSMD13 | 164.130392 | 80 |
| Ubiquitin-dependent degradation of Cyclin D | PSMD13 | 164.130392 | 80 |
| Cross-presentation of soluble exogenous antigens (endosomes) | PSMD13 | 164.130392 | 80 |
| Cyclin E associated events during G1/S transition | PSMD13 | 164.130392 | 80 |
| Regulation of ornithine decarboxylase (ODC) | PSMD13 | 164.130392 | 80 |
| Orc1 removal from chromatin | PSMD13 | 164.130392 | 80 |
| Switching of origins to a post-replicative state | PSMD13 | 164.130392 | 80 |
| Cyclin A:Cdk2-associated events at S phase entry | PSMD13 | 164.130392 | 80 |
| CDT1 association with the CDC6:ORC:origin complex | PSMD13 | 164.130392 | 80 |
| G1/S Transition | PSMD13 | 164.130392 | 80 |
| CDK-mediated phosphorylation and removal of Cdc6 | PSMD13 | 164.130392 | 80 |
| Hh mutants abrogate ligand secretion | PSMD13 | 164.130392 | 80 |
| Removal of licensing factors from origins | PSMD13 | 164.130392 | 80 |
| Hh mutants that don't undergo autocatalytic processing are degraded by ERAD | PSMD13 | 164.130392 | 80 |
| Ubiquitin Mediated Degradation of Phosphorylated Cdc25A | PSMD13 | 164.130392 | 80 |
| p53-Independent DNA Damage Response | PSMD13 | 164.130392 | 80 |
| p53-Independent G1/S DNA damage checkpoint | PSMD13 | 164.130392 | 80 |
| C-type lectin receptors (CLRs) | PSMD13 | 164.130392 | 80 |
| Hedgehog ligand biogenesis | PSMA2 | 159.3847845 | 80 |
| Hedgehog ligand biogenesis | PSMB7 | 159.3847845 | 80 |
| Ubiquitin-dependent degradation of Cyclin D1 | PSMA2 | 159.3847845 | 80 |
| Ubiquitin-dependent degradation of Cyclin D1 | PSMB7 | 159.3847845 | 80 |
| Ubiquitin-dependent degradation of Cyclin D | PSMA2 | 159.3847845 | 80 |
| Ubiquitin-dependent degradation of Cyclin D | PSMB7 | 159.3847845 | 80 |
| Cross-presentation of soluble exogenous antigens (endosomes) | PSMA2 | 159.3847845 | 80 |
| Cross-presentation of soluble exogenous antigens (endosomes) | PSMB7 | 159.3847845 | 80 |
| Cyclin E associated events during G1/S transition | PSMA2 | 159.3847845 | 80 |
| Cyclin E associated events during G1/S transition | PSMB7 | 159.3847845 | 80 |
| Regulation of ornithine decarboxylase (ODC) | PSMA2 | 159.3847845 | 80 |
| Regulation of ornithine decarboxylase (ODC) | PSMB7 | 159.3847845 | 80 |
| Orc1 removal from chromatin | PSMA2 | 159.3847845 | 80 |
| Orc1 removal from chromatin | PSMB7 | 159.3847845 | 80 |
| Switching of origins to a post-replicative state | PSMA2 | 159.3847845 | 80 |
| Switching of origins to a post-replicative state | PSMB7 | 159.3847845 | 80 |
| Cyclin A:Cdk2-associated events at S phase entry | PSMA2 | 159.3847845 | 80 |
| Cyclin A:Cdk2-associated events at S phase entry | PSMB7 | 159.3847845 | 80 |
| CDT1 association with the CDC6:ORC:origin complex | PSMA2 | 159.3847845 | 80 |
| CDT1 association with the CDC6:ORC:origin complex | PSMB7 | 159.3847845 | 80 |
| G1/S Transition | PSMA2 | 159.3847845 | 80 |
| G1/S Transition | PSMB7 | 159.3847845 | 80 |
| CDK-mediated phosphorylation and removal of Cdc6 | PSMA2 | 159.3847845 | 80 |
| CDK-mediated phosphorylation and removal of Cdc6 | PSMB7 | 159.3847845 | 80 |
| Hh mutants abrogate ligand secretion | PSMA2 | 159.3847845 | 80 |
| Hh mutants abrogate ligand secretion | PSMB7 | 159.3847845 | 80 |
| Removal of licensing factors from origins | PSMA2 | 159.3847845 | 80 |
| Removal of licensing factors from origins | PSMB7 | 159.3847845 | 80 |
| Hh mutants that don't undergo autocatalytic processing are degraded by ERAD | PSMA2 | 159.3847845 | 80 |
| Hh mutants that don't undergo autocatalytic processing are degraded by ERAD | PSMB7 | 159.3847845 | 80 |
| Ubiquitin Mediated Degradation of Phosphorylated Cdc25A | PSMA2 | 159.3847845 | 80 |
| Ubiquitin Mediated Degradation of Phosphorylated Cdc25A | PSMB7 | 159.3847845 | 80 |
| p53-Independent DNA Damage Response | PSMA2 | 159.3847845 | 80 |
| p53-Independent DNA Damage Response | PSMB7 | 159.3847845 | 80 |
| p53-Independent G1/S DNA damage checkpoint | PSMA2 | 159.3847845 | 80 |
| p53-Independent G1/S DNA damage checkpoint | PSMB7 | 159.3847845 | 80 |
| C-type lectin receptors (CLRs) | PSMA2 | 159.3847845 | 80 |
| C-type lectin receptors (CLRs) | PSMB7 | 159.3847845 | 80 |
| Hedgehog ligand biogenesis | PSMD3 | 155.3662807 | 80 |
| Ubiquitin-dependent degradation of Cyclin D1 | PSMD3 | 155.3662807 | 80 |
| Ubiquitin-dependent degradation of Cyclin D | PSMD3 | 155.3662807 | 80 |
| Cross-presentation of soluble exogenous antigens (endosomes) | PSMD3 | 155.3662807 | 80 |
| Cyclin E associated events during G1/S transition | PSMD3 | 155.3662807 | 80 |
| Regulation of ornithine decarboxylase (ODC) | PSMD3 | 155.3662807 | 80 |
| Orc1 removal from chromatin | PSMD3 | 155.3662807 | 80 |
| Switching of origins to a post-replicative state | PSMD3 | 155.3662807 | 80 |
| Cyclin A:Cdk2-associated events at S phase entry | PSMD3 | 155.3662807 | 80 |
| CDT1 association with the CDC6:ORC:origin complex | PSMD3 | 155.3662807 | 80 |
| G1/S Transition | PSMD3 | 155.3662807 | 80 |
| CDK-mediated phosphorylation and removal of Cdc6 | PSMD3 | 155.3662807 | 80 |
| Hh mutants abrogate ligand secretion | PSMD3 | 155.3662807 | 80 |
| Removal of licensing factors from origins | PSMD3 | 155.3662807 | 80 |
| Hh mutants that don't undergo autocatalytic processing are degraded by ERAD | PSMD3 | 155.3662807 | 80 |
| Ubiquitin Mediated Degradation of Phosphorylated Cdc25A | PSMD3 | 155.3662807 | 80 |
| p53-Independent DNA Damage Response | PSMD3 | 155.3662807 | 80 |
| p53-Independent G1/S DNA damage checkpoint | PSMD3 | 155.3662807 | 80 |
| C-type lectin receptors (CLRs) | PSMD3 | 155.3662807 | 80 |
| Orc1 removal from chromatin | FZR1 | 690.9823201 | 79 |
| Switching of origins to a post-replicative state | FZR1 | 690.9823201 | 79 |
| Cyclin A:Cdk2-associated events at S phase entry | FZR1 | 690.9823201 | 79 |
| Removal of licensing factors from origins | FZR1 | 690.9823201 | 79 |
| Cyclin E associated events during G1/S transition | SKP1 | 653.9655416 | 79 |
| Cyclin A:Cdk2-associated events at S phase entry | SKP1 | 653.9655416 | 79 |
| G1/S Transition | SKP1 | 653.9655416 | 79 |
| C-type lectin receptors (CLRs) | SKP1 | 653.9655416 | 79 |
| Hedgehog ligand biogenesis | PSME2 | 278.5723636 | 79 |
| Ubiquitin-dependent degradation of Cyclin D1 | PSME2 | 278.5723636 | 79 |
| Ubiquitin-dependent degradation of Cyclin D | PSME2 | 278.5723636 | 79 |
| Cross-presentation of soluble exogenous antigens (endosomes) | PSME2 | 278.5723636 | 79 |
| Cyclin E associated events during G1/S transition | PSME2 | 278.5723636 | 79 |
| Regulation of ornithine decarboxylase (ODC) | PSME2 | 278.5723636 | 79 |
| Orc1 removal from chromatin | PSME2 | 278.5723636 | 79 |
| Switching of origins to a post-replicative state | PSME2 | 278.5723636 | 79 |
| Cyclin A:Cdk2-associated events at S phase entry | PSME2 | 278.5723636 | 79 |
| CDT1 association with the CDC6:ORC:origin complex | PSME2 | 278.5723636 | 79 |
| G1/S Transition | PSME2 | 278.5723636 | 79 |
| CDK-mediated phosphorylation and removal of Cdc6 | PSME2 | 278.5723636 | 79 |
| Hh mutants abrogate ligand secretion | PSME2 | 278.5723636 | 79 |
| Removal of licensing factors from origins | PSME2 | 278.5723636 | 79 |
| Hh mutants that don't undergo autocatalytic processing are degraded by ERAD | PSME2 | 278.5723636 | 79 |
| Ubiquitin Mediated Degradation of Phosphorylated Cdc25A | PSME2 | 278.5723636 | 79 |
| p53-Independent DNA Damage Response | PSME2 | 278.5723636 | 79 |
| p53-Independent G1/S DNA damage checkpoint | PSME2 | 278.5723636 | 79 |
| C-type lectin receptors (CLRs) | PSME2 | 278.5723636 | 79 |
| Hedgehog ligand biogenesis | PSMB2 | 145.2497728 | 79 |
| Hedgehog ligand biogenesis | PSMB5 | 145.2497728 | 79 |
| Hedgehog ligand biogenesis | PSMB6 | 145.2497728 | 79 |
| Hedgehog ligand biogenesis | PSMD11 | 145.2497728 | 79 |
| Hedgehog ligand biogenesis | PSMD12 | 145.2497728 | 79 |
| Hedgehog ligand biogenesis | PSMD8 | 145.2497728 | 79 |
| Ubiquitin-dependent degradation of Cyclin D1 | PSMB2 | 145.2497728 | 79 |
| Ubiquitin-dependent degradation of Cyclin D1 | PSMB5 | 145.2497728 | 79 |
| Ubiquitin-dependent degradation of Cyclin D1 | PSMB6 | 145.2497728 | 79 |
| Ubiquitin-dependent degradation of Cyclin D1 | PSMD11 | 145.2497728 | 79 |
| Ubiquitin-dependent degradation of Cyclin D1 | PSMD12 | 145.2497728 | 79 |
| Ubiquitin-dependent degradation of Cyclin D1 | PSMD8 | 145.2497728 | 79 |
| Ubiquitin-dependent degradation of Cyclin D | PSMB2 | 145.2497728 | 79 |
| Ubiquitin-dependent degradation of Cyclin D | PSMB5 | 145.2497728 | 79 |
| Ubiquitin-dependent degradation of Cyclin D | PSMB6 | 145.2497728 | 79 |
| Ubiquitin-dependent degradation of Cyclin D | PSMD11 | 145.2497728 | 79 |
| Ubiquitin-dependent degradation of Cyclin D | PSMD12 | 145.2497728 | 79 |
| Ubiquitin-dependent degradation of Cyclin D | PSMD8 | 145.2497728 | 79 |
| Cross-presentation of soluble exogenous antigens (endosomes) | PSMB2 | 145.2497728 | 79 |
| Cross-presentation of soluble exogenous antigens (endosomes) | PSMB5 | 145.2497728 | 79 |
| Cross-presentation of soluble exogenous antigens (endosomes) | PSMB6 | 145.2497728 | 79 |
| Cross-presentation of soluble exogenous antigens (endosomes) | PSMD11 | 145.2497728 | 79 |
| Cross-presentation of soluble exogenous antigens (endosomes) | PSMD12 | 145.2497728 | 79 |
| Cross-presentation of soluble exogenous antigens (endosomes) | PSMD8 | 145.2497728 | 79 |
| Cyclin E associated events during G1/S transition | PSMB2 | 145.2497728 | 79 |
| Cyclin E associated events during G1/S transition | PSMB5 | 145.2497728 | 79 |
| Cyclin E associated events during G1/S transition | PSMB6 | 145.2497728 | 79 |
| Cyclin E associated events during G1/S transition | PSMD11 | 145.2497728 | 79 |
| Cyclin E associated events during G1/S transition | PSMD12 | 145.2497728 | 79 |
| Cyclin E associated events during G1/S transition | PSMD8 | 145.2497728 | 79 |
| Regulation of ornithine decarboxylase (ODC) | PSMB2 | 145.2497728 | 79 |
| Regulation of ornithine decarboxylase (ODC) | PSMB5 | 145.2497728 | 79 |
| Regulation of ornithine decarboxylase (ODC) | PSMB6 | 145.2497728 | 79 |
| Regulation of ornithine decarboxylase (ODC) | PSMD11 | 145.2497728 | 79 |
| Regulation of ornithine decarboxylase (ODC) | PSMD12 | 145.2497728 | 79 |
| Regulation of ornithine decarboxylase (ODC) | PSMD8 | 145.2497728 | 79 |
| Orc1 removal from chromatin | PSMB2 | 145.2497728 | 79 |
| Orc1 removal from chromatin | PSMB5 | 145.2497728 | 79 |
| Orc1 removal from chromatin | PSMB6 | 145.2497728 | 79 |
| Orc1 removal from chromatin | PSMD11 | 145.2497728 | 79 |
| Orc1 removal from chromatin | PSMD12 | 145.2497728 | 79 |
| Orc1 removal from chromatin | PSMD8 | 145.2497728 | 79 |
| Switching of origins to a post-replicative state | PSMB2 | 145.2497728 | 79 |
| Switching of origins to a post-replicative state | PSMB5 | 145.2497728 | 79 |
| Switching of origins to a post-replicative state | PSMB6 | 145.2497728 | 79 |
| Switching of origins to a post-replicative state | PSMD11 | 145.2497728 | 79 |
| Switching of origins to a post-replicative state | PSMD12 | 145.2497728 | 79 |
| Switching of origins to a post-replicative state | PSMD8 | 145.2497728 | 79 |
| Cyclin A:Cdk2-associated events at S phase entry | PSMB2 | 145.2497728 | 79 |
| Cyclin A:Cdk2-associated events at S phase entry | PSMB5 | 145.2497728 | 79 |
| Cyclin A:Cdk2-associated events at S phase entry | PSMB6 | 145.2497728 | 79 |
| Cyclin A:Cdk2-associated events at S phase entry | PSMD11 | 145.2497728 | 79 |
| Cyclin A:Cdk2-associated events at S phase entry | PSMD12 | 145.2497728 | 79 |
| Cyclin A:Cdk2-associated events at S phase entry | PSMD8 | 145.2497728 | 79 |
| CDT1 association with the CDC6:ORC:origin complex | PSMB2 | 145.2497728 | 79 |
| CDT1 association with the CDC6:ORC:origin complex | PSMB5 | 145.2497728 | 79 |
| CDT1 association with the CDC6:ORC:origin complex | PSMB6 | 145.2497728 | 79 |
| CDT1 association with the CDC6:ORC:origin complex | PSMD11 | 145.2497728 | 79 |
| CDT1 association with the CDC6:ORC:origin complex | PSMD12 | 145.2497728 | 79 |
| CDT1 association with the CDC6:ORC:origin complex | PSMD8 | 145.2497728 | 79 |
| G1/S Transition | PSMB2 | 145.2497728 | 79 |
| G1/S Transition | PSMB5 | 145.2497728 | 79 |
| G1/S Transition | PSMB6 | 145.2497728 | 79 |
| G1/S Transition | PSMD11 | 145.2497728 | 79 |
| G1/S Transition | PSMD12 | 145.2497728 | 79 |
| G1/S Transition | PSMD8 | 145.2497728 | 79 |
| CDK-mediated phosphorylation and removal of Cdc6 | PSMB2 | 145.2497728 | 79 |
| CDK-mediated phosphorylation and removal of Cdc6 | PSMB5 | 145.2497728 | 79 |
| CDK-mediated phosphorylation and removal of Cdc6 | PSMB6 | 145.2497728 | 79 |
| CDK-mediated phosphorylation and removal of Cdc6 | PSMD11 | 145.2497728 | 79 |
| CDK-mediated phosphorylation and removal of Cdc6 | PSMD12 | 145.2497728 | 79 |
| CDK-mediated phosphorylation and removal of Cdc6 | PSMD8 | 145.2497728 | 79 |
| Hh mutants abrogate ligand secretion | PSMB2 | 145.2497728 | 79 |
| Hh mutants abrogate ligand secretion | PSMB5 | 145.2497728 | 79 |
| Hh mutants abrogate ligand secretion | PSMB6 | 145.2497728 | 79 |
| Hh mutants abrogate ligand secretion | PSMD11 | 145.2497728 | 79 |
| Hh mutants abrogate ligand secretion | PSMD12 | 145.2497728 | 79 |
| Hh mutants abrogate ligand secretion | PSMD8 | 145.2497728 | 79 |
| Removal of licensing factors from origins | PSMB2 | 145.2497728 | 79 |
| Removal of licensing factors from origins | PSMB5 | 145.2497728 | 79 |
| Removal of licensing factors from origins | PSMB6 | 145.2497728 | 79 |
| Removal of licensing factors from origins | PSMD11 | 145.2497728 | 79 |
| Removal of licensing factors from origins | PSMD12 | 145.2497728 | 79 |
| Removal of licensing factors from origins | PSMD8 | 145.2497728 | 79 |
| Hh mutants that don't undergo autocatalytic processing are degraded by ERAD | PSMB2 | 145.2497728 | 79 |
| Hh mutants that don't undergo autocatalytic processing are degraded by ERAD | PSMB5 | 145.2497728 | 79 |
| Hh mutants that don't undergo autocatalytic processing are degraded by ERAD | PSMB6 | 145.2497728 | 79 |
| Hh mutants that don't undergo autocatalytic processing are degraded by ERAD | PSMD11 | 145.2497728 | 79 |
| Hh mutants that don't undergo autocatalytic processing are degraded by ERAD | PSMD12 | 145.2497728 | 79 |
| Hh mutants that don't undergo autocatalytic processing are degraded by ERAD | PSMD8 | 145.2497728 | 79 |
| Ubiquitin Mediated Degradation of Phosphorylated Cdc25A | PSMB2 | 145.2497728 | 79 |
| Ubiquitin Mediated Degradation of Phosphorylated Cdc25A | PSMB5 | 145.2497728 | 79 |
| Ubiquitin Mediated Degradation of Phosphorylated Cdc25A | PSMB6 | 145.2497728 | 79 |
| Ubiquitin Mediated Degradation of Phosphorylated Cdc25A | PSMD11 | 145.2497728 | 79 |
| Ubiquitin Mediated Degradation of Phosphorylated Cdc25A | PSMD12 | 145.2497728 | 79 |
| Ubiquitin Mediated Degradation of Phosphorylated Cdc25A | PSMD8 | 145.2497728 | 79 |
| p53-Independent DNA Damage Response | PSMB2 | 145.2497728 | 79 |
| p53-Independent DNA Damage Response | PSMB5 | 145.2497728 | 79 |
| p53-Independent DNA Damage Response | PSMB6 | 145.2497728 | 79 |
| p53-Independent DNA Damage Response | PSMD11 | 145.2497728 | 79 |
| p53-Independent DNA Damage Response | PSMD12 | 145.2497728 | 79 |
| p53-Independent DNA Damage Response | PSMD8 | 145.2497728 | 79 |
| p53-Independent G1/S DNA damage checkpoint | PSMB2 | 145.2497728 | 79 |
| p53-Independent G1/S DNA damage checkpoint | PSMB5 | 145.2497728 | 79 |
| p53-Independent G1/S DNA damage checkpoint | PSMB6 | 145.2497728 | 79 |
| p53-Independent G1/S DNA damage checkpoint | PSMD11 | 145.2497728 | 79 |
| p53-Independent G1/S DNA damage checkpoint | PSMD12 | 145.2497728 | 79 |
| p53-Independent G1/S DNA damage checkpoint | PSMD8 | 145.2497728 | 79 |
| C-type lectin receptors (CLRs) | PSMB2 | 145.2497728 | 79 |
| C-type lectin receptors (CLRs) | PSMB5 | 145.2497728 | 79 |
| C-type lectin receptors (CLRs) | PSMB6 | 145.2497728 | 79 |
| C-type lectin receptors (CLRs) | PSMD11 | 145.2497728 | 79 |
| C-type lectin receptors (CLRs) | PSMD12 | 145.2497728 | 79 |
| C-type lectin receptors (CLRs) | PSMD8 | 145.2497728 | 79 |
| Hedgehog ligand biogenesis | PSME3 | 138.6335492 | 79 |
| Ubiquitin-dependent degradation of Cyclin D1 | PSME3 | 138.6335492 | 79 |
| Ubiquitin-dependent degradation of Cyclin D | PSME3 | 138.6335492 | 79 |
| Cross-presentation of soluble exogenous antigens (endosomes) | PSME3 | 138.6335492 | 79 |
| Cyclin E associated events during G1/S transition | PSME3 | 138.6335492 | 79 |
| Regulation of ornithine decarboxylase (ODC) | PSME3 | 138.6335492 | 79 |
| Orc1 removal from chromatin | PSME3 | 138.6335492 | 79 |
| Switching of origins to a post-replicative state | PSME3 | 138.6335492 | 79 |
| Cyclin A:Cdk2-associated events at S phase entry | PSME3 | 138.6335492 | 79 |
| CDT1 association with the CDC6:ORC:origin complex | PSME3 | 138.6335492 | 79 |
| G1/S Transition | PSME3 | 138.6335492 | 79 |
| CDK-mediated phosphorylation and removal of Cdc6 | PSME3 | 138.6335492 | 79 |
| Hh mutants abrogate ligand secretion | PSME3 | 138.6335492 | 79 |
| Removal of licensing factors from origins | PSME3 | 138.6335492 | 79 |
| Hh mutants that don't undergo autocatalytic processing are degraded by ERAD | PSME3 | 138.6335492 | 79 |
| Ubiquitin Mediated Degradation of Phosphorylated Cdc25A | PSME3 | 138.6335492 | 79 |
| p53-Independent DNA Damage Response | PSME3 | 138.6335492 | 79 |
| p53-Independent G1/S DNA damage checkpoint | PSME3 | 138.6335492 | 79 |
| C-type lectin receptors (CLRs) | PSME3 | 138.6335492 | 79 |
| GPVI-mediated activation cascade | PLCG2 | 4877.846624 | 78 |
| C-type lectin receptors (CLRs) | PLCG2 | 4877.846624 | 78 |
| Hedgehog ligand biogenesis | PSMB8 | 128.6341891 | 78 |
| Hedgehog ligand biogenesis | PSMD10 | 128.6341891 | 78 |
| Hedgehog ligand biogenesis | PSMD5 | 128.6341891 | 78 |
| Hedgehog ligand biogenesis | PSMF1 | 128.6341891 | 78 |
| Ubiquitin-dependent degradation of Cyclin D1 | PSMB8 | 128.6341891 | 78 |
| Ubiquitin-dependent degradation of Cyclin D1 | PSMD10 | 128.6341891 | 78 |
| Ubiquitin-dependent degradation of Cyclin D1 | PSMD5 | 128.6341891 | 78 |
| Ubiquitin-dependent degradation of Cyclin D1 | PSMF1 | 128.6341891 | 78 |
| Ubiquitin-dependent degradation of Cyclin D | PSMB8 | 128.6341891 | 78 |
| Ubiquitin-dependent degradation of Cyclin D | PSMD10 | 128.6341891 | 78 |
| Ubiquitin-dependent degradation of Cyclin D | PSMD5 | 128.6341891 | 78 |
| Ubiquitin-dependent degradation of Cyclin D | PSMF1 | 128.6341891 | 78 |
| Cross-presentation of soluble exogenous antigens (endosomes) | PSMB8 | 128.6341891 | 78 |
| Cross-presentation of soluble exogenous antigens (endosomes) | PSMD10 | 128.6341891 | 78 |
| Cross-presentation of soluble exogenous antigens (endosomes) | PSMD5 | 128.6341891 | 78 |
| Cross-presentation of soluble exogenous antigens (endosomes) | PSMF1 | 128.6341891 | 78 |
| Cyclin E associated events during G1/S transition | PSMB8 | 128.6341891 | 78 |
| Cyclin E associated events during G1/S transition | PSMD10 | 128.6341891 | 78 |
| Cyclin E associated events during G1/S transition | PSMD5 | 128.6341891 | 78 |
| Cyclin E associated events during G1/S transition | PSMF1 | 128.6341891 | 78 |
| Regulation of ornithine decarboxylase (ODC) | PSMB8 | 128.6341891 | 78 |
| Regulation of ornithine decarboxylase (ODC) | PSMD10 | 128.6341891 | 78 |
| Regulation of ornithine decarboxylase (ODC) | PSMD5 | 128.6341891 | 78 |
| Regulation of ornithine decarboxylase (ODC) | PSMF1 | 128.6341891 | 78 |
| Orc1 removal from chromatin | PSMB8 | 128.6341891 | 78 |
| Orc1 removal from chromatin | PSMD10 | 128.6341891 | 78 |
| Orc1 removal from chromatin | PSMD5 | 128.6341891 | 78 |
| Orc1 removal from chromatin | PSMF1 | 128.6341891 | 78 |
| Switching of origins to a post-replicative state | PSMB8 | 128.6341891 | 78 |
| Switching of origins to a post-replicative state | PSMD10 | 128.6341891 | 78 |
| Switching of origins to a post-replicative state | PSMD5 | 128.6341891 | 78 |
| Switching of origins to a post-replicative state | PSMF1 | 128.6341891 | 78 |
| Cyclin A:Cdk2-associated events at S phase entry | PSMB8 | 128.6341891 | 78 |
| Cyclin A:Cdk2-associated events at S phase entry | PSMD10 | 128.6341891 | 78 |
| Cyclin A:Cdk2-associated events at S phase entry | PSMD5 | 128.6341891 | 78 |
| Cyclin A:Cdk2-associated events at S phase entry | PSMF1 | 128.6341891 | 78 |
| CDT1 association with the CDC6:ORC:origin complex | PSMB8 | 128.6341891 | 78 |
| CDT1 association with the CDC6:ORC:origin complex | PSMD10 | 128.6341891 | 78 |
| CDT1 association with the CDC6:ORC:origin complex | PSMD5 | 128.6341891 | 78 |
| CDT1 association with the CDC6:ORC:origin complex | PSMF1 | 128.6341891 | 78 |
| G1/S Transition | PSMB8 | 128.6341891 | 78 |
| G1/S Transition | PSMD10 | 128.6341891 | 78 |
| G1/S Transition | PSMD5 | 128.6341891 | 78 |
| G1/S Transition | PSMF1 | 128.6341891 | 78 |
| CDK-mediated phosphorylation and removal of Cdc6 | PSMB8 | 128.6341891 | 78 |
| CDK-mediated phosphorylation and removal of Cdc6 | PSMD10 | 128.6341891 | 78 |
| CDK-mediated phosphorylation and removal of Cdc6 | PSMD5 | 128.6341891 | 78 |
| CDK-mediated phosphorylation and removal of Cdc6 | PSMF1 | 128.6341891 | 78 |
| Hh mutants abrogate ligand secretion | PSMB8 | 128.6341891 | 78 |
| Hh mutants abrogate ligand secretion | PSMD10 | 128.6341891 | 78 |
| Hh mutants abrogate ligand secretion | PSMD5 | 128.6341891 | 78 |
| Hh mutants abrogate ligand secretion | PSMF1 | 128.6341891 | 78 |
| Removal of licensing factors from origins | PSMB8 | 128.6341891 | 78 |
| Removal of licensing factors from origins | PSMD10 | 128.6341891 | 78 |
| Removal of licensing factors from origins | PSMD5 | 128.6341891 | 78 |
| Removal of licensing factors from origins | PSMF1 | 128.6341891 | 78 |
| Hh mutants that don't undergo autocatalytic processing are degraded by ERAD | PSMB8 | 128.6341891 | 78 |
| Hh mutants that don't undergo autocatalytic processing are degraded by ERAD | PSMD10 | 128.6341891 | 78 |
| Hh mutants that don't undergo autocatalytic processing are degraded by ERAD | PSMD5 | 128.6341891 | 78 |
| Hh mutants that don't undergo autocatalytic processing are degraded by ERAD | PSMF1 | 128.6341891 | 78 |
| Ubiquitin Mediated Degradation of Phosphorylated Cdc25A | PSMB8 | 128.6341891 | 78 |
| Ubiquitin Mediated Degradation of Phosphorylated Cdc25A | PSMD10 | 128.6341891 | 78 |
| Ubiquitin Mediated Degradation of Phosphorylated Cdc25A | PSMD5 | 128.6341891 | 78 |
| Ubiquitin Mediated Degradation of Phosphorylated Cdc25A | PSMF1 | 128.6341891 | 78 |
| p53-Independent DNA Damage Response | PSMB8 | 128.6341891 | 78 |
| p53-Independent DNA Damage Response | PSMD10 | 128.6341891 | 78 |
| p53-Independent DNA Damage Response | PSMD5 | 128.6341891 | 78 |
| p53-Independent DNA Damage Response | PSMF1 | 128.6341891 | 78 |
| p53-Independent G1/S DNA damage checkpoint | PSMB8 | 128.6341891 | 78 |
| p53-Independent G1/S DNA damage checkpoint | PSMD10 | 128.6341891 | 78 |
| p53-Independent G1/S DNA damage checkpoint | PSMD5 | 128.6341891 | 78 |
| p53-Independent G1/S DNA damage checkpoint | PSMF1 | 128.6341891 | 78 |
| C-type lectin receptors (CLRs) | PSMB8 | 128.6341891 | 78 |
| C-type lectin receptors (CLRs) | PSMD10 | 128.6341891 | 78 |
| C-type lectin receptors (CLRs) | PSMD5 | 128.6341891 | 78 |
| C-type lectin receptors (CLRs) | PSMF1 | 128.6341891 | 78 |
| L1CAM interactions | ITGB1 | 6112.415682 | 77 |
| Non-integrin membrane-ECM interactions | ITGB1 | 6112.415682 | 77 |
| GPVI-mediated activation cascade | PIK3CG | 982.0969521 | 77 |
| GPVI-mediated activation cascade | PIK3R3 | 747.2700494 | 77 |
| Negative regulation of the PI3K/AKT network | PIK3R3 | 747.2700494 | 77 |
| Constitutive Signaling by Aberrant PI3K in Cancer | PIK3R3 | 747.2700494 | 77 |
| PI5P, PP2A and IER3 Regulate PI3K/AKT Signaling | PIK3R3 | 747.2700494 | 77 |
| Hedgehog ligand biogenesis | PSME4 | 268.1127336 | 76 |
| Ubiquitin-dependent degradation of Cyclin D1 | PSME4 | 268.1127336 | 76 |
| Ubiquitin-dependent degradation of Cyclin D | PSME4 | 268.1127336 | 76 |
| Cross-presentation of soluble exogenous antigens (endosomes) | PSME4 | 268.1127336 | 76 |
| Regulation of ornithine decarboxylase (ODC) | PSME4 | 268.1127336 | 76 |
| Orc1 removal from chromatin | PSME4 | 268.1127336 | 76 |
| Switching of origins to a post-replicative state | PSME4 | 268.1127336 | 76 |
| CDT1 association with the CDC6:ORC:origin complex | PSME4 | 268.1127336 | 76 |
| CDK-mediated phosphorylation and removal of Cdc6 | PSME4 | 268.1127336 | 76 |
| Hh mutants abrogate ligand secretion | PSME4 | 268.1127336 | 76 |
| Removal of licensing factors from origins | PSME4 | 268.1127336 | 76 |
| Hh mutants that don't undergo autocatalytic processing are degraded by ERAD | PSME4 | 268.1127336 | 76 |
| Ubiquitin Mediated Degradation of Phosphorylated Cdc25A | PSME4 | 268.1127336 | 76 |
| p53-Independent DNA Damage Response | PSME4 | 268.1127336 | 76 |
| p53-Independent G1/S DNA damage checkpoint | PSME4 | 268.1127336 | 76 |
| C-type lectin receptors (CLRs) | PSME4 | 268.1127336 | 76 |
| Hedgehog ligand biogenesis | PSMA8 | 141.8679026 | 76 |
| Ubiquitin-dependent degradation of Cyclin D1 | PSMA8 | 141.8679026 | 76 |
| Ubiquitin-dependent degradation of Cyclin D | PSMA8 | 141.8679026 | 76 |
| Cross-presentation of soluble exogenous antigens (endosomes) | PSMA8 | 141.8679026 | 76 |
| Regulation of ornithine decarboxylase (ODC) | PSMA8 | 141.8679026 | 76 |
| Orc1 removal from chromatin | PSMA8 | 141.8679026 | 76 |
| Switching of origins to a post-replicative state | PSMA8 | 141.8679026 | 76 |
| CDT1 association with the CDC6:ORC:origin complex | PSMA8 | 141.8679026 | 76 |
| CDK-mediated phosphorylation and removal of Cdc6 | PSMA8 | 141.8679026 | 76 |
| Hh mutants abrogate ligand secretion | PSMA8 | 141.8679026 | 76 |
| Removal of licensing factors from origins | PSMA8 | 141.8679026 | 76 |
| Hh mutants that don't undergo autocatalytic processing are degraded by ERAD | PSMA8 | 141.8679026 | 76 |
| Ubiquitin Mediated Degradation of Phosphorylated Cdc25A | PSMA8 | 141.8679026 | 76 |
| p53-Independent DNA Damage Response | PSMA8 | 141.8679026 | 76 |
| p53-Independent G1/S DNA damage checkpoint | PSMA8 | 141.8679026 | 76 |
| C-type lectin receptors (CLRs) | PSMA8 | 141.8679026 | 76 |
| C-type lectin receptors (CLRs) | PRKACB | 6849.447923 | 75 |
| C-type lectin receptors (CLRs) | MAP3K14 | 765.5663224 | 75 |
| Cyclin E associated events during G1/S transition | CUL1 | 296.5444519 | 75 |
| Cyclin A:Cdk2-associated events at S phase entry | CUL1 | 296.5444519 | 75 |
| G1/S Transition | CUL1 | 296.5444519 | 75 |
| C-type lectin receptors (CLRs) | CUL1 | 296.5444519 | 75 |
| C-type lectin receptors (CLRs) | PRKACG | 7879.67283 | 74 |
| Non-integrin membrane-ECM interactions | PRKCA | 2923.451083 | 74 |
| Hedgehog ligand biogenesis | PSMB11 | 111.3225703 | 74 |
| Ubiquitin-dependent degradation of Cyclin D1 | PSMB11 | 111.3225703 | 74 |
| Ubiquitin-dependent degradation of Cyclin D | PSMB11 | 111.3225703 | 74 |
| Cross-presentation of soluble exogenous antigens (endosomes) | PSMB11 | 111.3225703 | 74 |
| Regulation of ornithine decarboxylase (ODC) | PSMB11 | 111.3225703 | 74 |
| Orc1 removal from chromatin | PSMB11 | 111.3225703 | 74 |
| Switching of origins to a post-replicative state | PSMB11 | 111.3225703 | 74 |
| CDT1 association with the CDC6:ORC:origin complex | PSMB11 | 111.3225703 | 74 |
| CDK-mediated phosphorylation and removal of Cdc6 | PSMB11 | 111.3225703 | 74 |
| Hh mutants abrogate ligand secretion | PSMB11 | 111.3225703 | 74 |
| Removal of licensing factors from origins | PSMB11 | 111.3225703 | 74 |
| Hh mutants that don't undergo autocatalytic processing are degraded by ERAD | PSMB11 | 111.3225703 | 74 |
| Ubiquitin Mediated Degradation of Phosphorylated Cdc25A | PSMB11 | 111.3225703 | 74 |
| p53-Independent DNA Damage Response | PSMB11 | 111.3225703 | 74 |
| p53-Independent G1/S DNA damage checkpoint | PSMB11 | 111.3225703 | 74 |
| C-type lectin receptors (CLRs) | PSMB11 | 111.3225703 | 74 |
| Negative regulation of the PI3K/AKT network | GAB1 | 410.5114802 | 73 |
| Constitutive Signaling by Aberrant PI3K in Cancer | GAB1 | 410.5114802 | 73 |
| PI5P, PP2A and IER3 Regulate PI3K/AKT Signaling | GAB1 | 410.5114802 | 73 |
| GPVI-mediated activation cascade | PIK3R5 | 644.527249 | 71 |
| C-type lectin receptors (CLRs) | UBE2D1 | 329.8361879 | 71 |
| Negative regulation of the PI3K/AKT network | EGF | 1231.710449 | 70 |
| Constitutive Signaling by Aberrant PI3K in Cancer | EGF | 1231.710449 | 70 |
| PI5P, PP2A and IER3 Regulate PI3K/AKT Signaling | EGF | 1231.710449 | 70 |
| Negative regulation of the PI3K/AKT network | PDGFRB | 754.3095067 | 70 |
| Constitutive Signaling by Aberrant PI3K in Cancer | PDGFRB | 754.3095067 | 70 |
| PI5P, PP2A and IER3 Regulate PI3K/AKT Signaling | PDGFRB | 754.3095067 | 70 |
| GPVI-mediated activation cascade | LCK | 407.8925645 | 69 |
| Negative regulation of the PI3K/AKT network | LCK | 407.8925645 | 69 |
| Constitutive Signaling by Aberrant PI3K in Cancer | LCK | 407.8925645 | 69 |
| PI5P, PP2A and IER3 Regulate PI3K/AKT Signaling | LCK | 407.8925645 | 69 |
| L1CAM interactions | ITGB3 | 2498.866984 | 68 |
| Non-integrin membrane-ECM interactions | ITGB3 | 2498.866984 | 68 |
| L1CAM interactions | FGFR1 | 990.0107716 | 67 |
| Negative regulation of the PI3K/AKT network | FGFR1 | 990.0107716 | 67 |
| Constitutive Signaling by Aberrant PI3K in Cancer | FGFR1 | 990.0107716 | 67 |
| PI5P, PP2A and IER3 Regulate PI3K/AKT Signaling | FGFR1 | 990.0107716 | 67 |
| GPVI-mediated activation cascade | GAB2 | 421.7680059 | 67 |
| C-type lectin receptors (CLRs) | NFKB2 | 176.319644 | 67 |
| GPVI-mediated activation cascade | LYN | 2340.098986 | 66 |
| C-type lectin receptors (CLRs) | LYN | 2340.098986 | 66 |
| Cyclin E associated events during G1/S transition | SKP2 | 204.2102244 | 66 |
| Cyclin A:Cdk2-associated events at S phase entry | SKP2 | 204.2102244 | 66 |
| G1/S Transition | SKP2 | 204.2102244 | 66 |
| Hedgehog ligand biogenesis | SHH | 8575.544205 | 65 |
| Hh mutants abrogate ligand secretion | SHH | 8575.544205 | 65 |
| Hh mutants that don't undergo autocatalytic processing are degraded by ERAD | SHH | 8575.544205 | 65 |
| Negative regulation of the PI3K/AKT network | PPP2CA | 3216.763729 | 65 |
| G1/S Transition | PPP2CA | 3216.763729 | 65 |
| E2F mediated regulation of DNA replication | PPP2CA | 3216.763729 | 65 |
| PI5P, PP2A and IER3 Regulate PI3K/AKT Signaling | PPP2CA | 3216.763729 | 65 |
| GPVI-mediated activation cascade | PRKCZ | 1023.435862 | 65 |
| Negative regulation of the PI3K/AKT network | FGFR3 | 471.5744863 | 65 |
| Constitutive Signaling by Aberrant PI3K in Cancer | FGFR3 | 471.5744863 | 65 |
| PI5P, PP2A and IER3 Regulate PI3K/AKT Signaling | FGFR3 | 471.5744863 | 65 |
| C-type lectin receptors (CLRs) | RELB | 135.612679 | 65 |
| Negative regulation of the PI3K/AKT network | FGFR2 | 556.6051848 | 64 |
| Constitutive Signaling by Aberrant PI3K in Cancer | FGFR2 | 556.6051848 | 64 |
| PI5P, PP2A and IER3 Regulate PI3K/AKT Signaling | FGFR2 | 556.6051848 | 64 |
| Muscle contraction | PXN | 3331.555469 | 63 |
| GPVI-mediated activation cascade | PDPK1 | 1437.879243 | 63 |
| C-type lectin receptors (CLRs) | PDPK1 | 1437.879243 | 63 |
| Cyclin E associated events during G1/S transition | MYC | 2537.243448 | 62 |
| Cyclin A:Cdk2-associated events at S phase entry | MYC | 2537.243448 | 62 |
| G1/S Transition | MYC | 2537.243448 | 62 |
| C-type lectin receptors (CLRs) | RAF1 | 872.63808 | 62 |
| Muscle contraction | ACTN2 | 7997.007888 | 61 |
| Negative regulation of the PI3K/AKT network | ERBB2 | 688.9042126 | 61 |
| Constitutive Signaling by Aberrant PI3K in Cancer | ERBB2 | 688.9042126 | 61 |
| PI5P, PP2A and IER3 Regulate PI3K/AKT Signaling | ERBB2 | 688.9042126 | 61 |
| Negative regulation of the PI3K/AKT network | FGFR4 | 390.2806844 | 61 |
| Constitutive Signaling by Aberrant PI3K in Cancer | FGFR4 | 390.2806844 | 61 |
| PI5P, PP2A and IER3 Regulate PI3K/AKT Signaling | FGFR4 | 390.2806844 | 61 |
| Negative regulation of the PI3K/AKT network | PPP2CB | 2949.169694 | 60 |
| G1/S Transition | PPP2CB | 2949.169694 | 60 |
| E2F mediated regulation of DNA replication | PPP2CB | 2949.169694 | 60 |
| PI5P, PP2A and IER3 Regulate PI3K/AKT Signaling | PPP2CB | 2949.169694 | 60 |
| Negative regulation of the PI3K/AKT network | PDGFRA | 687.501783 | 60 |
| Constitutive Signaling by Aberrant PI3K in Cancer | PDGFRA | 687.501783 | 60 |
| PI5P, PP2A and IER3 Regulate PI3K/AKT Signaling | PDGFRA | 687.501783 | 60 |
| Negative regulation of the PI3K/AKT network | PPP2R1A | 2704.659917 | 59 |
| G1/S Transition | PPP2R1A | 2704.659917 | 59 |
| E2F mediated regulation of DNA replication | PPP2R1A | 2704.659917 | 59 |
| PI5P, PP2A and IER3 Regulate PI3K/AKT Signaling | PPP2R1A | 2704.659917 | 59 |
| Negative regulation of the PI3K/AKT network | FGF2 | 1242.164037 | 59 |
| Constitutive Signaling by Aberrant PI3K in Cancer | FGF2 | 1242.164037 | 59 |
| PI5P, PP2A and IER3 Regulate PI3K/AKT Signaling | FGF2 | 1242.164037 | 59 |
| Non-integrin membrane-ECM interactions | FGF2 | 1242.164037 | 59 |
| Cyclin E associated events during G1/S transition | CKS1B | 41.79810805 | 59 |
| Cyclin A:Cdk2-associated events at S phase entry | CKS1B | 41.79810805 | 59 |
| G1/S Transition | CKS1B | 41.79810805 | 59 |
| Cardiac conduction | CAMK2A | 3769.973902 | 58 |
| Phase 0 - rapid depolarisation | CAMK2A | 3769.973902 | 58 |
| Muscle contraction | CAMK2A | 3769.973902 | 58 |
| Ubiquitin-dependent degradation of Cyclin D1 | CDK4 | 2580.428717 | 58 |
| Ubiquitin-dependent degradation of Cyclin D | CDK4 | 2580.428717 | 58 |
| Cyclin E associated events during G1/S transition | CDK4 | 2580.428717 | 58 |
| Cyclin A:Cdk2-associated events at S phase entry | CDK4 | 2580.428717 | 58 |
| G1/S Transition | CDK4 | 2580.428717 | 58 |
| L1CAM interactions | ITGAV | 2146.800612 | 58 |
| Non-integrin membrane-ECM interactions | ITGAV | 2146.800612 | 58 |
| GPVI-mediated activation cascade | SYK | 1411.564785 | 58 |
| C-type lectin receptors (CLRs) | SYK | 1411.564785 | 58 |
| GPVI-mediated activation cascade | JAK1 | 367.6351811 | 58 |
| Cardiac conduction | CAMK2B | 3405.539396 | 57 |
| Cardiac conduction | CAMK2D | 3405.539396 | 57 |
| Cardiac conduction | CAMK2G | 3405.539396 | 57 |
| Phase 0 - rapid depolarisation | CAMK2B | 3405.539396 | 57 |
| Phase 0 - rapid depolarisation | CAMK2D | 3405.539396 | 57 |
| Phase 0 - rapid depolarisation | CAMK2G | 3405.539396 | 57 |
| Muscle contraction | CAMK2B | 3405.539396 | 57 |
| Muscle contraction | CAMK2D | 3405.539396 | 57 |
| Muscle contraction | CAMK2G | 3405.539396 | 57 |
| GPVI-mediated activation cascade | AKT2 | 1514.765756 | 57 |
| Negative regulation of the PI3K/AKT network | AKT2 | 1514.765756 | 57 |
| Negative regulation of the PI3K/AKT network | KIT | 461.9421181 | 57 |
| Constitutive Signaling by Aberrant PI3K in Cancer | KIT | 461.9421181 | 57 |
| PI5P, PP2A and IER3 Regulate PI3K/AKT Signaling | KIT | 461.9421181 | 57 |
| Cyclin E associated events during G1/S transition | CDC25A | 95.31190108 | 57 |
| Cyclin A:Cdk2-associated events at S phase entry | CDC25A | 95.31190108 | 57 |
| G1/S Transition | CDC25A | 95.31190108 | 57 |
| E2F mediated regulation of DNA replication | CDC25A | 95.31190108 | 57 |
| Ubiquitin Mediated Degradation of Phosphorylated Cdc25A | CDC25A | 95.31190108 | 57 |
| p53-Independent DNA Damage Response | CDC25A | 95.31190108 | 57 |
| p53-Independent G1/S DNA damage checkpoint | CDC25A | 95.31190108 | 57 |
| Negative regulation of the PI3K/AKT network | INSR | 677.8966832 | 56 |
| PI5P, PP2A and IER3 Regulate PI3K/AKT Signaling | INSR | 677.8966832 | 56 |
| L1CAM interactions | L1CAM | 5562.146873 | 55 |
| G1/S Transition | FBXO5 | 13.356822 | 55 |
| E2F mediated regulation of DNA replication | FBXO5 | 13.356822 | 55 |
| GPVI-mediated activation cascade | AKT3 | 1329.847404 | 54 |
| Negative regulation of the PI3K/AKT network | AKT3 | 1329.847404 | 54 |
| Muscle contraction | ITGB5 | 1237.016858 | 54 |
| Non-integrin membrane-ECM interactions | ITGB5 | 1237.016858 | 54 |
| L1CAM interactions | NUMB | 778.594639 | 54 |
| Muscle contraction | TPM3 | 1617.077441 | 53 |
| Negative regulation of the PI3K/AKT network | ERBB3 | 374.5282722 | 52 |
| Constitutive Signaling by Aberrant PI3K in Cancer | ERBB3 | 374.5282722 | 52 |
| PI5P, PP2A and IER3 Regulate PI3K/AKT Signaling | ERBB3 | 374.5282722 | 52 |
| Muscle contraction | ACTN3 | 2632.567959 | 51 |
| GPVI-mediated activation cascade | CDC42 | 1207.427714 | 51 |
| Muscle contraction | TPM4 | 1135.438734 | 51 |
| C-type lectin receptors (CLRs) | CHUK | 1084.676338 | 51 |
| Negative regulation of the PI3K/AKT network | ERBB4 | 547.423177 | 51 |
| Constitutive Signaling by Aberrant PI3K in Cancer | ERBB4 | 547.423177 | 51 |
| PI5P, PP2A and IER3 Regulate PI3K/AKT Signaling | ERBB4 | 547.423177 | 51 |
| Negative regulation of the PI3K/AKT network | FRS2 | 106.6917073 | 51 |
| Constitutive Signaling by Aberrant PI3K in Cancer | FRS2 | 106.6917073 | 51 |
| PI5P, PP2A and IER3 Regulate PI3K/AKT Signaling | FRS2 | 106.6917073 | 51 |
| Muscle contraction | TPM1 | 471.5593329 | 50 |
| Muscle contraction | TPM2 | 471.5593329 | 50 |
| Regulation of ornithine decarboxylase (ODC) | ODC1 | 453.542024 | 50 |
| GPVI-mediated activation cascade | VAV1 | 296.6205872 | 50 |
| Negative regulation of the PI3K/AKT network | VAV1 | 296.6205872 | 50 |
| Constitutive Signaling by Aberrant PI3K in Cancer | VAV1 | 296.6205872 | 50 |
| PI5P, PP2A and IER3 Regulate PI3K/AKT Signaling | VAV1 | 296.6205872 | 50 |
| L1CAM interactions | ITGA1 | 1148.207094 | 49 |
| Muscle contraction | ITGA1 | 1148.207094 | 49 |
| Negative regulation of the PI3K/AKT network | IRS1 | 836.6656937 | 49 |
| Constitutive Signaling by Aberrant PI3K in Cancer | IRS1 | 836.6656937 | 49 |
| PI5P, PP2A and IER3 Regulate PI3K/AKT Signaling | IRS1 | 836.6656937 | 49 |
| CDT1 association with the CDC6:ORC:origin complex | GMNN | 9.196213496 | 49 |
| Removal of licensing factors from origins | GMNN | 9.196213496 | 49 |
| Muscle contraction | VCL | 1398.987094 | 48 |
| C-type lectin receptors (CLRs) | IKBKG | 1325.690471 | 48 |
| Muscle contraction | DMD | 2161.659856 | 47 |
| Non-integrin membrane-ECM interactions | DMD | 2161.659856 | 47 |
| GPVI-mediated activation cascade | PTPN6 | 544.0692949 | 47 |
| Regulation of ornithine decarboxylase (ODC) | OAZ1 | 258.575615 | 47 |
| Regulation of ornithine decarboxylase (ODC) | OAZ2 | 258.575615 | 47 |
| Negative regulation of the PI3K/AKT network | FGF6 | 89.74827629 | 47 |
| Constitutive Signaling by Aberrant PI3K in Cancer | FGF6 | 89.74827629 | 47 |
| PI5P, PP2A and IER3 Regulate PI3K/AKT Signaling | FGF6 | 89.74827629 | 47 |
| Regulation of ornithine decarboxylase (ODC) | OAZ3 | 258.575615 | 46 |
| Negative regulation of the PI3K/AKT network | FGF1 | 176.9017647 | 46 |
| Constitutive Signaling by Aberrant PI3K in Cancer | FGF1 | 176.9017647 | 46 |
| PI5P, PP2A and IER3 Regulate PI3K/AKT Signaling | FGF1 | 176.9017647 | 46 |
| Negative regulation of the PI3K/AKT network | FGF23 | 13.32564837 | 46 |
| Constitutive Signaling by Aberrant PI3K in Cancer | FGF23 | 13.32564837 | 46 |
| PI5P, PP2A and IER3 Regulate PI3K/AKT Signaling | FGF23 | 13.32564837 | 46 |
| L1CAM interactions | MAP2K1 | 914.3455677 | 45 |
| Negative regulation of the PI3K/AKT network | FGF16 | 10.99578288 | 45 |
| Negative regulation of the PI3K/AKT network | FGF17 | 10.99578288 | 45 |
| Negative regulation of the PI3K/AKT network | FGF18 | 10.99578288 | 45 |
| Negative regulation of the PI3K/AKT network | FGF20 | 10.99578288 | 45 |
| Negative regulation of the PI3K/AKT network | FGF4 | 10.99578288 | 45 |
| Negative regulation of the PI3K/AKT network | FGF8 | 10.99578288 | 45 |
| Negative regulation of the PI3K/AKT network | FGF9 | 10.99578288 | 45 |
| Constitutive Signaling by Aberrant PI3K in Cancer | FGF16 | 10.99578288 | 45 |
| Constitutive Signaling by Aberrant PI3K in Cancer | FGF17 | 10.99578288 | 45 |
| Constitutive Signaling by Aberrant PI3K in Cancer | FGF18 | 10.99578288 | 45 |
| Constitutive Signaling by Aberrant PI3K in Cancer | FGF20 | 10.99578288 | 45 |
| Constitutive Signaling by Aberrant PI3K in Cancer | FGF4 | 10.99578288 | 45 |
| Constitutive Signaling by Aberrant PI3K in Cancer | FGF8 | 10.99578288 | 45 |
| Constitutive Signaling by Aberrant PI3K in Cancer | FGF9 | 10.99578288 | 45 |
| PI5P, PP2A and IER3 Regulate PI3K/AKT Signaling | FGF16 | 10.99578288 | 45 |
| PI5P, PP2A and IER3 Regulate PI3K/AKT Signaling | FGF17 | 10.99578288 | 45 |
| PI5P, PP2A and IER3 Regulate PI3K/AKT Signaling | FGF18 | 10.99578288 | 45 |
| PI5P, PP2A and IER3 Regulate PI3K/AKT Signaling | FGF20 | 10.99578288 | 45 |
| PI5P, PP2A and IER3 Regulate PI3K/AKT Signaling | FGF4 | 10.99578288 | 45 |
| PI5P, PP2A and IER3 Regulate PI3K/AKT Signaling | FGF8 | 10.99578288 | 45 |
| PI5P, PP2A and IER3 Regulate PI3K/AKT Signaling | FGF9 | 10.99578288 | 45 |
| Cardiac conduction | TNNI3 | 3751.195258 | 44 |
| Muscle contraction | TNNI3 | 3751.195258 | 44 |
| Phase 2 - plateau phase | CACNA1C | 3211.563601 | 44 |
| Cardiac conduction | CACNA1C | 3211.563601 | 44 |
| Phase 1 - inactivation of fast Na+ channels | CACNA1C | 3211.563601 | 44 |
| Phase 0 - rapid depolarisation | CACNA1C | 3211.563601 | 44 |
| Muscle contraction | CACNA1C | 3211.563601 | 44 |
| G1/S Transition | RPA2 | 1144.535497 | 44 |
| GPVI-mediated activation cascade | VAV2 | 660.0536806 | 44 |
| L1CAM interactions | VAV2 | 660.0536806 | 44 |
| GPVI-mediated activation cascade | IL2RG | 123.6579794 | 44 |
| Negative regulation of the PI3K/AKT network | FGF5 | 11.1173638 | 44 |
| Constitutive Signaling by Aberrant PI3K in Cancer | FGF5 | 11.1173638 | 44 |
| PI5P, PP2A and IER3 Regulate PI3K/AKT Signaling | FGF5 | 11.1173638 | 44 |
| G1/S Transition | RPA1 | 968.3159265 | 43 |
| Non-integrin membrane-ECM interactions | ITGB4 | 563.4378413 | 43 |
| GPVI-mediated activation cascade | IL2RA | 186.6948789 | 43 |
| GPVI-mediated activation cascade | JAK3 | 59.54821614 | 43 |
| Negative regulation of the PI3K/AKT network | FGF10 | 9.734753527 | 43 |
| Negative regulation of the PI3K/AKT network | FGF22 | 9.734753527 | 43 |
| Negative regulation of the PI3K/AKT network | FGF3 | 9.734753527 | 43 |
| Negative regulation of the PI3K/AKT network | FGF7 | 9.734753527 | 43 |
| Constitutive Signaling by Aberrant PI3K in Cancer | FGF10 | 9.734753527 | 43 |
| Constitutive Signaling by Aberrant PI3K in Cancer | FGF22 | 9.734753527 | 43 |
| Constitutive Signaling by Aberrant PI3K in Cancer | FGF3 | 9.734753527 | 43 |
| Constitutive Signaling by Aberrant PI3K in Cancer | FGF7 | 9.734753527 | 43 |
| PI5P, PP2A and IER3 Regulate PI3K/AKT Signaling | FGF10 | 9.734753527 | 43 |
| PI5P, PP2A and IER3 Regulate PI3K/AKT Signaling | FGF22 | 9.734753527 | 43 |
| PI5P, PP2A and IER3 Regulate PI3K/AKT Signaling | FGF3 | 9.734753527 | 43 |
| PI5P, PP2A and IER3 Regulate PI3K/AKT Signaling | FGF7 | 9.734753527 | 43 |
| L1CAM interactions | ITGA2 | 1592.464912 | 42 |
| Non-integrin membrane-ECM interactions | ITGA2 | 1592.464912 | 42 |
| Negative regulation of the PI3K/AKT network | PPP2R5C | 1345.378871 | 42 |
| PI5P, PP2A and IER3 Regulate PI3K/AKT Signaling | PPP2R5C | 1345.378871 | 42 |
| Muscle contraction | TLN1 | 565.6671898 | 42 |
| Phase 2 - plateau phase | CACNA1D | 435.3120891 | 42 |
| Cardiac conduction | CACNA1D | 435.3120891 | 42 |
| Phase 1 - inactivation of fast Na+ channels | CACNA1D | 435.3120891 | 42 |
| Phase 0 - rapid depolarisation | CACNA1D | 435.3120891 | 42 |
| Muscle contraction | CACNA1D | 435.3120891 | 42 |
| GPVI-mediated activation cascade | IL2 | 239.4016537 | 42 |
| G1/S Transition | RPA3 | 920.902496 | 41 |
| L1CAM interactions | PAK1 | 539.882212 | 41 |
| C-type lectin receptors (CLRs) | PAK1 | 539.882212 | 41 |
| Negative regulation of the PI3K/AKT network | PDGFB | 360.7969141 | 41 |
| Constitutive Signaling by Aberrant PI3K in Cancer | PDGFB | 360.7969141 | 41 |
| PI5P, PP2A and IER3 Regulate PI3K/AKT Signaling | PDGFB | 360.7969141 | 41 |
| Non-integrin membrane-ECM interactions | PDGFB | 360.7969141 | 41 |
| GPVI-mediated activation cascade | IL2RB | 47.3565899 | 41 |
| Negative regulation of the PI3K/AKT network | FGF19 | 10.43634232 | 40 |
| Constitutive Signaling by Aberrant PI3K in Cancer | FGF19 | 10.43634232 | 40 |
| PI5P, PP2A and IER3 Regulate PI3K/AKT Signaling | FGF19 | 10.43634232 | 40 |
| Negative regulation of the PI3K/AKT network | INS | 1279.320142 | 39 |
| PI5P, PP2A and IER3 Regulate PI3K/AKT Signaling | INS | 1279.320142 | 39 |
| Cyclin E associated events during G1/S transition | RB1 | 1237.347683 | 39 |
| Orc1 removal from chromatin | RB1 | 1237.347683 | 39 |
| Switching of origins to a post-replicative state | RB1 | 1237.347683 | 39 |
| Cyclin A:Cdk2-associated events at S phase entry | RB1 | 1237.347683 | 39 |
| G1/S Transition | RB1 | 1237.347683 | 39 |
| E2F mediated regulation of DNA replication | RB1 | 1237.347683 | 39 |
| Removal of licensing factors from origins | RB1 | 1237.347683 | 39 |
| Orc1 removal from chromatin | MCM7 | 864.30294 | 39 |
| Switching of origins to a post-replicative state | MCM7 | 864.30294 | 39 |
| G1/S Transition | MCM7 | 864.30294 | 39 |
| Removal of licensing factors from origins | MCM7 | 864.30294 | 39 |
| Negative regulation of the PI3K/AKT network | PPP2R5B | 707.3967111 | 39 |
| PI5P, PP2A and IER3 Regulate PI3K/AKT Signaling | PPP2R5B | 707.3967111 | 39 |
| Negative regulation of the PI3K/AKT network | PPP2R1B | 634.6014342 | 39 |
| G1/S Transition | PPP2R1B | 634.6014342 | 39 |
| E2F mediated regulation of DNA replication | PPP2R1B | 634.6014342 | 39 |
| PI5P, PP2A and IER3 Regulate PI3K/AKT Signaling | PPP2R1B | 634.6014342 | 39 |
| C-type lectin receptors (CLRs) | IKBKB | 532.104895 | 39 |
| GPVI-mediated activation cascade | RAC2 | 277.7711269 | 39 |
| GPVI-mediated activation cascade | CSF2 | 244.7919863 | 39 |
| GPVI-mediated activation cascade | VAV3 | 103.8166199 | 39 |
| Muscle contraction | VIM | 3933.446767 | 38 |
| C-type lectin receptors (CLRs) | PRKCD | 847.6404471 | 38 |
| Negative regulation of the PI3K/AKT network | PPP2R5A | 586.1990471 | 38 |
| PI5P, PP2A and IER3 Regulate PI3K/AKT Signaling | PPP2R5A | 586.1990471 | 38 |
| L1CAM interactions | ITGA2B | 536.9554041 | 38 |
| Phase 2 - plateau phase | CACNA1S | 280.5985047 | 38 |
| Cardiac conduction | CACNA1S | 280.5985047 | 38 |
| Phase 1 - inactivation of fast Na+ channels | CACNA1S | 280.5985047 | 38 |
| Phase 0 - rapid depolarisation | CACNA1S | 280.5985047 | 38 |
| Muscle contraction | CACNA1S | 280.5985047 | 38 |
| Phase 2 - plateau phase | CACNB2 | 175.3262546 | 38 |
| Phase 2 - plateau phase | CACNB3 | 175.3262546 | 38 |
| Cardiac conduction | CACNB2 | 175.3262546 | 38 |
| Cardiac conduction | CACNB3 | 175.3262546 | 38 |
| Phase 1 - inactivation of fast Na+ channels | CACNB2 | 175.3262546 | 38 |
| Phase 1 - inactivation of fast Na+ channels | CACNB3 | 175.3262546 | 38 |
| Phase 0 - rapid depolarisation | CACNB2 | 175.3262546 | 38 |
| Phase 0 - rapid depolarisation | CACNB3 | 175.3262546 | 38 |
| Muscle contraction | CACNB2 | 175.3262546 | 38 |
| Muscle contraction | CACNB3 | 175.3262546 | 38 |
| Orc1 removal from chromatin | MCM3 | 156.5880455 | 38 |
| Switching of origins to a post-replicative state | MCM3 | 156.5880455 | 38 |
| G1/S Transition | MCM3 | 156.5880455 | 38 |
| Removal of licensing factors from origins | MCM3 | 156.5880455 | 38 |
| Orc1 removal from chromatin | ORC2 | 120.0418551 | 38 |
| Orc1 removal from chromatin | ORC3 | 120.0418551 | 38 |
| Switching of origins to a post-replicative state | ORC2 | 120.0418551 | 38 |
| Switching of origins to a post-replicative state | ORC3 | 120.0418551 | 38 |
| CDT1 association with the CDC6:ORC:origin complex | ORC2 | 120.0418551 | 38 |
| CDT1 association with the CDC6:ORC:origin complex | ORC3 | 120.0418551 | 38 |
| G1/S Transition | ORC2 | 120.0418551 | 38 |
| G1/S Transition | ORC3 | 120.0418551 | 38 |
| E2F mediated regulation of DNA replication | ORC2 | 120.0418551 | 38 |
| E2F mediated regulation of DNA replication | ORC3 | 120.0418551 | 38 |
| Removal of licensing factors from origins | ORC2 | 120.0418551 | 38 |
| Removal of licensing factors from origins | ORC3 | 120.0418551 | 38 |
| Phase 2 - plateau phase | CACNG2 | 93.29989453 | 38 |
| Phase 2 - plateau phase | CACNG3 | 93.29989453 | 38 |
| Phase 2 - plateau phase | CACNG4 | 93.29989453 | 38 |
| Phase 2 - plateau phase | CACNG8 | 93.29989453 | 38 |
| Cardiac conduction | CACNG2 | 93.29989453 | 38 |
| Cardiac conduction | CACNG3 | 93.29989453 | 38 |
| Cardiac conduction | CACNG4 | 93.29989453 | 38 |
| Cardiac conduction | CACNG8 | 93.29989453 | 38 |
| Phase 1 - inactivation of fast Na+ channels | CACNG2 | 93.29989453 | 38 |
| Phase 1 - inactivation of fast Na+ channels | CACNG3 | 93.29989453 | 38 |
| Phase 1 - inactivation of fast Na+ channels | CACNG4 | 93.29989453 | 38 |
| Phase 1 - inactivation of fast Na+ channels | CACNG8 | 93.29989453 | 38 |
| Phase 0 - rapid depolarisation | CACNG2 | 93.29989453 | 38 |
| Phase 0 - rapid depolarisation | CACNG3 | 93.29989453 | 38 |
| Phase 0 - rapid depolarisation | CACNG4 | 93.29989453 | 38 |
| Phase 0 - rapid depolarisation | CACNG8 | 93.29989453 | 38 |
| Muscle contraction | CACNG2 | 93.29989453 | 38 |
| Muscle contraction | CACNG3 | 93.29989453 | 38 |
| Muscle contraction | CACNG4 | 93.29989453 | 38 |
| Muscle contraction | CACNG8 | 93.29989453 | 38 |
| Negative regulation of the PI3K/AKT network | PPP2R5D | 469.494215 | 37 |
| PI5P, PP2A and IER3 Regulate PI3K/AKT Signaling | PPP2R5D | 469.494215 | 37 |
| Non-integrin membrane-ECM interactions | ITGA6 | 462.1552154 | 37 |
| L1CAM interactions | MAP2K2 | 174.4342675 | 37 |
| GPVI-mediated activation cascade | CSF2RB | 130.8313117 | 37 |
| GPVI-mediated activation cascade | LCP2 | 108.0658014 | 37 |
| GPVI-mediated activation cascade | LAT | 74.03497111 | 37 |
| Phase 2 - plateau phase | CACNB1 | 72.89220761 | 37 |
| Phase 2 - plateau phase | CACNB4 | 72.89220761 | 37 |
| Cardiac conduction | CACNB1 | 72.89220761 | 37 |
| Cardiac conduction | CACNB4 | 72.89220761 | 37 |
| Phase 1 - inactivation of fast Na+ channels | CACNB1 | 72.89220761 | 37 |
| Phase 1 - inactivation of fast Na+ channels | CACNB4 | 72.89220761 | 37 |
| Phase 0 - rapid depolarisation | CACNB1 | 72.89220761 | 37 |
| Phase 0 - rapid depolarisation | CACNB4 | 72.89220761 | 37 |
| Muscle contraction | CACNB1 | 72.89220761 | 37 |
| Muscle contraction | CACNB4 | 72.89220761 | 37 |
| Negative regulation of the PI3K/AKT network | IRS2 | 56.75487962 | 37 |
| Constitutive Signaling by Aberrant PI3K in Cancer | IRS2 | 56.75487962 | 37 |
| PI5P, PP2A and IER3 Regulate PI3K/AKT Signaling | IRS2 | 56.75487962 | 37 |
| G1/S Transition | E2F1 | 3890.14687 | 36 |
| E2F mediated regulation of DNA replication | E2F1 | 3890.14687 | 36 |
| Non-integrin membrane-ECM interactions | ACTN1 | 549.0093044 | 36 |
| Muscle contraction | ACTA2 | 505.0142065 | 36 |
| Negative regulation of the PI3K/AKT network | PPP2R5E | 458.9019517 | 36 |
| PI5P, PP2A and IER3 Regulate PI3K/AKT Signaling | PPP2R5E | 458.9019517 | 36 |
| Cyclin E associated events during G1/S transition | CCNE1 | 399.5137612 | 36 |
| Cyclin A:Cdk2-associated events at S phase entry | CCNE1 | 399.5137612 | 36 |
| G1/S Transition | CCNE1 | 399.5137612 | 36 |
| E2F mediated regulation of DNA replication | CCNE1 | 399.5137612 | 36 |
| Orc1 removal from chromatin | MCM2 | 93.83366989 | 36 |
| Switching of origins to a post-replicative state | MCM2 | 93.83366989 | 36 |
| G1/S Transition | MCM2 | 93.83366989 | 36 |
| Removal of licensing factors from origins | MCM2 | 93.83366989 | 36 |
| Orc1 removal from chromatin | MCM6 | 93.81196061 | 36 |
| Switching of origins to a post-replicative state | MCM6 | 93.81196061 | 36 |
| G1/S Transition | MCM6 | 93.81196061 | 36 |
| Removal of licensing factors from origins | MCM6 | 93.81196061 | 36 |
| Orc1 removal from chromatin | ORC4 | 59.36090342 | 36 |
| Switching of origins to a post-replicative state | ORC4 | 59.36090342 | 36 |
| CDT1 association with the CDC6:ORC:origin complex | ORC4 | 59.36090342 | 36 |
| G1/S Transition | ORC4 | 59.36090342 | 36 |
| E2F mediated regulation of DNA replication | ORC4 | 59.36090342 | 36 |
| Removal of licensing factors from origins | ORC4 | 59.36090342 | 36 |
| Phase 2 - plateau phase | CACNA1F | 32.84173075 | 36 |
| Phase 2 - plateau phase | CACNA2D1 | 32.84173075 | 36 |
| Phase 2 - plateau phase | CACNA2D2 | 32.84173075 | 36 |
| Phase 2 - plateau phase | CACNA2D3 | 32.84173075 | 36 |
| Phase 2 - plateau phase | CACNA2D4 | 32.84173075 | 36 |
| Phase 2 - plateau phase | CACNG1 | 32.84173075 | 36 |
| Phase 2 - plateau phase | CACNG5 | 32.84173075 | 36 |
| Phase 2 - plateau phase | CACNG6 | 32.84173075 | 36 |
| Phase 2 - plateau phase | CACNG7 | 32.84173075 | 36 |
| Cardiac conduction | CACNA1F | 32.84173075 | 36 |
| Cardiac conduction | CACNA2D1 | 32.84173075 | 36 |
| Cardiac conduction | CACNA2D2 | 32.84173075 | 36 |
| Cardiac conduction | CACNA2D3 | 32.84173075 | 36 |
| Cardiac conduction | CACNA2D4 | 32.84173075 | 36 |
| Cardiac conduction | CACNG1 | 32.84173075 | 36 |
| Cardiac conduction | CACNG5 | 32.84173075 | 36 |
| Cardiac conduction | CACNG6 | 32.84173075 | 36 |
| Cardiac conduction | CACNG7 | 32.84173075 | 36 |
| Phase 1 - inactivation of fast Na+ channels | CACNA1F | 32.84173075 | 36 |
| Phase 1 - inactivation of fast Na+ channels | CACNA2D1 | 32.84173075 | 36 |
| Phase 1 - inactivation of fast Na+ channels | CACNA2D2 | 32.84173075 | 36 |
| Phase 1 - inactivation of fast Na+ channels | CACNA2D3 | 32.84173075 | 36 |
| Phase 1 - inactivation of fast Na+ channels | CACNA2D4 | 32.84173075 | 36 |
| Phase 1 - inactivation of fast Na+ channels | CACNG1 | 32.84173075 | 36 |
| Phase 1 - inactivation of fast Na+ channels | CACNG5 | 32.84173075 | 36 |
| Phase 1 - inactivation of fast Na+ channels | CACNG6 | 32.84173075 | 36 |
| Phase 1 - inactivation of fast Na+ channels | CACNG7 | 32.84173075 | 36 |
| Phase 0 - rapid depolarisation | CACNA1F | 32.84173075 | 36 |
| Phase 0 - rapid depolarisation | CACNA2D1 | 32.84173075 | 36 |
| Phase 0 - rapid depolarisation | CACNA2D2 | 32.84173075 | 36 |
| Phase 0 - rapid depolarisation | CACNA2D3 | 32.84173075 | 36 |
| Phase 0 - rapid depolarisation | CACNA2D4 | 32.84173075 | 36 |
| Phase 0 - rapid depolarisation | CACNG1 | 32.84173075 | 36 |
| Phase 0 - rapid depolarisation | CACNG5 | 32.84173075 | 36 |
| Phase 0 - rapid depolarisation | CACNG6 | 32.84173075 | 36 |
| Phase 0 - rapid depolarisation | CACNG7 | 32.84173075 | 36 |
| Muscle contraction | CACNA1F | 32.84173075 | 36 |
| Muscle contraction | CACNA2D1 | 32.84173075 | 36 |
| Muscle contraction | CACNA2D2 | 32.84173075 | 36 |
| Muscle contraction | CACNA2D3 | 32.84173075 | 36 |
| Muscle contraction | CACNA2D4 | 32.84173075 | 36 |
| Muscle contraction | CACNG1 | 32.84173075 | 36 |
| Muscle contraction | CACNG5 | 32.84173075 | 36 |
| Muscle contraction | CACNG6 | 32.84173075 | 36 |
| Muscle contraction | CACNG7 | 32.84173075 | 36 |
| Negative regulation of the PI3K/AKT network | KITLG | 1094.873319 | 35 |
| Constitutive Signaling by Aberrant PI3K in Cancer | KITLG | 1094.873319 | 35 |
| PI5P, PP2A and IER3 Regulate PI3K/AKT Signaling | KITLG | 1094.873319 | 35 |
| Muscle contraction | ACTG2 | 485.2090029 | 35 |
| Negative regulation of the PI3K/AKT network | NRG1 | 194.6511598 | 35 |
| Constitutive Signaling by Aberrant PI3K in Cancer | NRG1 | 194.6511598 | 35 |
| PI5P, PP2A and IER3 Regulate PI3K/AKT Signaling | NRG1 | 194.6511598 | 35 |
| Orc1 removal from chromatin | MCM4 | 80.55946639 | 35 |
| Switching of origins to a post-replicative state | MCM4 | 80.55946639 | 35 |
| G1/S Transition | MCM4 | 80.55946639 | 35 |
| Removal of licensing factors from origins | MCM4 | 80.55946639 | 35 |
| Orc1 removal from chromatin | MCM5 | 80.46815096 | 35 |
| Switching of origins to a post-replicative state | MCM5 | 80.46815096 | 35 |
| G1/S Transition | MCM5 | 80.46815096 | 35 |
| Removal of licensing factors from origins | MCM5 | 80.46815096 | 35 |
| Orc1 removal from chromatin | MCM8 | 46.98970439 | 35 |
| Orc1 removal from chromatin | ORC5 | 46.98970439 | 35 |
| Orc1 removal from chromatin | ORC6 | 46.98970439 | 35 |
| Switching of origins to a post-replicative state | MCM8 | 46.98970439 | 35 |
| Switching of origins to a post-replicative state | ORC5 | 46.98970439 | 35 |
| Switching of origins to a post-replicative state | ORC6 | 46.98970439 | 35 |
| CDT1 association with the CDC6:ORC:origin complex | MCM8 | 46.98970439 | 35 |
| CDT1 association with the CDC6:ORC:origin complex | ORC5 | 46.98970439 | 35 |
| CDT1 association with the CDC6:ORC:origin complex | ORC6 | 46.98970439 | 35 |
| G1/S Transition | MCM8 | 46.98970439 | 35 |
| G1/S Transition | ORC5 | 46.98970439 | 35 |
| G1/S Transition | ORC6 | 46.98970439 | 35 |
| E2F mediated regulation of DNA replication | MCM8 | 46.98970439 | 35 |
| E2F mediated regulation of DNA replication | ORC5 | 46.98970439 | 35 |
| E2F mediated regulation of DNA replication | ORC6 | 46.98970439 | 35 |
| Removal of licensing factors from origins | MCM8 | 46.98970439 | 35 |
| Removal of licensing factors from origins | ORC5 | 46.98970439 | 35 |
| Removal of licensing factors from origins | ORC6 | 46.98970439 | 35 |
| C-type lectin receptors (CLRs) | CREBBP | 2218.60119 | 34 |
| L1CAM interactions | AP2A2 | 755.8576034 | 34 |
| L1CAM interactions | AP2A1 | 751.6470874 | 34 |
| G1/S Transition | PCNA | 722.9621477 | 34 |
| E2F mediated regulation of DNA replication | PCNA | 722.9621477 | 34 |
| L1CAM interactions | SH3GL2 | 465.2297505 | 34 |
| G1/S Transition | CCNB1 | 399.2385019 | 34 |
| E2F mediated regulation of DNA replication | CCNB1 | 399.2385019 | 34 |
| C-type lectin receptors (CLRs) | PAK3 | 367.5534013 | 34 |
| Negative regulation of the PI3K/AKT network | KL | 155.1584708 | 34 |
| Constitutive Signaling by Aberrant PI3K in Cancer | KL | 155.1584708 | 34 |
| PI5P, PP2A and IER3 Regulate PI3K/AKT Signaling | KL | 155.1584708 | 34 |
| GPVI-mediated activation cascade | IL3RA | 86.93429471 | 34 |
| Negative regulation of the PI3K/AKT network | KLB | 5.787043148 | 34 |
| Constitutive Signaling by Aberrant PI3K in Cancer | KLB | 5.787043148 | 34 |
| PI5P, PP2A and IER3 Regulate PI3K/AKT Signaling | KLB | 5.787043148 | 34 |
| Muscle contraction | MYL2 | 667.9193935 | 33 |
| G1/S Transition | POLE | 445.430426 | 33 |
| Hedgehog ligand biogenesis | VCP | 382.4347275 | 33 |
| Hh mutants abrogate ligand secretion | VCP | 382.4347275 | 33 |
| Hh mutants that don't undergo autocatalytic processing are degraded by ERAD | VCP | 382.4347275 | 33 |
| Muscle contraction | MYH3 | 161.8989365 | 33 |
| Muscle contraction | MYH6 | 161.8989365 | 33 |
| Muscle contraction | MYH8 | 161.8989365 | 33 |
| Muscle contraction | TTN | 552.4736908 | 32 |
| GPVI-mediated activation cascade | IL5 | 470.0240132 | 32 |
| Muscle contraction | MYL12A | 291.8238412 | 32 |
| Muscle contraction | DES | 170.2546163 | 32 |
| GPVI-mediated activation cascade | CSF2RA | 129.2371863 | 32 |
| G1/S Transition | MCM10 | 95.22225354 | 32 |
| Removal of licensing factors from origins | MCM10 | 95.22225354 | 32 |
| G1/S Transition | POLA1 | 60.09245645 | 32 |
| E2F mediated regulation of DNA replication | POLA1 | 60.09245645 | 32 |
| Non-integrin membrane-ECM interactions | TGFB1 | 2889.927957 | 31 |
| L1CAM interactions | DLG4 | 1819.441936 | 31 |
| L1CAM interactions | LAMC1 | 891.3206679 | 31 |
| Non-integrin membrane-ECM interactions | LAMC1 | 891.3206679 | 31 |
| Muscle contraction | TNNC1 | 621.5833462 | 31 |
| C-type lectin receptors (CLRs) | FBXW11 | 417.9220417 | 31 |
| Orc1 removal from chromatin | CDT1 | 278.1272792 | 31 |
| Switching of origins to a post-replicative state | CDT1 | 278.1272792 | 31 |
| CDT1 association with the CDC6:ORC:origin complex | CDT1 | 278.1272792 | 31 |
| G1/S Transition | CDT1 | 278.1272792 | 31 |
| E2F mediated regulation of DNA replication | CDT1 | 278.1272792 | 31 |
| Removal of licensing factors from origins | CDT1 | 278.1272792 | 31 |
| L1CAM interactions | ITGA5 | 252.5129315 | 31 |
| Muscle contraction | MYH11 | 245.8357898 | 31 |
| G1/S Transition | POLE2 | 81.06723472 | 31 |
| G1/S Transition | CDC7 | 60.80621229 | 31 |
| Muscle contraction | MYL3 | 28.14144442 | 31 |
| Muscle contraction | MYL4 | 28.14144442 | 31 |
| Muscle contraction | MYL1 | 16.6282373 | 31 |
| GPVI-mediated activation cascade | FCER1G | 2573.125259 | 30 |
| C-type lectin receptors (CLRs) | FCER1G | 2573.125259 | 30 |
| Non-integrin membrane-ECM interactions | SDC2 | 1771.294511 | 30 |
| C-type lectin receptors (CLRs) | MAP3K7 | 1094.522256 | 30 |
| Muscle contraction | MYBPC2 | 600.8971224 | 30 |
| Muscle contraction | MYL12B | 194.1347819 | 30 |
| Muscle contraction | MYL6 | 194.1347819 | 30 |
| Muscle contraction | MYL9 | 194.1347819 | 30 |
| Muscle contraction | NEB | 3.493180364 | 30 |
| Muscle contraction | TCAP | 3.493180364 | 30 |
| Muscle contraction | TMOD1 | 3.493180364 | 30 |
| L1CAM interactions | NCAM1 | 1799.559698 | 29 |
| L1CAM interactions | SPTAN1 | 1552.167295 | 29 |
| Cardiac conduction | ATP1B2 | 844.7023585 | 29 |
| Muscle contraction | ATP1B2 | 844.7023585 | 29 |
| Muscle contraction | MYLPF | 677.4136811 | 29 |
| L1CAM interactions | AP2M1 | 369.7297848 | 29 |
| L1CAM interactions | AP2B1 | 357.6048795 | 29 |
| Non-integrin membrane-ECM interactions | LAMA3 | 327.512779 | 29 |
| G1/S Transition | PPP2R3B | 318.7795608 | 29 |
| E2F mediated regulation of DNA replication | PPP2R3B | 318.7795608 | 29 |
| L1CAM interactions | ITGA9 | 217.3082022 | 29 |
| L1CAM interactions | DNM2 | 171.6141635 | 29 |
| GPVI-mediated activation cascade | IL3 | 52.65978353 | 29 |
| G1/S Transition | DBF4 | 10.40785407 | 29 |
| G1/S Transition | POLA2 | 3.68800071 | 29 |
| G1/S Transition | PRIM1 | 3.68800071 | 29 |
| G1/S Transition | PRIM2 | 3.68800071 | 29 |
| E2F mediated regulation of DNA replication | POLA2 | 3.68800071 | 29 |
| E2F mediated regulation of DNA replication | PRIM1 | 3.68800071 | 29 |
| E2F mediated regulation of DNA replication | PRIM2 | 3.68800071 | 29 |
| Muscle contraction | TNNC2 | 3.376595799 | 29 |
| Muscle contraction | MYBPC1 | 3.147403737 | 29 |
| Cardiac conduction | ATP1B3 | 671.8731062 | 28 |
| Muscle contraction | ATP1B3 | 671.8731062 | 28 |
| Cardiac conduction | ATP1B1 | 590.7968727 | 28 |
| Muscle contraction | ATP1B1 | 590.7968727 | 28 |
| L1CAM interactions | AP2S1 | 327.898321 | 28 |
| Negative regulation of the PI3K/AKT network | NRG2 | 57.91385203 | 28 |
| Constitutive Signaling by Aberrant PI3K in Cancer | NRG2 | 57.91385203 | 28 |
| PI5P, PP2A and IER3 Regulate PI3K/AKT Signaling | NRG2 | 57.91385203 | 28 |
| Muscle contraction | TNNT1 | 3.278894649 | 28 |
| Muscle contraction | TNNT2 | 3.278894649 | 28 |
| Muscle contraction | TNNT3 | 3.278894649 | 28 |
| Muscle contraction | MYBPC3 | 3.147403737 | 28 |
| Muscle contraction | TNNI1 | 0.900518272 | 28 |
| Muscle contraction | TNNI2 | 0.900518272 | 28 |
| G1/S Transition | CDC45 | 0 | 28 |
| G1/S Transition | RPA4 | 0 | 28 |
| E2F mediated regulation of DNA replication | CDC45 | 0 | 28 |
| Cardiac conduction | NOS1 | 1172.458864 | 27 |
| Muscle contraction | NOS1 | 1172.458864 | 27 |
| Cardiac conduction | ATP1A1 | 924.7445482 | 27 |
| Muscle contraction | ATP1A1 | 924.7445482 | 27 |
| L1CAM interactions | LAMB1 | 756.2039521 | 27 |
| Non-integrin membrane-ECM interactions | LAMB1 | 756.2039521 | 27 |
| L1CAM interactions | CSNK2A1 | 706.688327 | 27 |
| L1CAM interactions | CLTA | 449.1348136 | 27 |
| Muscle contraction | SORBS1 | 276.6694477 | 27 |
| Non-integrin membrane-ECM interactions | LAMA5 | 273.1918889 | 27 |
| L1CAM interactions | ITGA10 | 183.1997195 | 27 |
| GPVI-mediated activation cascade | IL5RA | 183.1634172 | 27 |
| Cardiac conduction | ATP1A2 | 761.9980589 | 26 |
| Muscle contraction | ATP1A2 | 761.9980589 | 26 |
| Cardiac conduction | ATP1A3 | 753.2349857 | 26 |
| Muscle contraction | ATP1A3 | 753.2349857 | 26 |
| Muscle contraction | CALD1 | 274.4852185 | 26 |
| Negative regulation of the PI3K/AKT network | PDGFA | 187.0456361 | 26 |
| Constitutive Signaling by Aberrant PI3K in Cancer | PDGFA | 187.0456361 | 26 |
| PI5P, PP2A and IER3 Regulate PI3K/AKT Signaling | PDGFA | 187.0456361 | 26 |
| Non-integrin membrane-ECM interactions | PDGFA | 187.0456361 | 26 |
| Cyclin E associated events during G1/S transition | CCNE2 | 48.04774661 | 26 |
| Cyclin A:Cdk2-associated events at S phase entry | CCNE2 | 48.04774661 | 26 |
| G1/S Transition | CCNE2 | 48.04774661 | 26 |
| Muscle contraction | MYL10 | 22.39758935 | 26 |
| Muscle contraction | MYL5 | 22.39758935 | 26 |
| Muscle contraction | MYL7 | 22.39758935 | 26 |
| Cardiac conduction | RYR2 | 2748.094804 | 25 |
| Muscle contraction | RYR2 | 2748.094804 | 25 |
| L1CAM interactions | SPTA1 | 1957.003108 | 25 |
| L1CAM interactions | SCN1B | 1820.813894 | 25 |
| Cardiac conduction | SCN1B | 1820.813894 | 25 |
| Phase 0 - rapid depolarisation | SCN1B | 1820.813894 | 25 |
| Muscle contraction | SCN1B | 1820.813894 | 25 |
| Muscle contraction | MYLK | 1027.645505 | 25 |
| L1CAM interactions | SCN5A | 688.7062481 | 25 |
| Cardiac conduction | SCN5A | 688.7062481 | 25 |
| Phase 0 - rapid depolarisation | SCN5A | 688.7062481 | 25 |
| Muscle contraction | SCN5A | 688.7062481 | 25 |
| Cardiac conduction | ATP1A4 | 671.3961832 | 25 |
| Muscle contraction | ATP1A4 | 671.3961832 | 25 |
| L1CAM interactions | EPHB2 | 329.5781409 | 25 |
| Non-integrin membrane-ECM interactions | LAMC2 | 116.9461118 | 25 |
| C-type lectin receptors (CLRs) | BCL10 | 102.3324664 | 25 |
| Muscle contraction | MYL6B | 3.25838902 | 25 |
| L1CAM interactions | CSNK2A2 | 366.3026651 | 24 |
| Negative regulation of the PI3K/AKT network | PIP5K1C | 122.4297609 | 24 |
| PI5P, PP2A and IER3 Regulate PI3K/AKT Signaling | PIP5K1C | 122.4297609 | 24 |
| Non-integrin membrane-ECM interactions | LAMB2 | 68.67873528 | 24 |
| Muscle contraction | LMOD1 | 0.223871367 | 24 |
| Muscle contraction | SORBS3 | 0.223871367 | 24 |
| Non-integrin membrane-ECM interactions | DAG1 | 2401.497247 | 23 |
| L1CAM interactions | HSPA8 | 746.771095 | 23 |
| C-type lectin receptors (CLRs) | UBE2N | 350.5613182 | 23 |
| L1CAM interactions | LAMA1 | 202.9182132 | 23 |
| Non-integrin membrane-ECM interactions | LAMA1 | 202.9182132 | 23 |
| Cardiac conduction | FXYD2 | 84.30499222 | 23 |
| Muscle contraction | FXYD2 | 84.30499222 | 23 |
| Negative regulation of the PI3K/AKT network | EREG | 51.96538558 | 23 |
| Constitutive Signaling by Aberrant PI3K in Cancer | EREG | 51.96538558 | 23 |
| PI5P, PP2A and IER3 Regulate PI3K/AKT Signaling | EREG | 51.96538558 | 23 |
| Negative regulation of the PI3K/AKT network | CD28 | 32.08760773 | 23 |
| Constitutive Signaling by Aberrant PI3K in Cancer | CD28 | 32.08760773 | 23 |
| PI5P, PP2A and IER3 Regulate PI3K/AKT Signaling | CD28 | 32.08760773 | 23 |
| Negative regulation of the PI3K/AKT network | HBEGF | 18.55605754 | 23 |
| Constitutive Signaling by Aberrant PI3K in Cancer | HBEGF | 18.55605754 | 23 |
| PI5P, PP2A and IER3 Regulate PI3K/AKT Signaling | HBEGF | 18.55605754 | 23 |
| Negative regulation of the PI3K/AKT network | NRG4 | 17.03761423 | 23 |
| Constitutive Signaling by Aberrant PI3K in Cancer | NRG4 | 17.03761423 | 23 |
| PI5P, PP2A and IER3 Regulate PI3K/AKT Signaling | NRG4 | 17.03761423 | 23 |
| Cardiac conduction | ITPR1 | 2200.623003 | 22 |
| C-type lectin receptors (CLRs) | ITPR1 | 2200.623003 | 22 |
| Muscle contraction | ITPR1 | 2200.623003 | 22 |
| L1CAM interactions | ANK2 | 2154.407219 | 22 |
| Non-integrin membrane-ECM interactions | TNC | 841.9756325 | 22 |
| L1CAM interactions | SPTBN2 | 701.8134541 | 22 |
| Non-integrin membrane-ECM interactions | THBS1 | 350.2417544 | 22 |
| Negative regulation of the PI3K/AKT network | BTC | 15.19641075 | 22 |
| Constitutive Signaling by Aberrant PI3K in Cancer | BTC | 15.19641075 | 22 |
| PI5P, PP2A and IER3 Regulate PI3K/AKT Signaling | BTC | 15.19641075 | 22 |
| Muscle contraction | CAV3 | 2124.025741 | 21 |
| C-type lectin receptors (CLRs) | NFATC1 | 484.6883716 | 21 |
| GPVI-mediated activation cascade | RHOB | 303.6106909 | 21 |
| Non-integrin membrane-ECM interactions | AGRN | 257.1009199 | 21 |
| Non-integrin membrane-ECM interactions | SDC4 | 229.5861852 | 21 |
| C-type lectin receptors (CLRs) | MALT1 | 158.8923111 | 21 |
| L1CAM interactions | RPS6KA3 | 95.76727287 | 21 |
| Non-integrin membrane-ECM interactions | LAMA2 | 89.2120111 | 21 |
| Cardiac conduction | SRI | 2798.416107 | 20 |
| Muscle contraction | SRI | 2798.416107 | 20 |
| L1CAM interactions | NRCAM | 1474.807719 | 20 |
| Phase 2 - plateau phase | AKAP9 | 1353.777455 | 20 |
| Cardiac conduction | AKAP9 | 1353.777455 | 20 |
| Muscle contraction | AKAP9 | 1353.777455 | 20 |
| L1CAM interactions | SPTB | 432.1040005 | 20 |
| L1CAM interactions | SPTBN1 | 432.1040005 | 20 |
| L1CAM interactions | SPTBN4 | 432.1040005 | 20 |
| L1CAM interactions | SPTBN5 | 432.1040005 | 20 |
| Negative regulation of the PI3K/AKT network | CD19 | 349.4619235 | 20 |
| Constitutive Signaling by Aberrant PI3K in Cancer | CD19 | 349.4619235 | 20 |
| PI5P, PP2A and IER3 Regulate PI3K/AKT Signaling | CD19 | 349.4619235 | 20 |
| L1CAM interactions | EZR | 293.8705386 | 20 |
| L1CAM interactions | RPS6KA1 | 104.1392087 | 20 |
| C-type lectin receptors (CLRs) | TAB2 | 93.80471943 | 20 |
| Non-integrin membrane-ECM interactions | LAMA4 | 66.18550153 | 20 |
| Cyclin E associated events during G1/S transition | CDK7 | 44.47940666 | 20 |
| Cyclin A:Cdk2-associated events at S phase entry | CDK7 | 44.47940666 | 20 |
| G1/S Transition | CDK7 | 44.47940666 | 20 |
| Negative regulation of the PI3K/AKT network | CD86 | 4.647246028 | 20 |
| Constitutive Signaling by Aberrant PI3K in Cancer | CD86 | 4.647246028 | 20 |
| PI5P, PP2A and IER3 Regulate PI3K/AKT Signaling | CD86 | 4.647246028 | 20 |
| L1CAM interactions | NFASC | 1393.944815 | 19 |
| Cardiac conduction | ITPR3 | 1206.923159 | 19 |
| C-type lectin receptors (CLRs) | ITPR3 | 1206.923159 | 19 |
| Muscle contraction | ITPR3 | 1206.923159 | 19 |
| L1CAM interactions | DLG1 | 551.8277442 | 19 |
| L1CAM interactions | ANK1 | 362.0162074 | 19 |
| C-type lectin receptors (CLRs) | CASP8 | 332.2278951 | 19 |
| L1CAM interactions | CSNK2B | 227.0852687 | 19 |
| Non-integrin membrane-ECM interactions | VTN | 173.7340693 | 19 |
| Non-integrin membrane-ECM interactions | LAMC3 | 61.24529551 | 19 |
| Cyclin E associated events during G1/S transition | MNAT1 | 7.065906996 | 19 |
| Cyclin A:Cdk2-associated events at S phase entry | MNAT1 | 7.065906996 | 19 |
| G1/S Transition | MNAT1 | 7.065906996 | 19 |
| Cardiac conduction | WWTR1 | 524.7661982 | 18 |
| Muscle contraction | WWTR1 | 524.7661982 | 18 |
| C-type lectin receptors (CLRs) | NFATC2 | 340.0044153 | 18 |
| Negative regulation of the PI3K/AKT network | PIP4K2B | 77.69125995 | 18 |
| PI5P, PP2A and IER3 Regulate PI3K/AKT Signaling | PIP4K2B | 77.69125995 | 18 |
| Cyclin E associated events during G1/S transition | PTK6 | 60.41084559 | 18 |
| Cyclin A:Cdk2-associated events at S phase entry | PTK6 | 60.41084559 | 18 |
| G1/S Transition | PTK6 | 60.41084559 | 18 |
| C-type lectin receptors (CLRs) | UBE2V1 | 42.19963487 | 18 |
| C-type lectin receptors (CLRs) | UBE2D2 | 23.03297815 | 18 |
| Negative regulation of the PI3K/AKT network | PIP5K1A | 18.67981771 | 18 |
| PI5P, PP2A and IER3 Regulate PI3K/AKT Signaling | PIP5K1A | 18.67981771 | 18 |
| Cyclin E associated events during G1/S transition | CCNH | 4.45662012 | 18 |
| Cyclin A:Cdk2-associated events at S phase entry | CCNH | 4.45662012 | 18 |
| G1/S Transition | CCNH | 4.45662012 | 18 |
| Non-integrin membrane-ECM interactions | HSPG2 | 1244.294654 | 17 |
| Cardiac conduction | KAT2B | 752.7439614 | 17 |
| Muscle contraction | KAT2B | 752.7439614 | 17 |
| Hedgehog ligand biogenesis | ADAM17 | 291.8497269 | 17 |
| Non-integrin membrane-ECM interactions | SDC1 | 278.1318357 | 17 |
| Ubiquitin Mediated Degradation of Phosphorylated Cdc25A | ATM | 120.7616952 | 17 |
| p53-Independent DNA Damage Response | ATM | 120.7616952 | 17 |
| p53-Independent G1/S DNA damage checkpoint | ATM | 120.7616952 | 17 |
| Cyclin E associated events during G1/S transition | MAX | 94.98167274 | 17 |
| Cyclin A:Cdk2-associated events at S phase entry | MAX | 94.98167274 | 17 |
| G1/S Transition | MAX | 94.98167274 | 17 |
| L1CAM interactions | RPS6KA6 | 71.09908328 | 17 |
| L1CAM interactions | RPS6KA2 | 66.81066113 | 17 |
| Negative regulation of the PI3K/AKT network | PIP5K1B | 13.86152351 | 17 |
| PI5P, PP2A and IER3 Regulate PI3K/AKT Signaling | PIP5K1B | 13.86152351 | 17 |
| Negative regulation of the PI3K/AKT network | PIP4K2A | 9.832168378 | 17 |
| Negative regulation of the PI3K/AKT network | PIP4K2C | 9.832168378 | 17 |
| PI5P, PP2A and IER3 Regulate PI3K/AKT Signaling | PIP4K2A | 9.832168378 | 17 |
| PI5P, PP2A and IER3 Regulate PI3K/AKT Signaling | PIP4K2C | 9.832168378 | 17 |
| Cardiac conduction | ITPR2 | 662.9930716 | 16 |
| C-type lectin receptors (CLRs) | ITPR2 | 662.9930716 | 16 |
| Muscle contraction | ITPR2 | 662.9930716 | 16 |
| L1CAM interactions | RPS6KA5 | 159.9076934 | 16 |
| C-type lectin receptors (CLRs) | RPS6KA5 | 159.9076934 | 16 |
| Ubiquitin Mediated Degradation of Phosphorylated Cdc25A | CHEK1 | 149.9444091 | 16 |
| p53-Independent DNA Damage Response | CHEK1 | 149.9444091 | 16 |
| p53-Independent G1/S DNA damage checkpoint | CHEK1 | 149.9444091 | 16 |
| Cardiac conduction | FGF11 | 142.9605022 | 16 |
| Cardiac conduction | FGF12 | 142.9605022 | 16 |
| Cardiac conduction | FGF13 | 142.9605022 | 16 |
| Cardiac conduction | FGF14 | 142.9605022 | 16 |
| Phase 0 - rapid depolarisation | FGF11 | 142.9605022 | 16 |
| Phase 0 - rapid depolarisation | FGF12 | 142.9605022 | 16 |
| Phase 0 - rapid depolarisation | FGF13 | 142.9605022 | 16 |
| Phase 0 - rapid depolarisation | FGF14 | 142.9605022 | 16 |
| Muscle contraction | FGF11 | 142.9605022 | 16 |
| Muscle contraction | FGF12 | 142.9605022 | 16 |
| Muscle contraction | FGF13 | 142.9605022 | 16 |
| Muscle contraction | FGF14 | 142.9605022 | 16 |
| G1/S Transition | TFDP1 | 40.00243831 | 16 |
| E2F mediated regulation of DNA replication | TFDP1 | 40.00243831 | 16 |
| Non-integrin membrane-ECM interactions | LAMB3 | 18.15564244 | 16 |
| Negative regulation of the PI3K/AKT network | CD80 | 3.302168985 | 16 |
| Constitutive Signaling by Aberrant PI3K in Cancer | CD80 | 3.302168985 | 16 |
| PI5P, PP2A and IER3 Regulate PI3K/AKT Signaling | CD80 | 3.302168985 | 16 |
| Cardiac conduction | PLN | 1373.33139 | 15 |
| Muscle contraction | PLN | 1373.33139 | 15 |
| C-type lectin receptors (CLRs) | IL1B | 184.7661919 | 15 |
| C-type lectin receptors (CLRs) | CARD11 | 11.52129863 | 15 |
| Cardiac conduction | SLC8A1 | 328.8980648 | 14 |
| Muscle contraction | SLC8A1 | 328.8980648 | 14 |
| C-type lectin receptors (CLRs) | UBE2M | 0.598562778 | 14 |
| Cross-presentation of soluble exogenous antigens (endosomes) | FCGR1A | 0.036235448 | 14 |
| Hedgehog ligand biogenesis | SEL1L | 930.2106466 | 13 |
| Hh mutants abrogate ligand secretion | SEL1L | 930.2106466 | 13 |
| Hh mutants that don't undergo autocatalytic processing are degraded by ERAD | SEL1L | 930.2106466 | 13 |
| L1CAM interactions | ANK3 | 115.0389016 | 13 |
| GPVI-mediated activation cascade | RHOG | 53.25404145 | 13 |
| Cyclin E associated events during G1/S transition | CCNA2 | 45.65963657 | 13 |
| Orc1 removal from chromatin | CCNA2 | 45.65963657 | 13 |
| Switching of origins to a post-replicative state | CCNA2 | 45.65963657 | 13 |
| Cyclin A:Cdk2-associated events at S phase entry | CCNA2 | 45.65963657 | 13 |
| G1/S Transition | CCNA2 | 45.65963657 | 13 |
| Removal of licensing factors from origins | CCNA2 | 45.65963657 | 13 |
| C-type lectin receptors (CLRs) | TAB1 | 42.18519278 | 13 |
| C-type lectin receptors (CLRs) | UBA3 | 0 | 13 |
| C-type lectin receptors (CLRs) | NFATC3 | 75.99905517 | 12 |
| L1CAM interactions | DNM1 | 9.161740863 | 12 |
| C-type lectin receptors (CLRs) | TAB3 | 1.46419826 | 12 |
| Muscle contraction | ANXA1 | 1719.423046 | 11 |
| Cardiac conduction | GATA4 | 294.490967 | 11 |
| Muscle contraction | GATA4 | 294.490967 | 11 |
| C-type lectin receptors (CLRs) | PPP3CB | 127.4485858 | 11 |
| Cyclin A:Cdk2-associated events at S phase entry | CDC25B | 77.26114824 | 11 |
| L1CAM interactions | RPS6KA4 | 59.41227251 | 11 |
| Cyclin E associated events during G1/S transition | PKMYT1 | 51.56129411 | 11 |
| G1/S Transition | PKMYT1 | 51.56129411 | 11 |
| Hedgehog ligand biogenesis | SYVN1 | 47.64972512 | 11 |
| Hh mutants abrogate ligand secretion | SYVN1 | 47.64972512 | 11 |
| Hh mutants that don't undergo autocatalytic processing are degraded by ERAD | SYVN1 | 47.64972512 | 11 |
| Cyclin E associated events during G1/S transition | CCNA1 | 12.42238973 | 11 |
| Orc1 removal from chromatin | CCNA1 | 12.42238973 | 11 |
| Switching of origins to a post-replicative state | CCNA1 | 12.42238973 | 11 |
| Cyclin A:Cdk2-associated events at S phase entry | CCNA1 | 12.42238973 | 11 |
| G1/S Transition | CCNA1 | 12.42238973 | 11 |
| E2F mediated regulation of DNA replication | CCNA1 | 12.42238973 | 11 |
| Removal of licensing factors from origins | CCNA1 | 12.42238973 | 11 |
| L1CAM interactions | KCNQ2 | 1.843765025 | 11 |
| L1CAM interactions | KCNQ3 | 1.843765025 | 11 |
| Cardiac conduction | FXYD1 | 0.531717597 | 11 |
| Muscle contraction | FXYD1 | 0.531717597 | 11 |
| L1CAM interactions | MSN | 497.5747886 | 10 |
| Cardiac conduction | ATP2A2 | 112.041284 | 10 |
| Muscle contraction | ATP2A2 | 112.041284 | 10 |
| Cardiac conduction | RANGRF | 0.75 | 10 |
| Phase 0 - rapid depolarisation | RANGRF | 0.75 | 10 |
| Muscle contraction | RANGRF | 0.75 | 10 |
| Hedgehog ligand biogenesis | DERL2 | 0.222222222 | 10 |
| Hh mutants abrogate ligand secretion | DERL2 | 0.222222222 | 10 |
| Hh mutants that don't undergo autocatalytic processing are degraded by ERAD | DERL2 | 0.222222222 | 10 |
| L1CAM interactions | NRP1 | 91.17878646 | 9 |
| L1CAM interactions | RDX | 70.80682188 | 9 |
| Cardiac conduction | HIPK2 | 66.29578092 | 9 |
| Muscle contraction | HIPK2 | 66.29578092 | 9 |
| C-type lectin receptors (CLRs) | PPP3CA | 61.31076502 | 9 |
| L1CAM interactions | KIF4A | 8.943883463 | 9 |
| L1CAM interactions | KIF4B | 8.943883463 | 9 |
| Non-integrin membrane-ECM interactions | NTN4 | 1.528782498 | 9 |
| Cardiac conduction | SLN | 1.016666667 | 9 |
| Muscle contraction | SLN | 1.016666667 | 9 |
| Hedgehog ligand biogenesis | ERLEC1 | 0 | 9 |
| Hedgehog ligand biogenesis | OS9 | 0 | 9 |
| Hh mutants abrogate ligand secretion | ERLEC1 | 0 | 9 |
| Hh mutants abrogate ligand secretion | OS9 | 0 | 9 |
| Hh mutants that don't undergo autocatalytic processing are degraded by ERAD | ERLEC1 | 0 | 9 |
| Hh mutants that don't undergo autocatalytic processing are degraded by ERAD | OS9 | 0 | 9 |
| Cardiac conduction | RYR3 | 1256.618701 | 8 |
| Muscle contraction | RYR3 | 1256.618701 | 8 |
| L1CAM interactions | CHL1 | 1221.731439 | 8 |
| Cardiac conduction | RYR1 | 820.0097938 | 8 |
| Muscle contraction | RYR1 | 820.0097938 | 8 |
| Cardiac conduction | ATP2B4 | 203.7566746 | 8 |
| Muscle contraction | ATP2B4 | 203.7566746 | 8 |
| C-type lectin receptors (CLRs) | PPP3R1 | 126.0042912 | 8 |
| Cardiac conduction | FKBP1B | 79.01261415 | 8 |
| Muscle contraction | FKBP1B | 79.01261415 | 8 |
| Cardiac conduction | ATP2A1 | 58.58459405 | 8 |
| Cardiac conduction | ATP2A3 | 58.58459405 | 8 |
| Muscle contraction | ATP2A1 | 58.58459405 | 8 |
| Muscle contraction | ATP2A3 | 58.58459405 | 8 |
| Ubiquitin Mediated Degradation of Phosphorylated Cdc25A | CHEK2 | 13.22835113 | 8 |
| p53-Independent DNA Damage Response | CHEK2 | 13.22835113 | 8 |
| p53-Independent G1/S DNA damage checkpoint | CHEK2 | 13.22835113 | 8 |
| Cardiac conduction | FXYD7 | 6.453635999 | 8 |
| Muscle contraction | FXYD7 | 6.453635999 | 8 |
| Negative regulation of the PI3K/AKT network | TRAT1 | 0.164932231 | 8 |
| Constitutive Signaling by Aberrant PI3K in Cancer | TRAT1 | 0.164932231 | 8 |
| PI5P, PP2A and IER3 Regulate PI3K/AKT Signaling | TRAT1 | 0.164932231 | 8 |
| Cardiac conduction | KCNJ11 | 1217.290319 | 7 |
| Muscle contraction | KCNJ11 | 1217.290319 | 7 |
| L1CAM interactions | DLG3 | 392.701881 | 7 |
| Non-integrin membrane-ECM interactions | CASK | 290.5533841 | 7 |
| Cardiac conduction | KCNH2 | 211.3380465 | 7 |
| Muscle contraction | KCNH2 | 211.3380465 | 7 |
| Muscle contraction | DYSF | 151.9496406 | 7 |
| C-type lectin receptors (CLRs) | CD209 | 130.5765697 | 7 |
| Muscle contraction | TRIM72 | 104.0324879 | 7 |
| L1CAM interactions | SDCBP | 56.93579468 | 7 |
| Cardiac conduction | TBX5 | 50.17811867 | 7 |
| Muscle contraction | TBX5 | 50.17811867 | 7 |
| Cardiac conduction | NKX2-5 | 12.29408239 | 7 |
| Muscle contraction | NKX2-5 | 12.29408239 | 7 |
| Negative regulation of the PI3K/AKT network | IER3 | 2.964662162 | 7 |
| PI5P, PP2A and IER3 Regulate PI3K/AKT Signaling | IER3 | 2.964662162 | 7 |
| L1CAM interactions | SCN4B | 1.856578911 | 7 |
| L1CAM interactions | SCN7A | 1.856578911 | 7 |
| Cardiac conduction | SCN4B | 1.856578911 | 7 |
| Cardiac conduction | SCN7A | 1.856578911 | 7 |
| Phase 0 - rapid depolarisation | SCN4B | 1.856578911 | 7 |
| Phase 0 - rapid depolarisation | SCN7A | 1.856578911 | 7 |
| Muscle contraction | SCN4B | 1.856578911 | 7 |
| Muscle contraction | SCN7A | 1.856578911 | 7 |
| Cardiac conduction | ASPH | 1.762722368 | 7 |
| Cardiac conduction | CLIC2 | 1.762722368 | 7 |
| Cardiac conduction | TRDN | 1.762722368 | 7 |
| Muscle contraction | ASPH | 1.762722368 | 7 |
| Muscle contraction | CLIC2 | 1.762722368 | 7 |
| Muscle contraction | TRDN | 1.762722368 | 7 |
| Cardiac conduction | FXYD3 | 0.476923077 | 7 |
| Cardiac conduction | FXYD4 | 0.476923077 | 7 |
| Cardiac conduction | FXYD6 | 0.476923077 | 7 |
| Muscle contraction | FXYD3 | 0.476923077 | 7 |
| Muscle contraction | FXYD4 | 0.476923077 | 7 |
| Muscle contraction | FXYD6 | 0.476923077 | 7 |
| L1CAM interactions | CNTN1 | 312.9911292 | 6 |
| Phase 2 - plateau phase | KCNE1 | 123.4392532 | 6 |
| Cardiac conduction | KCNE1 | 123.4392532 | 6 |
| Muscle contraction | KCNE1 | 123.4392532 | 6 |
| Phase 2 - plateau phase | KCNQ1 | 111.539412 | 6 |
| Cardiac conduction | KCNQ1 | 111.539412 | 6 |
| Muscle contraction | KCNQ1 | 111.539412 | 6 |
| Non-integrin membrane-ECM interactions | SDC3 | 76.39325487 | 6 |
| GPVI-mediated activation cascade | PDPN | 19.75787684 | 6 |
| Cardiac conduction | HIPK1 | 17.09668708 | 6 |
| Muscle contraction | HIPK1 | 17.09668708 | 6 |
| Cardiac conduction | STIM1 | 15.54611466 | 6 |
| Muscle contraction | STIM1 | 15.54611466 | 6 |
| L1CAM interactions | DPYSL2 | 7.758520613 | 6 |
| Cyclin E associated events during G1/S transition | WEE1 | 4.1348048 | 6 |
| Cyclin A:Cdk2-associated events at S phase entry | WEE1 | 4.1348048 | 6 |
| G1/S Transition | WEE1 | 4.1348048 | 6 |
| Negative regulation of the PI3K/AKT network | NRG3 | 2.874293005 | 6 |
| Constitutive Signaling by Aberrant PI3K in Cancer | NRG3 | 2.874293005 | 6 |
| PI5P, PP2A and IER3 Regulate PI3K/AKT Signaling | NRG3 | 2.874293005 | 6 |
| C-type lectin receptors (CLRs) | CARD9 | 2.705332236 | 6 |
| GPVI-mediated activation cascade | GP6 | 0 | 6 |
| Cardiac conduction | ATP2B1 | 21.06255974 | 5 |
| Muscle contraction | ATP2B1 | 21.06255974 | 5 |
| Negative regulation of the PI3K/AKT network | TRIB3 | 1.61850909 | 5 |
| C-type lectin receptors (CLRs) | PYCARD | 0.366666667 | 5 |
| GPVI-mediated activation cascade | CLEC1B | 0 | 5 |
| C-type lectin receptors (CLRs) | CLEC4E | 0 | 5 |
| C-type lectin receptors (CLRs) | CLEC6A | 0 | 5 |
| Muscle contraction | ANXA2 | 0 | 5 |
| Muscle contraction | ANXA6 | 0 | 5 |
| Cardiac conduction | NPPA | 4678 | 4 |
| Muscle contraction | NPPA | 4678 | 4 |
| Cardiac conduction | KCNJ2 | 2354.580454 | 4 |
| Muscle contraction | KCNJ2 | 2354.580454 | 4 |
| Cardiac conduction | ORAI1 | 60.69158365 | 4 |
| Muscle contraction | ORAI1 | 60.69158365 | 4 |
| C-type lectin receptors (CLRs) | ICAM3 | 30.10842159 | 4 |
| Non-integrin membrane-ECM interactions | NRXN1 | 27.989218 | 4 |
| L1CAM interactions | CNTN2 | 12.93011858 | 4 |
| L1CAM interactions | NCAN | 10.50005138 | 4 |
| Cardiac conduction | KCND1 | 3.333333333 | 4 |
| Cardiac conduction | KCND2 | 3.333333333 | 4 |
| Cardiac conduction | KCND3 | 3.333333333 | 4 |
| Phase 1 - inactivation of fast Na+ channels | KCND1 | 3.333333333 | 4 |
| Phase 1 - inactivation of fast Na+ channels | KCND2 | 3.333333333 | 4 |
| Phase 1 - inactivation of fast Na+ channels | KCND3 | 3.333333333 | 4 |
| Muscle contraction | KCND1 | 3.333333333 | 4 |
| Muscle contraction | KCND2 | 3.333333333 | 4 |
| Muscle contraction | KCND3 | 3.333333333 | 4 |
| Cardiac conduction | KCNIP2 | 1.5 | 4 |
| Cardiac conduction | KCNIP4 | 1.5 | 4 |
| Phase 1 - inactivation of fast Na+ channels | KCNIP2 | 1.5 | 4 |
| Phase 1 - inactivation of fast Na+ channels | KCNIP4 | 1.5 | 4 |
| Muscle contraction | KCNIP2 | 1.5 | 4 |
| Muscle contraction | KCNIP4 | 1.5 | 4 |
| Non-integrin membrane-ECM interactions | DDR2 | 1.453407188 | 4 |
| G1/S Transition | RRM2 | 1.297670348 | 4 |
| E2F mediated regulation of DNA replication | RRM2 | 1.297670348 | 4 |
| L1CAM interactions | RANBP9 | 1.24044798 | 4 |
| Cardiac conduction | TRPC1 | 0.787179487 | 4 |
| Muscle contraction | TRPC1 | 0.787179487 | 4 |
| Cardiac conduction | AHCYL1 | 0.5 | 4 |
| C-type lectin receptors (CLRs) | AHCYL1 | 0.5 | 4 |
| Muscle contraction | AHCYL1 | 0.5 | 4 |
| Regulation of ornithine decarboxylase (ODC) | AZIN1 | 0.086956522 | 4 |
| Cardiac conduction | ATP2B2 | 0.054794521 | 4 |
| Muscle contraction | ATP2B2 | 0.054794521 | 4 |
| GPVI-mediated activation cascade | PIK3R6 | 0 | 4 |
| Negative regulation of the PI3K/AKT network | PHLPP1 | 0 | 4 |
| Negative regulation of the PI3K/AKT network | PHLPP2 | 0 | 4 |
| C-type lectin receptors (CLRs) | CDC34 | 0 | 4 |
| C-type lectin receptors (CLRs) | ICAM2 | 51.558408 | 3 |
| L1CAM interactions | CNTNAP1 | 18.96515027 | 3 |
| Phase 2 - plateau phase | KCNE2 | 8.178322027 | 3 |
| Cardiac conduction | KCNE2 | 8.178322027 | 3 |
| Muscle contraction | KCNE2 | 8.178322027 | 3 |
| Cardiac conduction | KCNIP1 | 1.5 | 3 |
| Cardiac conduction | KCNIP3 | 1.5 | 3 |
| Phase 1 - inactivation of fast Na+ channels | KCNIP1 | 1.5 | 3 |
| Phase 1 - inactivation of fast Na+ channels | KCNIP3 | 1.5 | 3 |
| Muscle contraction | KCNIP1 | 1.5 | 3 |
| Muscle contraction | KCNIP3 | 1.5 | 3 |
| L1CAM interactions | ALCAM | 0.096574625 | 3 |
| L1CAM interactions | SCN1A | 0.054794521 | 3 |
| Cardiac conduction | ATP2B3 | 0.054794521 | 3 |
| Cardiac conduction | SCN1A | 0.054794521 | 3 |
| Phase 0 - rapid depolarisation | SCN1A | 0.054794521 | 3 |
| Muscle contraction | ATP2B3 | 0.054794521 | 3 |
| Muscle contraction | SCN1A | 0.054794521 | 3 |
| Negative regulation of the PI3K/AKT network | THEM4 | 0 | 3 |
| C-type lectin receptors (CLRs) | CLEC7A | 0 | 3 |
| Cardiac conduction | NPR1 | 2344 | 2 |
| Muscle contraction | NPR1 | 2344 | 2 |
| Hedgehog ligand biogenesis | HHAT | 1174 | 2 |
| Cardiac conduction | KCNK5 | 1174 | 2 |
| Cardiac conduction | NPPC | 1174 | 2 |
| G1/S Transition | TYMS | 1174 | 2 |
| E2F mediated regulation of DNA replication | TYMS | 1174 | 2 |
| Muscle contraction | KCNK5 | 1174 | 2 |
| Muscle contraction | NPPC | 1174 | 2 |
| Regulation of ornithine decarboxylase (ODC) | NQO1 | 3.47606888 | 2 |
| L1CAM interactions | CD24 | 1.455294045 | 2 |
| Hedgehog ligand biogenesis | DISP2 | 0 | 2 |
| Hedgehog ligand biogenesis | GPC5 | 0 | 2 |
| Hedgehog ligand biogenesis | NOTUM | 0 | 2 |
| Hedgehog ligand biogenesis | SCUBE2 | 0 | 2 |
| Hedgehog ligand biogenesis | DHH | 0 | 1 |
| Hedgehog ligand biogenesis | P4HB | 0 | 1 |
| Cross-presentation of soluble exogenous antigens (endosomes) | MRC1 | 0 | 1 |
| Cross-presentation of soluble exogenous antigens (endosomes) | MRC2 | 0 | 1 |
| L1CAM interactions | CNTN6 | 0 | 1 |
| L1CAM interactions | DCX | 0 | 1 |
| L1CAM interactions | NRP2 | 0 | 1 |
| L1CAM interactions | SCN2A | 0 | 1 |
| L1CAM interactions | SCN2B | 0 | 1 |
| L1CAM interactions | SHTN1 | 0 | 1 |
| Cardiac conduction | ABCC9 | 0 | 1 |
| Cardiac conduction | CORIN | 0 | 1 |
| Cardiac conduction | DMPK | 0 | 1 |
| Cardiac conduction | KCNJ12 | 0 | 1 |
| Cardiac conduction | KCNJ4 | 0 | 1 |
| Cardiac conduction | KCNK3 | 0 | 1 |
| Cardiac conduction | KCNK9 | 0 | 1 |
| Cardiac conduction | NPR2 | 0 | 1 |
| Cardiac conduction | SCN2A | 0 | 1 |
| Cardiac conduction | SCN2B | 0 | 1 |
| Cardiac conduction | SLC8A2 | 0 | 1 |
| Cardiac conduction | SLC8A3 | 0 | 1 |
| G1/S Transition | DHFR | 0 | 1 |
| Hh mutants abrogate ligand secretion | DHH | 0 | 1 |
| E2F mediated regulation of DNA replication | DHFR | 0 | 1 |
| Phase 0 - rapid depolarisation | SCN2A | 0 | 1 |
| Phase 0 - rapid depolarisation | SCN2B | 0 | 1 |
| C-type lectin receptors (CLRs) | CLEC4C | 0 | 1 |
| C-type lectin receptors (CLRs) | CLEC4D | 0 | 1 |
| Muscle contraction | ABCC9 | 0 | 1 |
| Muscle contraction | CORIN | 0 | 1 |
| Muscle contraction | DMPK | 0 | 1 |
| Muscle contraction | KCNJ12 | 0 | 1 |
| Muscle contraction | KCNJ4 | 0 | 1 |
| Muscle contraction | KCNK3 | 0 | 1 |
| Muscle contraction | KCNK9 | 0 | 1 |
| Muscle contraction | NPR2 | 0 | 1 |
| Muscle contraction | SCN2A | 0 | 1 |
| Muscle contraction | SCN2B | 0 | 1 |
| Muscle contraction | SLC8A2 | 0 | 1 |
| Muscle contraction | SLC8A3 | 0 | 1 |
| Non-integrin membrane-ECM interactions | TRAPPC4 | 0 | 1 |

**Supplementary Table5.** A full list of all significantly enriched biological processes with an FDR < 0.001.

| **p-value** | **q-value** | **term_goid** | **term_name** |
| --- | --- | --- | --- |
| 1.05E-110 | 2.18E-108 | GO:0002220 | innate immune response activating cell surface receptor signaling pathway |
| 1.59E-110 | 2.18E-108 | GO:0006521 | regulation of cellular amino acid metabolic process |
| 2.30E-108 | 8.12E-106 | GO:0038061 | NIK/NF-kappaB signaling |
| 2.58E-105 | 2.37E-103 | GO:0070498 | interleukin-1-mediated signaling pathway |
| 2.46E-100 | 1.69E-98 | GO:0033238 | regulation of cellular amine metabolic process |
| 2.88E-88 | 5.08E-86 | GO:0043620 | regulation of DNA-templated transcription in response to stress |
| 3.32E-87 | 1.82E-85 | GO:0043618 | regulation of transcription from RNA polymerase II promoter in response to stress |
| 4.45E-86 | 5.24E-84 | GO:0044106 | cellular amine metabolic process |
| 2.33E-85 | 1.07E-83 | GO:0070555 | response to interleukin-1 |
| 4.43E-85 | 3.91E-83 | GO:0002758 | innate immune response-activating signal transduction |
| 3.16E-84 | 1.24E-82 | GO:1901532 | regulation of hematopoietic progenitor cell differentiation |
| 3.45E-84 | 2.43E-82 | GO:0009308 | amine metabolic process |
| 5.49E-84 | 1.83E-82 | GO:0060218 | hematopoietic stem cell differentiation |
| 6.00E-84 | 1.83E-82 | GO:0042590 | antigen processing and presentation of exogenous peptide antigen via MHC class I |
| 1.78E-83 | 1.05E-81 | GO:0044839 | cell cycle G2/M phase transition |
| 3.06E-83 | 6.45E-81 | GO:0002218 | activation of innate immune response |
| 3.10E-83 | 8.52E-82 | GO:0000086 | G2/M transition of mitotic cell cycle |
| 9.67E-82 | 1.02E-79 | GO:0007164 | establishment of tissue polarity |
| 9.67E-82 | 4.88E-80 | GO:0001736 | establishment of planar polarity |
| 4.21E-81 | 1.86E-79 | GO:0071456 | cellular response to hypoxia |
| 3.66E-80 | 9.16E-79 | GO:0036294 | cellular response to decreased oxygen levels |
| 1.00E-79 | 2.29E-78 | GO:0033209 | tumor necrosis factor-mediated signaling pathway |
| 4.78E-79 | 1.01E-77 | GO:0010565 | regulation of cellular ketone metabolic process |
| 9.93E-79 | 3.89E-77 | GO:0002474 | antigen processing and presentation of peptide antigen via MHC class I |
| 1.51E-78 | 2.98E-77 | GO:0050851 | antigen receptor-mediated signaling pathway |
| 2.04E-78 | 3.74E-77 | GO:0001738 | morphogenesis of a polarized epithelium |
| 3.76E-78 | 1.33E-76 | GO:0071453 | cellular response to oxygen levels |
| 4.91E-78 | 8.44E-77 | GO:0010498 | proteasomal protein catabolic process |
| 6.99E-78 | 1.13E-76 | GO:2000736 | regulation of stem cell differentiation |
| 9.95E-78 | 3.19E-76 | GO:0030163 | protein catabolic process |
| 1.58E-76 | 2.42E-75 | GO:1905330 | regulation of morphogenesis of an epithelium |
| 4.60E-75 | 1.35E-73 | GO:0044772 | mitotic cell cycle phase transition |
| 6.07E-75 | 1.65E-73 | GO:0043488 | regulation of mRNA stability |
| 6.91E-75 | 4.86E-73 | GO:0044770 | cell cycle phase transition |
| 9.21E-74 | 4.86E-72 | GO:0043487 | regulation of RNA stability |
| 1.06E-73 | 1.53E-72 | GO:0045088 | regulation of innate immune response |
| 1.43E-73 | 3.60E-72 | GO:0002244 | hematopoietic progenitor cell differentiation |
| 7.49E-73 | 1.03E-71 | GO:0043632 | modification-dependent macromolecule catabolic process |
| 1.06E-72 | 1.39E-71 | GO:0070646 | protein modification by small protein removal |
| 1.96E-71 | 2.45E-70 | GO:0031349 | positive regulation of defense response |
| 4.38E-71 | 1.03E-69 | GO:0002429 | immune response-activating cell surface receptor signaling pathway |
| 1.36E-70 | 5.72E-69 | GO:0042180 | cellular ketone metabolic process |
| 1.45E-69 | 1.73E-68 | GO:0051603 | proteolysis involved in cellular protein catabolic process |
| 2.79E-69 | 3.20E-68 | GO:0044257 | cellular protein catabolic process |
| 3.42E-69 | 7.54E-68 | GO:0002768 | immune response-regulating cell surface receptor signaling pathway |
| 5.74E-69 | 6.32E-68 | GO:0030111 | regulation of Wnt signaling pathway |
| 1.60E-68 | 5.63E-67 | GO:0001666 | response to hypoxia |
| 1.74E-68 | 1.84E-67 | GO:2000027 | regulation of organ morphogenesis |
| 3.93E-68 | 1.19E-66 | GO:0070482 | response to oxygen levels |
| 5.38E-68 | 1.12E-66 | GO:1901565 | organonitrogen compound catabolic process |
| 6.09E-68 | 1.19E-66 | GO:0036293 | response to decreased oxygen levels |
| 6.92E-68 | 7.04E-67 | GO:0016055 | Wnt signaling pathway |
| 8.85E-68 | 2.33E-66 | GO:0198738 | cell-cell signaling by wnt |
| 1.44E-67 | 1.42E-66 | GO:0034612 | response to tumor necrosis factor |
| 9.73E-66 | 1.81E-64 | GO:0009057 | macromolecule catabolic process |
| 8.10E-65 | 1.43E-63 | GO:0048863 | stem cell differentiation |
| 1.18E-64 | 1.98E-63 | GO:0002478 | antigen processing and presentation of exogenous peptide antigen |
| 2.47E-64 | 2.34E-63 | GO:0045786 | negative regulation of cell cycle |
| 8.44E-64 | 1.98E-62 | GO:0019884 | antigen processing and presentation of exogenous antigen |
| 1.65E-63 | 3.49E-62 | GO:0002757 | immune response-activating signal transduction |
| 5.54E-63 | 1.06E-61 | GO:0048002 | antigen processing and presentation of peptide antigen |
| 2.70E-62 | 4.34E-61 | GO:1905114 | cell surface receptor signaling pathway involved in cell-cell signaling |
| 4.27E-62 | 7.50E-61 | GO:0002764 | immune response-regulating signaling pathway |
| 6.91E-62 | 1.12E-60 | GO:1903047 | mitotic cell cycle process |
| 2.17E-61 | 2.02E-59 | GO:0019882 | antigen processing and presentation |
| 3.10E-61 | 4.68E-60 | GO:0006520 | cellular amino acid metabolic process |
| 5.31E-61 | 7.47E-60 | GO:0000278 | mitotic cell cycle |
| 6.98E-61 | 6.40E-60 | GO:0007346 | regulation of mitotic cell cycle |
| 2.22E-60 | 1.03E-58 | GO:0002253 | activation of immune response |
| 9.58E-60 | 1.47E-58 | GO:0009894 | regulation of catabolic process |
| 1.39E-58 | 2.05E-57 | GO:0019221 | cytokine-mediated signaling pathway |
| 1.82E-57 | 2.57E-56 | GO:0044265 | cellular macromolecule catabolic process |
| 8.92E-57 | 1.21E-55 | GO:1903706 | regulation of hemopoiesis |
| 1.01E-56 | 8.93E-56 | GO:0010564 | regulation of cell cycle process |
| 1.17E-56 | 1.52E-55 | GO:0062012 | regulation of small molecule metabolic process |
| 1.88E-56 | 1.62E-55 | GO:0031347 | regulation of defense response |
| 7.43E-56 | 9.37E-55 | GO:0051726 | regulation of cell cycle |
| 2.54E-55 | 2.11E-54 | GO:0050778 | positive regulation of immune response |
| 4.47E-55 | 3.61E-54 | GO:0006401 | RNA catabolic process |
| 4.67E-55 | 6.16E-54 | GO:0033554 | cellular response to stress |
| 2.08E-54 | 2.53E-53 | GO:0002009 | morphogenesis of an epithelium |
| 1.99E-53 | 2.34E-52 | GO:0080134 | regulation of response to stress |
| 5.74E-53 | 6.54E-52 | GO:0034097 | response to cytokine |
| 1.81E-52 | 2.25E-51 | GO:0045087 | innate immune response |
| 2.46E-52 | 2.72E-51 | GO:0006508 | proteolysis |
| 3.02E-52 | 3.23E-51 | GO:0002684 | positive regulation of immune system process |
| 3.56E-52 | 1.10E-50 | GO:0009628 | response to abiotic stimulus |
| 4.71E-52 | 3.70E-51 | GO:0071345 | cellular response to cytokine stimulus |
| 2.98E-51 | 3.50E-50 | GO:1901575 | organic substance catabolic process |
| 4.45E-51 | 1.03E-49 | GO:0022402 | cell cycle process |
| 2.94E-50 | 3.26E-49 | GO:0048729 | tissue morphogenesis |
| 4.02E-49 | 3.07E-48 | GO:0031329 | regulation of cellular catabolic process |
| 8.12E-49 | 8.44E-48 | GO:0050776 | regulation of immune response |
| 9.67E-49 | 9.76E-48 | GO:0000165 | MAPK cascade |
| 5.52E-48 | 5.82E-47 | GO:0023014 | signal transduction by protein phosphorylation |
| 1.71E-46 | 3.18E-45 | GO:0007049 | cell cycle |
| 1.89E-46 | 1.85E-45 | GO:0034655 | nucleobase-containing compound catabolic process |
| 6.78E-45 | 1.05E-43 | GO:0009056 | catabolic process |
| 7.09E-45 | 6.76E-44 | GO:0046700 | heterocycle catabolic process |
| 8.22E-45 | 7.64E-44 | GO:0044270 | cellular nitrogen compound catabolic process |
| 2.40E-44 | 2.17E-43 | GO:0048534 | hematopoietic or lymphoid organ development |
| 2.64E-44 | 2.33E-43 | GO:0019439 | aromatic compound catabolic process |
| 3.01E-44 | 3.02E-43 | GO:0009887 | animal organ morphogenesis |
| 3.67E-44 | 3.52E-43 | GO:0002682 | regulation of immune system process |
| 5.36E-44 | 3.98E-43 | GO:0030097 | hemopoiesis |
| 1.28E-43 | 1.10E-42 | GO:0048585 | negative regulation of response to stimulus |
| 2.34E-43 | 1.96E-42 | GO:1901361 | organic cyclic compound catabolic process |
| 4.10E-43 | 5.45E-42 | GO:0002520 | immune system development |
| 5.11E-43 | 3.70E-42 | GO:0009968 | negative regulation of signal transduction |
| 1.16E-41 | 1.06E-40 | GO:0010033 | response to organic substance |
| 2.33E-41 | 1.91E-40 | GO:0060429 | epithelium development |
| 4.60E-41 | 3.24E-40 | GO:0010648 | negative regulation of cell communication |
| 5.44E-41 | 4.36E-40 | GO:0023057 | negative regulation of signaling |
| 1.02E-39 | 8.95E-39 | GO:0007166 | cell surface receptor signaling pathway |
| 1.45E-39 | 1.22E-38 | GO:0006952 | defense response |
| 1.54E-39 | 1.79E-38 | GO:0007267 | cell-cell signaling |
| 4.92E-39 | 3.86E-38 | GO:0071310 | cellular response to organic substance |
| 5.04E-39 | 3.46E-38 | GO:0019752 | carboxylic acid metabolic process |
| 2.95E-38 | 2.26E-37 | GO:0022603 | regulation of anatomical structure morphogenesis |
| 5.02E-38 | 4.07E-37 | GO:0035556 | intracellular signal transduction |
| 1.39E-37 | 1.04E-36 | GO:0036211 | protein modification process |
| 1.39E-37 | 9.30E-37 | GO:0006464 | cellular protein modification process |
| 2.02E-37 | 1.58E-36 | GO:0044248 | cellular catabolic process |
| 3.01E-37 | 2.27E-36 | GO:0070887 | cellular response to chemical stimulus |
| 3.79E-37 | 2.79E-36 | GO:0043436 | oxoacid metabolic process |
| 7.96E-37 | 5.79E-36 | GO:0006082 | organic acid metabolic process |
| 8.57E-37 | 6.17E-36 | GO:0048584 | positive regulation of response to stimulus |
| 1.44E-36 | 9.46E-36 | GO:0010605 | negative regulation of macromolecule metabolic process |
| 1.99E-36 | 1.40E-35 | GO:0055085 | transmembrane transport |
| 4.74E-36 | 3.28E-35 | GO:0043412 | macromolecule modification |
| 1.00E-35 | 1.04E-34 | GO:0006950 | response to stress |
| 2.96E-35 | 1.90E-34 | GO:0016310 | phosphorylation |
| 3.70E-35 | 2.60E-34 | GO:0019538 | protein metabolic process |
| 4.31E-35 | 2.69E-34 | GO:0009967 | positive regulation of signal transduction |
| 2.62E-34 | 2.44E-33 | GO:0006955 | immune response |
| 3.65E-34 | 2.48E-33 | GO:0009892 | negative regulation of metabolic process |
| 6.78E-34 | 4.51E-33 | GO:0045595 | regulation of cell differentiation |
| 3.74E-33 | 2.55E-32 | GO:0048519 | negative regulation of biological process |
| 5.82E-33 | 3.55E-32 | GO:0010647 | positive regulation of cell communication |
| 7.00E-33 | 4.57E-32 | GO:0023056 | positive regulation of signaling |
| 2.21E-32 | 1.46E-31 | GO:0048518 | positive regulation of biological process |
| 2.75E-32 | 1.76E-31 | GO:0044267 | cellular protein metabolic process |
| 5.71E-32 | 4.83E-31 | GO:0042221 | response to chemical |
| 9.90E-32 | 6.33E-31 | GO:0048583 | regulation of response to stimulus |
| 1.32E-31 | 8.21E-31 | GO:1901564 | organonitrogen compound metabolic process |
| 3.75E-31 | 2.26E-30 | GO:0009888 | tissue development |
| 7.11E-31 | 4.48E-30 | GO:0048523 | negative regulation of cellular process |
| 1.25E-30 | 7.71E-30 | GO:2000026 | regulation of multicellular organismal development |
| 1.92E-29 | 1.17E-28 | GO:0009966 | regulation of signal transduction |
| 8.51E-29 | 6.59E-28 | GO:0007165 | signal transduction |
| 1.71E-27 | 1.02E-26 | GO:0080090 | regulation of primary metabolic process |
| 2.73E-27 | 1.60E-26 | GO:0051171 | regulation of nitrogen compound metabolic process |
| 5.25E-27 | 3.76E-26 | GO:0007154 | cell communication |
| 5.44E-27 | 3.15E-26 | GO:0010646 | regulation of cell communication |
| 6.00E-27 | 3.59E-26 | GO:0042176 | regulation of protein catabolic process |
| 7.60E-27 | 4.33E-26 | GO:0048522 | positive regulation of cellular process |
| 7.84E-27 | 4.39E-26 | GO:0006796 | phosphate-containing compound metabolic process |
| 8.45E-27 | 4.95E-26 | GO:0050793 | regulation of developmental process |
| 1.36E-26 | 7.74E-26 | GO:0023051 | regulation of signaling |
| 2.81E-26 | 1.87E-25 | GO:0051716 | cellular response to stimulus |
| 3.64E-26 | 2.01E-25 | GO:0031323 | regulation of cellular metabolic process |
| 4.32E-26 | 2.40E-25 | GO:0006793 | phosphorus metabolic process |
| 8.65E-26 | 5.37E-25 | GO:0009653 | anatomical structure morphogenesis |
| 9.64E-26 | 5.60E-25 | GO:0044281 | small molecule metabolic process |
| 1.12E-25 | 6.06E-25 | GO:0048513 | animal organ development |
| 2.86E-25 | 1.55E-24 | GO:0060255 | regulation of macromolecule metabolic process |
| 3.77E-25 | 1.99E-24 | GO:0050794 | regulation of cellular process |
| 5.73E-25 | 3.13E-24 | GO:0065008 | regulation of biological quality |
| 9.42E-25 | 4.85E-24 | GO:0019222 | regulation of metabolic process |
| 9.58E-25 | 5.60E-24 | GO:0006351 | transcription, DNA-templated |
| 1.59E-24 | 9.10E-24 | GO:0032774 | RNA biosynthetic process |
| 2.59E-24 | 1.30E-23 | GO:0051239 | regulation of multicellular organismal process |
| 3.19E-24 | 1.79E-23 | GO:0051252 | regulation of RNA metabolic process |
| 1.52E-23 | 8.10E-23 | GO:0044271 | cellular nitrogen compound biosynthetic process |
| 5.48E-23 | 3.01E-22 | GO:0019219 | regulation of nucleobase-containing compound metabolic process |
| 9.07E-23 | 4.68E-22 | GO:0009058 | biosynthetic process |
| 1.07E-22 | 5.26E-22 | GO:0050789 | regulation of biological process |
| 1.09E-22 | 5.73E-22 | GO:0009889 | regulation of biosynthetic process |
| 1.57E-22 | 8.16E-22 | GO:0009059 | macromolecule biosynthetic process |
| 2.32E-22 | 1.25E-21 | GO:0010556 | regulation of macromolecule biosynthetic process |
| 2.59E-22 | 1.33E-21 | GO:0034645 | cellular macromolecule biosynthetic process |
| 3.31E-22 | 1.75E-21 | GO:0031326 | regulation of cellular biosynthetic process |
| 3.59E-22 | 1.76E-21 | GO:1901576 | organic substance biosynthetic process |
| 3.72E-22 | 1.78E-21 | GO:0043170 | macromolecule metabolic process |
| 5.50E-22 | 2.78E-21 | GO:0034654 | nucleobase-containing compound biosynthetic process |
| 1.11E-21 | 5.20E-21 | GO:0044249 | cellular biosynthetic process |
| 1.28E-21 | 6.38E-21 | GO:0018130 | heterocycle biosynthetic process |
| 1.32E-21 | 6.45E-21 | GO:1901362 | organic cyclic compound biosynthetic process |
| 1.42E-21 | 6.89E-21 | GO:0019438 | aromatic compound biosynthetic process |
| 4.79E-21 | 2.19E-20 | GO:0030154 | cell differentiation |
| 8.14E-21 | 3.88E-20 | GO:0090304 | nucleic acid metabolic process |
| 9.66E-21 | 4.49E-20 | GO:0048869 | cellular developmental process |
| 2.64E-20 | 1.17E-19 | GO:0006807 | nitrogen compound metabolic process |
| 4.65E-20 | 2.41E-19 | GO:0010468 | regulation of gene expression |
| 1.91E-19 | 8.58E-19 | GO:0034641 | cellular nitrogen compound metabolic process |
| 2.56E-19 | 1.13E-18 | GO:0044260 | cellular macromolecule metabolic process |
| 2.68E-19 | 1.36E-18 | GO:0016070 | RNA metabolic process |
| 1.00E-18 | 4.33E-18 | GO:0048731 | system development |
| 2.11E-18 | 8.93E-18 | GO:0044238 | primary metabolic process |
| 3.60E-18 | 1.52E-17 | GO:0006810 | transport |
| 9.20E-18 | 3.81E-17 | GO:0006139 | nucleobase-containing compound metabolic process |
| 1.18E-17 | 4.79E-17 | GO:0051234 | establishment of localization |
| 2.40E-17 | 9.31E-17 | GO:0007275 | multicellular organism development |
| 3.34E-17 | 1.67E-16 | GO:0000082 | G1/S transition of mitotic cell cycle |
| 3.38E-17 | 1.26E-16 | GO:0044237 | cellular metabolic process |
| 3.53E-17 | 1.26E-16 | GO:0071704 | organic substance metabolic process |
| 5.15E-17 | 2.09E-16 | GO:0046483 | heterocycle metabolic process |
| 7.78E-17 | 3.10E-16 | GO:0006725 | cellular aromatic compound metabolic process |
| 1.07E-16 | 5.04E-16 | GO:0044843 | cell cycle G1/S phase transition |
| 1.11E-16 | 5.44E-16 | GO:0051246 | regulation of protein metabolic process |
| 1.12E-16 | 4.36E-16 | GO:1901360 | organic cyclic compound metabolic process |
| 1.24E-16 | 4.27E-16 | GO:0048856 | anatomical structure development |
| 9.86E-16 | 4.58E-15 | GO:0010467 | gene expression |
| 2.20E-14 | 1.01E-13 | GO:0016032 | viral process |
| 1.25E-13 | 4.78E-13 | GO:0044403 | symbiont process |
| 3.99E-13 | 1.33E-12 | GO:0044419 | interspecies interaction between organisms |
| 2.20E-12 | 1.06E-11 | GO:0032268 | regulation of cellular protein metabolic process |
| 6.58E-12 | 3.12E-11 | GO:0006517 | protein deglycosylation |
| 1.29E-10 | 6.00E-10 | GO:0051173 | positive regulation of nitrogen compound metabolic process |
| 1.68E-10 | 7.61E-10 | GO:0034976 | response to endoplasmic reticulum stress |
| 4.94E-10 | 2.27E-09 | GO:0010604 | positive regulation of macromolecule metabolic process |
| 5.51E-10 | 2.46E-09 | GO:0043312 | neutrophil degranulation |
| 8.74E-10 | 3.94E-09 | GO:0002446 | neutrophil mediated immunity |
| 9.28E-10 | 4.10E-09 | GO:0036230 | granulocyte activation |
| 2.28E-09 | 8.60E-09 | GO:0043299 | leukocyte degranulation |
| 2.77E-09 | 1.21E-08 | GO:0002275 | myeloid cell activation involved in immune response |
| 3.13E-09 | 1.35E-08 | GO:0071824 | protein-DNA complex subunit organization |
| 3.27E-09 | 1.38E-08 | GO:0002444 | myeloid leukocyte mediated immunity |
| 3.29E-09 | 1.38E-08 | GO:0044819 | mitotic G1/S transition checkpoint |
| 3.59E-09 | 1.49E-08 | GO:0002274 | myeloid leukocyte activation |
| 3.69E-09 | 1.64E-08 | GO:0044783 | G1 DNA damage checkpoint |
| 4.35E-09 | 1.78E-08 | GO:0009893 | positive regulation of metabolic process |
| 5.87E-09 | 2.38E-08 | GO:1903513 | endoplasmic reticulum to cytosol transport |
| 6.34E-09 | 2.73E-08 | GO:0072401 | signal transduction involved in DNA integrity checkpoint |
| 6.34E-09 | 2.73E-08 | GO:0072422 | signal transduction involved in DNA damage checkpoint |
| 7.04E-09 | 2.82E-08 | GO:0072395 | signal transduction involved in cell cycle checkpoint |
| 9.43E-09 | 3.49E-08 | GO:0044093 | positive regulation of molecular function |
| 9.75E-09 | 3.87E-08 | GO:0000077 | DNA damage checkpoint |
| 1.03E-08 | 4.35E-08 | GO:1902047 | polyamine transmembrane transport |
| 1.48E-08 | 5.80E-08 | GO:0002366 | leukocyte activation involved in immune response |
| 1.60E-08 | 6.66E-08 | GO:0009896 | positive regulation of catabolic process |
| 1.62E-08 | 5.88E-08 | GO:0002263 | cell activation involved in immune response |
| 1.76E-08 | 6.28E-08 | GO:0031570 | DNA integrity checkpoint |
| 2.03E-08 | 8.32E-08 | GO:0031325 | positive regulation of cellular metabolic process |
| 2.31E-08 | 8.13E-08 | GO:0050790 | regulation of catalytic activity |
| 2.39E-08 | 9.66E-08 | GO:0015846 | polyamine transport |
| 2.63E-08 | 1.05E-07 | GO:1904029 | regulation of cyclin-dependent protein kinase activity |
| 3.10E-08 | 1.20E-07 | GO:0042770 | signal transduction in response to DNA damage |
| 3.59E-08 | 1.38E-07 | GO:0031098 | stress-activated protein kinase signaling cascade |
| 4.08E-08 | 1.55E-07 | GO:0072413 | signal transduction involved in mitotic cell cycle checkpoint |
| 4.85E-08 | 1.82E-07 | GO:0035872 | nucleotide-binding domain, leucine rich repeat containing receptor signaling pathway |
| 5.80E-08 | 2.15E-07 | GO:0051091 | positive regulation of DNA binding transcription factor activity |
| 5.83E-08 | 2.29E-07 | GO:0051092 | positive regulation of NF-kappaB transcription factor activity |
| 6.34E-08 | 2.33E-07 | GO:0010243 | response to organonitrogen compound |
| 6.36E-08 | 2.46E-07 | GO:0043067 | regulation of programmed cell death |
| 6.95E-08 | 2.53E-07 | GO:0010941 | regulation of cell death |
| 8.03E-08 | 3.07E-07 | GO:0044773 | mitotic DNA damage checkpoint |
| 8.76E-08 | 2.81E-07 | GO:0045321 | leukocyte activation |
| 9.44E-08 | 3.56E-07 | GO:0065004 | protein-DNA complex assembly |
| 9.78E-08 | 3.03E-07 | GO:0065009 | regulation of molecular function |
| 1.06E-07 | 3.83E-07 | GO:0007249 | I-kappaB kinase/NF-kappaB signaling |
| 1.07E-07 | 3.71E-07 | GO:1901698 | response to nitrogen compound |
| 1.07E-07 | 3.99E-07 | GO:0030330 | DNA damage response, signal transduction by p53 class mediator |
| 1.23E-07 | 4.40E-07 | GO:0044774 | mitotic DNA integrity checkpoint |
| 1.36E-07 | 4.91E-07 | GO:0060548 | negative regulation of cell death |
| 1.36E-07 | 4.91E-07 | GO:0009309 | amine biosynthetic process |
| 1.60E-07 | 4.81E-07 | GO:0001775 | cell activation |
| 2.20E-07 | 7.50E-07 | GO:0051090 | regulation of DNA binding transcription factor activity |
| 2.40E-07 | 8.56E-07 | GO:0032479 | regulation of type I interferon production |
| 2.55E-07 | 8.55E-07 | GO:0032606 | type I interferon production |
| 2.63E-07 | 7.65E-07 | GO:0000075 | cell cycle checkpoint |
| 3.65E-07 | 1.29E-06 | GO:0045055 | regulated exocytosis |
| 6.95E-07 | 2.29E-06 | GO:0007050 | cell cycle arrest |
| 7.53E-07 | 2.63E-06 | GO:0002753 | cytoplasmic pattern recognition receptor signaling pathway |
| 1.03E-06 | 3.63E-06 | GO:0051403 | stress-activated MAPK cascade |
| 1.09E-06 | 3.78E-06 | GO:0034622 | cellular protein-containing complex assembly |
| 1.80E-06 | 6.19E-06 | GO:0051347 | positive regulation of transferase activity |
| 2.17E-06 | 7.03E-06 | GO:0002443 | leukocyte mediated immunity |
| 2.38E-06 | 7.60E-06 | GO:0007093 | mitotic cell cycle checkpoint |
| 2.61E-06 | 8.22E-06 | GO:0006887 | exocytosis |
| 2.85E-06 | 9.88E-06 | GO:0043085 | positive regulation of catalytic activity |
| 3.13E-06 | 1.06E-05 | GO:0045787 | positive regulation of cell cycle |
| 4.35E-06 | 1.49E-05 | GO:0007219 | Notch signaling pathway |
| 4.39E-06 | 1.49E-05 | GO:0006915 | apoptotic process |
| 4.54E-06 | 1.41E-05 | GO:0012501 | programmed cell death |
| 4.64E-06 | 1.29E-05 | GO:0008219 | cell death |
| 4.71E-06 | 1.29E-05 | GO:0002252 | immune effector process |
| 5.67E-06 | 1.90E-05 | GO:0000715 | nucleotide-excision repair, DNA damage recognition |
| 5.78E-06 | 1.91E-05 | GO:0002221 | pattern recognition receptor signaling pathway |
| 5.81E-06 | 1.95E-05 | GO:0006974 | cellular response to DNA damage stimulus |
| 6.78E-06 | 2.22E-05 | GO:0006297 | nucleotide-excision repair, DNA gap filling |
| 7.52E-06 | 2.50E-05 | GO:0065003 | protein-containing complex assembly |
| 8.02E-06 | 2.60E-05 | GO:0010942 | positive regulation of cell death |
| 8.92E-06 | 2.85E-05 | GO:0006576 | cellular biogenic amine metabolic process |
| 1.23E-05 | 4.05E-05 | GO:0051338 | regulation of transferase activity |
| 1.29E-05 | 4.06E-05 | GO:0010939 | regulation of necrotic cell death |
| 1.30E-05 | 4.08E-05 | GO:1903827 | regulation of cellular protein localization |
| 2.35E-05 | 7.27E-05 | GO:0051172 | negative regulation of nitrogen compound metabolic process |
| 3.29E-05 | 0.000100752 | GO:0043933 | protein-containing complex subunit organization |
| 4.00E-05 | 0.000122185 | GO:0070266 | necroptotic process |
| 4.20E-05 | 0.000126808 | GO:0032386 | regulation of intracellular transport |
| 4.45E-05 | 0.000134179 | GO:0042769 | DNA damage response, detection of DNA damage |
| 4.94E-05 | 0.000147676 | GO:0019068 | virion assembly |
| 5.07E-05 | 0.000149952 | GO:0000271 | polysaccharide biosynthetic process |
| 5.29E-05 | 0.000154815 | GO:0009891 | positive regulation of biosynthetic process |
| 5.45E-05 | 0.000162041 | GO:0031647 | regulation of protein stability |
| 5.54E-05 | 0.000162433 | GO:0097190 | apoptotic signaling pathway |
| 6.60E-05 | 0.000191004 | GO:0031334 | positive regulation of protein complex assembly |
| 6.64E-05 | 0.000217133 | GO:0097300 | programmed necrotic cell death |
| 7.99E-05 | 0.000228966 | GO:1903573 | negative regulation of response to endoplasmic reticulum stress |
| 8.80E-05 | 0.000284978 | GO:0072331 | signal transduction by p53 class mediator |
| 0.000106342 | 0.000341262 | GO:0051051 | negative regulation of transport |
| 0.000118683 | 0.000377433 | GO:0006112 | energy reserve metabolic process |
| 0.00012478 | 0.000393279 | GO:0035994 | response to muscle stretch |
| 0.00013139 | 0.000372498 | GO:0008593 | regulation of Notch signaling pathway |
| 0.000136081 | 0.00038186 | GO:0071897 | DNA biosynthetic process |
| 0.000142762 | 0.000445973 | GO:0006886 | intracellular protein transport |
| 0.000143094 | 0.000413599 | GO:0070265 | necrotic cell death |
| 0.00015194 | 0.000470481 | GO:0016192 | vesicle-mediated transport |
| 0.000159831 | 0.000443974 | GO:0090261 | positive regulation of inclusion body assembly |
| 0.000175855 | 0.000467271 | GO:0032940 | secretion by cell |
| 0.000181679 | 0.000557676 | GO:0046903 | secretion |
| 0.000195501 | 0.000537629 | GO:0031324 | negative regulation of cellular metabolic process |
| 0.000196072 | 0.000596667 | GO:0070841 | inclusion body assembly |
| 0.000214554 | 0.000584181 | GO:0009890 | negative regulation of biosynthetic process |
| 0.000232388 | 0.000701136 | GO:0006259 | DNA metabolic process |
| 0.000235453 | 0.000662407 | GO:0071214 | cellular response to abiotic stimulus |
| 0.000235453 | 0.000662407 | GO:0104004 | cellular response to environmental stimulus |
| 0.000265479 | 0.000715753 | GO:0009446 | putrescine biosynthetic process |
| 0.000287158 | 0.00085904 | GO:0046794 | transport of virus |
| 0.000296924 | 0.000880792 | GO:0060341 | regulation of cellular localization |
| 0.000304398 | 0.000895437 | GO:0009100 | glycoprotein metabolic process |
| 0.000323748 | 0.000944488 | GO:0005976 | polysaccharide metabolic process |

**Supplementary Table 6.** Gene coexpression modules.

| **Module** | **Gene** | **Module** | **Gene** | **Module** | **Gene** | **Module** | **Gene** |
| --- | --- | --- | --- | --- | --- | --- | --- |
| blue | CACNG6 | blue | RELA | brown | CDK4 | red | KCNIP2 |
| blue | FXYD3 | blue | FGFR2 | brown | PSMB2 | red | TMOD3 |
| blue | MYBPC1 | blue | NFKBIA | brown | P4HB | red | PSME4 |
| blue | ATP1A4 | blue | PDGFRB | brown | CDC34 | red | MAP3K7 |
| blue | FXYD2 | blue | TPM2 | brown | EZR | red | DERL2 |
| blue | IL2RG | blue | CCND1 | brown | MCM7 | red | KIT |
| blue | CD209 | blue | PHLPP1 | brown | TYMS | red | CDC7 |
| blue | PSMB9 | blue | ATP1B2 | brown | PCNA | red | FXYD7 |
| blue | CLEC7A | blue | KCNIP1 | brown | ODC1 | red | PIP5K1A |
| blue | FCGR1B | blue | RANGRF | brown | VIM | red | UBE2D1 |
| blue | RYR1 | blue | AHCYL1 | green | PSMD9 | red | MAX |
| blue | CSF2RB | blue | DAG1 | green | PSMC6 | red | PPP2R5E |
| blue | LMOD1 | blue | OAZ2 | green | RPA3 | red | MAPK3 |
| blue | IL1B | blue | SRI | green | CDK7 | red | TRAPPC4 |
| blue | FCGR1A | blue | PSME1 | green | ORC5 | red | PSMD11 |
| blue | NFATC2 | blue | ATP1A2 | green | DMPK | red | CSNK2A2 |
| blue | PIK3R5 | blue | MYL12A | green | SORBS1 | red | AKT3 |
| blue | PLN | blue | FGFR3 | green | PSMD5 | red | PIK3R3 |
| blue | CD86 | blue | UBE2M | green | PSMD6 | red | NRAS |
| blue | NFATC1 | blue | SDC3 | green | CCNH | red | CUL1 |
| blue | MYL3 | blue | TPM4 | green | ORC4 | red | CSNK2A1 |
| blue | ITGA10 | blue | RHOB | green | ORC3 | red | PAK2 |
| blue | PTPN6 | blue | MYL6 | green | PTEN | red | PSMD2 |
| blue | ITGB4 | blue | RHOA | green | UBA3 | red | CDKN1B |
| blue | DHH | brown | MYH3 | green | KRAS | turquoise | CACNA1F |
| blue | GPC5 | brown | FGF7 | green | PSMD14 | turquoise | NEB |
| blue | LAMA3 | brown | TNNC1 | green | PSMD7 | turquoise | CARD11 |
| blue | RAC2 | brown | MYLPF | green | PSMA3 | turquoise | FGF5 |
| blue | KCNJ14 | brown | EGF | green | PSMA6 | turquoise | BTC |
| blue | DES | brown | ITGA2B | green | PSMC2 | turquoise | IL2RB |
| blue | CLIC2 | brown | SCN5A | green | PSMA5 | turquoise | KCNK13 |
| blue | ACTG2 | brown | LAMA1 | green | PSMA2 | turquoise | SCN1B |
| blue | NOTUM | brown | HHAT | green | FGF13 | turquoise | SCN7A |
| blue | PSMB8 | brown | ATM | green | UBE2V1 | turquoise | PIK3CG |
| blue | MYH11 | brown | OAZ3 | green | PSMD13 | turquoise | CNTNAP1 |
| blue | NPR1 | brown | POLE2 | green | PSME3 | turquoise | RYR2 |
| blue | KCNQ1 | brown | ITGA2 | green | PSMA1 | turquoise | CACNA1D |
| blue | SCUBE2 | brown | NRG1 | green | PSMD4 | turquoise | CACNG5 |
| blue | SYK | brown | CHEK2 | green | FKBP1B | turquoise | SCN1A |
| blue | DDR2 | brown | EGFR | green | PSMA4 | turquoise | CD19 |
| blue | ITPR2 | brown | MNAT1 | green | PSMD10 | turquoise | KCNJ12 |
| blue | ITPR3 | brown | NFKB1 | green | PSMD1 | turquoise | ITPR1 |
| blue | MYL4 | brown | PIK3CD | green | ATP1B3 | turquoise | KCNIP3 |
| blue | LAMA5 | brown | CARD9 | green | UBE2N | turquoise | SCN4B |
| blue | PIK3R6 | brown | DBF4 | green | RPA2 | turquoise | PRKACG |
| blue | IKBKB | brown | ORC2 | green | HRAS | turquoise | ANK1 |
| blue | KCNE4 | brown | MCM8 | green | SKP1 | turquoise | NPPC |
| blue | KCNE3 | brown | GAB1 | green | MYL6B | turquoise | ATP2A3 |
| blue | LAMA2 | brown | NRP1 | green | UBE2D2 | turquoise | PLCG2 |
| blue | PYCARD | brown | ORC1 | green | PSMB1 | turquoise | PRKCD |
| blue | FCER1G | brown | KIF4B | green | PSMC3 | turquoise | CNTN6 |
| blue | FXYD1 | brown | CCNE2 | green | CLTA | turquoise | LAMB3 |
| blue | NFKB2 | brown | MCM10 | green | AZIN1 | turquoise | CACNG1 |
| blue | ABCC9 | brown | CHEK1 | green | AP2S1 | turquoise | ITGA9 |
| blue | HSPG2 | brown | PRIM2 | green | RPS27A | turquoise | CACNA1C |
| blue | LYN | brown | POLA2 | green | PSMC4 | turquoise | SCN2B |
| blue | LAMC3 | brown | NFATC3 | green | CDC42 | turquoise | CACNG2 |
| blue | ITGB5 | brown | WEE1 | green | PSMB5 | turquoise | VAV3 |
| blue | FGF2 | brown | CKS1B | green | PSMB4 | turquoise | KCNIP4 |
| blue | CDKN1A | brown | MRC2 | green | PSMD8 | turquoise | SCN8A |
| blue | KAT2B | brown | CDC6 | green | MYL12B | turquoise | CACNB4 |
| blue | KCNK9 | brown | KCNK10 | green | PSMA7 | turquoise | KCNK12 |
| blue | ITGA1 | brown | CDC45 | green | PSMB3 | turquoise | KCNK1 |
| blue | ICAM3 | brown | ADAM17 | green | PSMB6 | turquoise | SPTB |
| blue | PSME2 | brown | ERBB2 | green | PSMB7 | turquoise | CACNB2 |
| blue | PPP2R5A | brown | NRP2 | green | UBA52 | turquoise | PHLPP2 |
| blue | ANXA2 | brown | CDC25A | green | OAZ1 | turquoise | PIP5K1B |
| blue | NQO1 | brown | FBXO5 | green | HSPA8 | turquoise | CACNG3 |
| blue | ANXA1 | brown | ORC6 | green | UBB | turquoise | CCNE1 |
| blue | ORAI1 | brown | KIF4A | red | SPTBN5 | turquoise | TRPC1 |
| blue | ACTA2 | brown | DHFR | red | PTK6 | turquoise | SLC8A3 |
| blue | ITGA5 | brown | RB1 | red | GP6 | turquoise | SPTBN4 |
| blue | SDC4 | brown | POLA1 | red | CACNA1S | turquoise | ATP2B3 |
| blue | WWTR1 | brown | PRIM1 | red | KCND1 | turquoise | KCNJ11 |
| blue | ASPH | brown | CDK2 | red | FGF22 | turquoise | KCNK3 |
| blue | HBEGF | brown | SKP2 | red | KCNK7 | turquoise | NRG3 |
| blue | LAMA4 | brown | GMNN | red | NRG4 | turquoise | NUMB |
| blue | PXN | brown | CDT1 | red | ATP2A1 | turquoise | NFASC |
| blue | SHC1 | brown | TNC | red | JAK3 | turquoise | ACTN1 |
| blue | LAMB2 | brown | TAB2 | red | LAT | turquoise | RPS6KA3 |
| blue | PDGFRA | brown | MCM5 | red | VTN | turquoise | CACNG8 |
| blue | PDGFB | brown | E2F1 | red | NRG2 | turquoise | KCNQ3 |
| blue | MYL9 | brown | PDPN | red | KCNK4 | turquoise | NPPA |
| blue | ITGAV | brown | RRM2 | red | KLB | turquoise | KCNJ4 |
| blue | ICAM2 | brown | ITGB1 | red | KCNK6 | turquoise | FGF12 |
| blue | SORBS3 | brown | MAP2K2 | red | THEM4 | turquoise | STIM1 |
| blue | SDC2 | brown | CDK1 | red | RELB | turquoise | DLG1 |
| blue | LAMB1 | brown | TFDP1 | red | PDGFA | turquoise | ATP2B2 |
| blue | TGFB1 | brown | MCM3 | red | RPS6KA4 | turquoise | KCND3 |
| blue | MYL5 | brown | CDC25B | red | MALT1 | turquoise | PDPK1 |
| blue | FGFR1 | brown | RDX | red | CHUK | turquoise | PRKCA |
| blue | ITGA6 | brown | CCNA2 | red | TK2 | turquoise | CACNA2D1 |
| blue | TLN1 | brown | CCNB1 | red | RPS6KA5 | turquoise | SCN2A |
| blue | VCL | brown | MCM4 | red | TRAF6 | turquoise | AKT2 |
| blue | CALD1 | brown | MCM6 | red | JAK2 | turquoise | CACNA2D2 |
| blue | FGF11 | brown | MSN | red | RPS6KA6 | turquoise | ERBB4 |
| blue | LAMC1 | brown | RPA1 | red | BCL10 | turquoise | PIK3CB |
| blue | DNM2 | brown | MCM2 | red | FRS2 | turquoise | PIP4K2C |
| blue | PSMB10 | brown | DDR1 | red | PIK3CA | turquoise | KCND2 |
| yellow | GAB2 | turquoise | ANK3 | turquoise | NRCAM | yellow | LCP2 |
| yellow | CREBBP | turquoise | DNM3 | turquoise | ERLEC1 | yellow | FGF18 |
| yellow | CNTN2 | turquoise | IRS2 | turquoise | TPM3 | yellow | THBS1 |
| yellow | EP300 | turquoise | ALCAM | turquoise | ANK2 | yellow | MYLK |
| yellow | INSR | turquoise | ATP2B1 | turquoise | PPP2R5B | yellow | DYSF |
| yellow | TAB1 | turquoise | CAMK2G | turquoise | SPTBN2 | yellow | KCNE2 |
| yellow | VAV2 | turquoise | PSMD12 | turquoise | TMOD2 | yellow | PSMC1 |
| yellow | SCN3A | turquoise | CAMK2B | turquoise | PTPN11 | yellow | SCN9A |
| yellow | CASK | turquoise | SLC8A2 | turquoise | ATP2A2 | yellow | IKBKG |
| yellow | SRC | turquoise | TPM1 | turquoise | FBXW11 | yellow | POLE |
| yellow | AGRN | turquoise | PPP2R5C | turquoise | MAPK1 | yellow | TAB3 |
| yellow | CHL1 | turquoise | SLC8A1 | turquoise | PRKACA | yellow | TNNT1 |
| yellow | CACNB3 | turquoise | CAMK2A | turquoise | KCNQ2 | yellow | DISP2 |
| yellow | EPHB2 | turquoise | SEL1L | turquoise | MAP2K1 | yellow | AKAP9 |
| yellow | RAF1 | turquoise | PIK3R1 | turquoise | PAK1 | yellow | TRIB3 |
| yellow | CACNG7 | turquoise | ANXA6 | turquoise | SPTBN1 | yellow | NPR2 |
| yellow | FZR1 | turquoise | PAK3 | turquoise | PPP3R1 | yellow | RHOG |
| yellow | PSMF1 | turquoise | CACNB1 | turquoise | ATP1B1 | yellow | KCNH2 |
| yellow | AKT1 | turquoise | HIPK1 | turquoise | PRKACB | yellow | MYC |
| yellow | CSNK2B | turquoise | PKMYT1 | turquoise | FYN | yellow | KCNK2 |
| yellow | PPP2CB | turquoise | CAMK2D | turquoise | PPP3CA | yellow | SDC1 |
| yellow | OS9 | turquoise | CACNA2D3 | turquoise | PPP3CB | yellow | IRS1 |
| yellow | PSMD3 | turquoise | PIP5K1C | turquoise | PPP2R5D | yellow | PIP4K2A |
| yellow | L1CAM | turquoise | JAK1 | turquoise | AP2A1 | yellow | TMOD1 |
| yellow | GRB2 | turquoise | AP2A2 | turquoise | ATP1A1 | yellow | HIPK2 |
| yellow | PIK3R2 | turquoise | PRKCZ | turquoise | DLG4 |  |  |
| yellow | SPTAN1 | turquoise | ATP2B4 | turquoise | PPP2CA |  |  |
| yellow | VCP | turquoise | DNM1 | turquoise | NCAN |  |  |
| yellow | NCAM1 | turquoise | RPS6KA2 | turquoise | PSMC5 |  |  |
| yellow | PIP4K2B | turquoise | SH3GL2 | turquoise | AP2M1 |  |  |
| yellow | FXYD6 | turquoise | DLG3 | turquoise | CALM1 |  |  |
| yellow | CACNG4 | turquoise | NRXN1 | turquoise | ATP1A3 |  |  |
| yellow | AP2B1 | turquoise | BTRC | yellow | FGF1 |  |  |
| yellow | PPP2R1A | turquoise | CNTN1 | yellow | KCNE1 |  |  |
| yellow | DPYSL2 | turquoise | RANBP9 | yellow | CLEC4A |  |  |
| yellow | DCX | turquoise | FGF14 | yellow | ERBB3 |  |  |
| yellow | UBC | turquoise | SCN3B | yellow | ITGB3 |  |  |

**Supplementary Table 7.** Based on the NetRep program, the Yellow, Blue, Red and Turquoise modules were replicated in 15 brain regions.

| > preservation$hip$p.value | |  |  |  |  |  |
| --- | --- | --- | --- | --- | --- | --- |
| **avg.weight coherence cor.cor cor.degree cor.contrib avg.cor avg.contrib** | | | | | | |
| 1 0.00349965 9.999e-05 9.999e-05 9.999e-05 9.999e-05 9.999e-05 9.999e-05 | | | | | | |
| 2 0.00009999 9.999e-05 9.999e-05 9.999e-05 9.999e-05 9.999e-05 9.999e-05 | | | | | | |
| 3 0.00009999 9.999e-05 9.999e-05 9.999e-05 9.999e-05 9.999e-05 9.999e-05 | | | | | | |
| 4 0.00009999 9.999e-05 9.999e-05 9.999e-05 9.999e-05 9.999e-05 9.999e-05 | | | | | | |
| 5 0.00009999 9.999e-05 9.999e-05 9.999e-05 9.999e-05 9.999e-05 9.999e-05 | | | | | | |
| 6 0.00239976 9.999e-05 9.999e-05 9.999e-05 9.999e-05 9.999e-05 9.999e-05 | | | | | | |
| > preservation$amy$p.value | |  |  |  |  |  |
| **avg.weight coherence cor.cor cor.degree cor.contrib avg.cor avg.contrib** | | | | | | |
| 1 0.00149985 9.999e-05 9.999e-05 9.999e-05 9.999e-05 9.999e-05 9.999e-05 | | | | | | |
| 2 0.00009999 9.999e-05 9.999e-05 9.999e-05 9.999e-05 9.999e-05 9.999e-05 | | | | | | |
| 3 0.04489551 9.999e-05 9.999e-05 9.999e-05 9.999e-05 9.999e-05 9.999e-05 | | | | | | |
| 4 0.00009999 9.999e-05 9.999e-05 9.999e-05 9.999e-05 9.999e-05 9.999e-05 | | | | | | |
| 5 0.00009999 9.999e-05 9.999e-05 9.999e-05 9.999e-05 9.999e-05 9.999e-05 | | | | | | |
| 6 0.00209979 9.999e-05 9.999e-05 9.999e-05 9.999e-05 9.999e-05 9.999e-05 | | | | | | |
| > preservation$a1c$p.value | |  |  |  |  |  |
| **avg.weight coherence cor.cor cor.degree cor.contrib avg.cor avg.contrib** | | | | | | |
| 1 0.00009999 9.999e-05 9.999e-05 9.999e-05 9.999e-05 9.999e-05 9.999e-05 | | | | | | |
| 2 0.00009999 9.999e-05 9.999e-05 9.999e-05 9.999e-05 9.999e-05 9.999e-05 | | | | | | |
| 3 0.00019998 9.999e-05 9.999e-05 9.999e-05 9.999e-05 9.999e-05 9.999e-05 | | | | | | |
| 4 0.00009999 9.999e-05 9.999e-05 9.999e-05 9.999e-05 9.999e-05 9.999e-05 | | | | | | |
| 5 0.00009999 9.999e-05 9.999e-05 9.999e-05 9.999e-05 9.999e-05 9.999e-05 | | | | | | |
| 6 0.00009999 9.999e-05 9.999e-05 9.999e-05 9.999e-05 9.999e-05 9.999e-05 | | | | | | |
| > preservation$dfc$p.value | |  |  |  |  |  |
| **avg.weight coherence cor.cor cor.degree cor.contrib avg.cor avg.contrib** | | | | | | |
| 1 0.00049995 9.999e-05 9.999e-05 9.999e-05 9.999e-05 9.999e-05 9.999e-05 | | | | | | |
| 2 0.00009999 9.999e-05 9.999e-05 9.999e-05 9.999e-05 9.999e-05 9.999e-05 | | | | | | |
| 3 0.00009999 9.999e-05 9.999e-05 9.999e-05 9.999e-05 9.999e-05 9.999e-05 | | | | | | |
| 4 0.00009999 9.999e-05 9.999e-05 9.999e-05 9.999e-05 9.999e-05 9.999e-05 | | | | | | |
| 5 0.00009999 9.999e-05 9.999e-05 9.999e-05 9.999e-05 9.999e-05 9.999e-05 | | | | | | |
| 6 0.00019998 9.999e-05 9.999e-05 9.999e-05 9.999e-05 9.999e-05 9.999e-05 | | | | | | |
| > preservation$ipc$p.value | |  |  |  |  |  |
| **avg.weight coherence cor.cor cor.degree cor.contrib avg.cor avg.contrib** | | | | | | |
| 1 0.00009999 0.00009999 9.999e-05 9.999e-05 9.999e-05 9.999e-05 0.00009999 | | | | | | |
| 2 0.96460354 0.64833517 9.999e-05 9.999e-05 9.999e-05 9.999e-05 0.02299770 | | | | | | |
| 3 0.00009999 0.00009999 9.999e-05 9.999e-05 9.999e-05 9.999e-05 0.00009999 | | | | | | |
| 4 0.00009999 0.00009999 9.999e-05 9.999e-05 9.999e-05 9.999e-05 0.00009999 | | | | | | |
| 5 0.00009999 0.00009999 9.999e-05 9.999e-05 9.999e-05 9.999e-05 0.00009999 | | | | | | |
| 6 0.00009999 0.00009999 9.999e-05 9.999e-05 9.999e-05 9.999e-05 0.00009999 | | | | | | |
| > preservation$itc$p.value | |  |  |  |  |  |
| **avg.weight coherence cor.cor cor.degree cor.contrib avg.cor avg.contrib** | | | | | | |
| 1 9.999e-05 9.999e-05 9.999e-05 9.999e-05 9.999e-05 9.999e-05 9.999e-05 | | | | | | |
| 2 9.999e-05 9.999e-05 9.999e-05 9.999e-05 9.999e-05 9.999e-05 9.999e-05 | | | | | | |
| 3 9.999e-05 9.999e-05 9.999e-05 9.999e-05 9.999e-05 9.999e-05 9.999e-05 | | | | | | |
| 4 9.999e-05 9.999e-05 9.999e-05 9.999e-05 9.999e-05 9.999e-05 9.999e-05 | | | | | | |
| 5 9.999e-05 9.999e-05 9.999e-05 9.999e-05 9.999e-05 9.999e-05 9.999e-05 | | | | | | |
| 6 9.999e-05 9.999e-05 9.999e-05 9.999e-05 9.999e-05 9.999e-05 9.999e-05 | | | | | | |
| > preservation$m1c$p.value | |  |  |  |  |  |
| **avg.weight coherence cor.cor cor.degree cor.contrib avg.cor avg.contrib** | | | | | | |
| 1 0.00009999 0.00009999 9.999e-05 0.00009999 9.999e-05 9.999e-05 0.00009999 | | | | | | |
| 2 0.52534747 0.47115288 9.999e-05 0.10618938 9.999e-05 9.999e-05 0.00089991 | | | | | | |
| 3 0.00009999 0.00009999 9.999e-05 0.00009999 9.999e-05 9.999e-05 0.00009999 | | | | | | |
| 4 0.00009999 0.00009999 9.999e-05 0.00009999 9.999e-05 9.999e-05 0.00009999 | | | | | | |
| 5 0.00009999 0.00009999 9.999e-05 0.00009999 9.999e-05 9.999e-05 0.00009999 | | | | | | |
| 6 0.00009999 0.00009999 9.999e-05 0.00009999 9.999e-05 9.999e-05 0.00009999 | | | | | | |
| > preservation$md$p.value |  |  |  |  |  |  |
| **avg.weight coherence cor.cor cor.degree cor.contrib avg.cor avg.contrib** | | | | | | |
| 1 0.00009999 0.00009999 9.999e-05 9.999e-05 9.999e-05 9.999e-05 9.999e-05 | | | | | | |
| 2 0.00789921 0.00249975 9.999e-05 9.999e-05 9.999e-05 9.999e-05 9.999e-05 | | | | | | |
| 3 0.00009999 0.00009999 9.999e-05 9.999e-05 9.999e-05 9.999e-05 9.999e-05 | | | | | | |
| 4 0.02029797 0.00009999 9.999e-05 9.999e-05 9.999e-05 9.999e-05 9.999e-05 | | | | | | |
| 5 0.00009999 0.00009999 9.999e-05 9.999e-05 9.999e-05 9.999e-05 9.999e-05 | | | | | | |
| 6 0.00009999 0.00009999 9.999e-05 9.999e-05 9.999e-05 9.999e-05 9.999e-05 | | | | | | |
| > preservation$mfc$p.value | |  |  |  |  |  |
| **avg.weight coherence cor.cor cor.degree cor.contrib avg.cor avg.contrib** | | | | | | |
| 1 0.00689931 9.999e-05 9.999e-05 9.999e-05 9.999e-05 9.999e-05 9.999e-05 | | | | | | |
| 2 0.00009999 9.999e-05 9.999e-05 9.999e-05 9.999e-05 9.999e-05 9.999e-05 | | | | | | |
| 3 0.00009999 9.999e-05 9.999e-05 9.999e-05 9.999e-05 9.999e-05 9.999e-05 | | | | | | |
| 4 0.00119988 9.999e-05 9.999e-05 9.999e-05 9.999e-05 9.999e-05 9.999e-05 | | | | | | |
| 5 0.00009999 9.999e-05 9.999e-05 9.999e-05 9.999e-05 9.999e-05 9.999e-05 | | | | | | |
| 6 0.00039996 9.999e-05 9.999e-05 9.999e-05 9.999e-05 9.999e-05 9.999e-05 | | | | | | |
| > preservation$ofc$p.value | |  |  |  |  |  |
| **avg.weight coherence cor.cor cor.degree cor.contrib avg.cor avg.contrib** | | | | | | |
| 1 0.00319968 9.999e-05 9.999e-05 9.999e-05 9.999e-05 9.999e-05 9.999e-05 | | | | | | |
| 2 0.00009999 9.999e-05 9.999e-05 9.999e-05 9.999e-05 9.999e-05 9.999e-05 | | | | | | |
| 3 0.00009999 9.999e-05 9.999e-05 9.999e-05 9.999e-05 9.999e-05 9.999e-05 | | | | | | |
| 4 0.00009999 9.999e-05 9.999e-05 9.999e-05 9.999e-05 9.999e-05 9.999e-05 | | | | | | |
| 5 0.00009999 9.999e-05 9.999e-05 9.999e-05 9.999e-05 9.999e-05 9.999e-05 | | | | | | |
| 6 0.00169983 9.999e-05 9.999e-05 9.999e-05 9.999e-05 9.999e-05 9.999e-05 | | | | | | |
| > preservation$s1c$p.value | |  |  |  |  |  |
| **avg.weight coherence cor.cor cor.degree cor.contrib avg.cor avg.contrib** | | | | | | |
| 1 0.00009999 0.00009999 9.999e-05 0.00009999 9.999e-05 9.999e-05 0.00009999 | | | | | | |
| 2 0.91850815 0.64113589 9.999e-05 0.01559844 9.999e-05 9.999e-05 0.09839016 | | | | | | |
| 3 0.00009999 0.00009999 9.999e-05 0.00009999 9.999e-05 9.999e-05 0.00009999 | | | | | | |
| 4 0.00009999 0.00009999 9.999e-05 0.00009999 9.999e-05 9.999e-05 0.00009999 | | | | | | |
| 5 0.00009999 0.00009999 9.999e-05 0.00009999 9.999e-05 9.999e-05 0.00009999 | | | | | | |
| 6 0.00009999 0.00009999 9.999e-05 0.00009999 9.999e-05 9.999e-05 0.00009999 | | | | | | |
| > preservation$stc$p.value | |  |  |  |  |  |
| **avg.weight coherence cor.cor cor.degree cor.contrib avg.cor avg.contrib** | | | | | | |
| 1 0.00009999 9.999e-05 9.999e-05 9.999e-05 9.999e-05 9.999e-05 9.999e-05 | | | | | | |
| 2 0.00009999 9.999e-05 9.999e-05 9.999e-05 9.999e-05 9.999e-05 9.999e-05 | | | | | | |
| 3 0.00009999 9.999e-05 9.999e-05 9.999e-05 9.999e-05 9.999e-05 9.999e-05 | | | | | | |
| 4 0.00019998 9.999e-05 9.999e-05 9.999e-05 9.999e-05 9.999e-05 9.999e-05 | | | | | | |
| 5 0.00009999 9.999e-05 9.999e-05 9.999e-05 9.999e-05 9.999e-05 9.999e-05 | | | | | | |
| 6 0.00009999 9.999e-05 9.999e-05 9.999e-05 9.999e-05 9.999e-05 9.999e-05 | | | | | | |
| > preservation$str$p.value | |  |  |  |  |  |
| **avg.weight coherence cor.cor cor.degree cor.contrib avg.cor avg.contrib** | | | | | | |
| 1 0.00009999 0.00009999 9.999e-05 9.999e-05 9.999e-05 9.999e-05 9.999e-05 | | | | | | |
| 2 0.00009999 0.00009999 9.999e-05 9.999e-05 9.999e-05 9.999e-05 9.999e-05 | | | | | | |
| 3 0.00009999 0.00009999 9.999e-05 9.999e-05 9.999e-05 9.999e-05 9.999e-05 | | | | | | |
| 4 0.00009999 0.00009999 9.999e-05 9.999e-05 9.999e-05 9.999e-05 9.999e-05 | | | | | | |
| 5 0.00089991 0.09529047 9.999e-05 9.999e-05 9.999e-05 9.999e-05 9.999e-05 | | | | | | |
| 6 0.00009999 0.00009999 9.999e-05 9.999e-05 9.999e-05 9.999e-05 9.999e-05 | | | | | | |
| > preservation$v1c$p.value | |  |  |  |  |  |
| **avg.weight coherence cor.cor cor.degree cor.contrib avg.cor avg.contrib** | | | | | | |
| 1 0.00009999 9.999e-05 9.999e-05 9.999e-05 9.999e-05 9.999e-05 9.999e-05 | | | | | | |
| 2 0.00009999 9.999e-05 9.999e-05 9.999e-05 9.999e-05 9.999e-05 9.999e-05 | | | | | | |
| 3 0.02829717 9.999e-05 9.999e-05 9.999e-05 9.999e-05 9.999e-05 9.999e-05 | | | | | | |
| 4 0.00009999 9.999e-05 9.999e-05 9.999e-05 9.999e-05 9.999e-05 9.999e-05 | | | | | | |
| 5 0.00009999 9.999e-05 9.999e-05 9.999e-05 9.999e-05 9.999e-05 9.999e-05 | | | | | | |
| 6 0.00009999 9.999e-05 9.999e-05 9.999e-05 9.999e-05 9.999e-05 9.999e-05 | | | | | | |
| > preservation$vfc$p.value | |  |  |  |  |  |
| **avg.weight coherence cor.cor cor.degree cor.contrib avg.cor avg.contrib** | | | | | | |
| 1 0.00009999 9.999e-05 9.999e-05 9.999e-05 9.999e-05 9.999e-05 9.999e-05 | | | | | | |
| 2 0.00009999 9.999e-05 9.999e-05 9.999e-05 9.999e-05 9.999e-05 9.999e-05 | | | | | | |
| 3 0.00199980 9.999e-05 9.999e-05 9.999e-05 9.999e-05 9.999e-05 9.999e-05 | | | | | | |
| 4 0.00009999 9.999e-05 9.999e-05 9.999e-05 9.999e-05 9.999e-05 9.999e-05 | | | | | | |
| 5 0.00009999 9.999e-05 9.999e-05 9.999e-05 9.999e-05 9.999e-05 9.999e-05 | | | | | | |
| 6 0.00009999 9.999e-05 9.999e-05 9.999e-05 9.999e-05 9.999e-05 9.999e-05 | | | | | | |

**Supplementary Table 8.** Functional enrichment of these genes in the red module.

| **p-value** | **q-value** | **term_goid** | **term_name** |
| --- | --- | --- | --- |
| 7.46E-22 | 1.75E-19 | GO:0016310 | phosphorylation |
| 3.78E-19 | 4.44E-17 | GO:0002220 | innate immune response activating cell surface receptor signaling pathway |
| 1.09E-18 | 2.12E-16 | GO:0006793 | phosphorus metabolic process |
| 4.62E-18 | 1.50E-15 | GO:0006796 | phosphate-containing compound metabolic process |
| 6.46E-18 | 6.30E-16 | GO:0035556 | intracellular signal transduction |
| 1.19E-15 | 1.69E-13 | GO:0002768 | immune response-regulating cell surface receptor signaling pathway |
| 1.56E-15 | 1.69E-13 | GO:0000165 | MAPK cascade |
| 2.95E-15 | 1.92E-13 | GO:0023014 | signal transduction by protein phosphorylation |
| 5.04E-15 | 2.46E-13 | GO:0007166 | cell surface receptor signaling pathway |
| 7.12E-15 | 5.78E-13 | GO:0002429 | immune response-activating cell surface receptor signaling pathway |
| 1.69E-14 | 1.10E-12 | GO:0048584 | positive regulation of response to stimulus |
| 4.00E-14 | 2.17E-12 | GO:0002758 | innate immune response-activating signal transduction |
| 9.62E-14 | 3.59E-12 | GO:0002218 | activation of innate immune response |
| 1.07E-13 | 8.41E-12 | GO:0031349 | positive regulation of defense response |
| 1.11E-13 | 3.59E-12 | GO:0002764 | immune response-regulating signaling pathway |
| 2.02E-13 | 1.19E-11 | GO:0070498 | interleukin-1-mediated signaling pathway |
| 2.58E-13 | 1.20E-11 | GO:0036211 | protein modification process |
| 2.58E-13 | 1.21E-11 | GO:0006464 | cellular protein modification process |
| 3.30E-13 | 1.87E-11 | GO:0002253 | activation of immune response |
| 4.62E-13 | 1.87E-11 | GO:0051716 | cellular response to stimulus |
| 5.48E-13 | 2.23E-11 | GO:0050776 | regulation of immune response |
| 5.60E-13 | 2.04E-11 | GO:0045088 | regulation of innate immune response |
| 6.68E-13 | 1.86E-11 | GO:0002757 | immune response-activating signal transduction |
| 6.92E-13 | 2.04E-11 | GO:0009967 | positive regulation of signal transduction |
| 6.96E-13 | 2.04E-11 | GO:0046834 | lipid phosphorylation |
| 1.06E-12 | 2.85E-11 | GO:0007165 | signal transduction |
| 1.13E-12 | 3.88E-11 | GO:0043412 | macromolecule modification |
| 1.19E-12 | 3.88E-11 | GO:0007167 | enzyme linked receptor protein signaling pathway |
| 2.20E-12 | 5.37E-11 | GO:0048583 | regulation of response to stimulus |
| 2.81E-12 | 7.35E-11 | GO:0050851 | antigen receptor-mediated signaling pathway |
| 3.18E-12 | 6.43E-11 | GO:0007154 | cell communication |
| 3.46E-12 | 8.13E-11 | GO:0007169 | transmembrane receptor protein tyrosine kinase signaling pathway |
| 5.08E-12 | 1.09E-10 | GO:0010647 | positive regulation of cell communication |
| 5.48E-12 | 1.62E-10 | GO:0023056 | positive regulation of signaling |
| 7.62E-12 | 1.24E-10 | GO:0065009 | regulation of molecular function |
| 8.23E-12 | 1.61E-10 | GO:0050778 | positive regulation of immune response |
| 1.23E-11 | 2.67E-10 | GO:0010033 | response to organic substance |
| 2.95E-11 | 5.76E-10 | GO:0051239 | regulation of multicellular organismal process |
| 3.16E-11 | 8.55E-10 | GO:0043491 | protein kinase B signaling |
| 4.27E-11 | 5.77E-10 | GO:0042221 | response to chemical |
| 4.51E-11 | 8.16E-10 | GO:0031347 | regulation of defense response |
| 6.07E-11 | 1.08E-09 | GO:0006952 | defense response |
| 6.97E-11 | 1.74E-09 | GO:0038061 | NIK/NF-kappaB signaling |
| 9.77E-11 | 2.27E-09 | GO:0002684 | positive regulation of immune system process |
| 1.26E-10 | 2.04E-09 | GO:0045087 | innate immune response |
| 1.49E-10 | 2.51E-09 | GO:0070555 | response to interleukin-1 |
| 1.99E-10 | 4.32E-09 | GO:0071310 | cellular response to organic substance |
| 2.35E-10 | 3.68E-09 | GO:1902531 | regulation of intracellular signal transduction |
| 2.99E-10 | 4.40E-09 | GO:0006650 | glycerophospholipid metabolic process |
| 3.34E-10 | 6.78E-09 | GO:0044267 | cellular protein metabolic process |
| 7.20E-10 | 8.33E-09 | GO:0006950 | response to stress |
| 7.41E-10 | 1.11E-08 | GO:0002682 | regulation of immune system process |
| 7.83E-10 | 1.50E-08 | GO:0048522 | positive regulation of cellular process |
| 9.40E-10 | 9.52E-09 | GO:0050789 | regulation of biological process |
| 1.02E-09 | 1.42E-08 | GO:0023051 | regulation of signaling |
| 1.18E-09 | 2.12E-08 | GO:0080134 | regulation of response to stress |
| 1.23E-09 | 1.59E-08 | GO:0048518 | positive regulation of biological process |
| 2.09E-09 | 2.55E-08 | GO:0070887 | cellular response to chemical stimulus |
| 2.39E-09 | 4.08E-08 | GO:0009966 | regulation of signal transduction |
| 3.32E-09 | 5.39E-08 | GO:0019221 | cytokine-mediated signaling pathway |
| 3.48E-09 | 5.39E-08 | GO:0046486 | glycerolipid metabolic process |
| 3.86E-09 | 4.43E-08 | GO:0002367 | cytokine production involved in immune response |
| 4.48E-09 | 6.19E-08 | GO:0051174 | regulation of phosphorus metabolic process |
| 5.27E-09 | 7.78E-08 | GO:0006644 | phospholipid metabolic process |
| 5.54E-09 | 7.83E-08 | GO:0030258 | lipid modification |
| 8.68E-09 | 9.40E-08 | GO:0048731 | system development |
| 1.40E-08 | 1.44E-07 | GO:0019538 | protein metabolic process |
| 2.00E-08 | 2.71E-07 | GO:0010646 | regulation of cell communication |
| 2.40E-08 | 3.12E-07 | GO:0051091 | positive regulation of DNA binding transcription factor activity |
| 2.48E-08 | 3.24E-07 | GO:0046474 | glycerophospholipid biosynthetic process |
| 2.69E-08 | 3.33E-07 | GO:0002718 | regulation of cytokine production involved in immune response |
| 2.99E-08 | 3.51E-07 | GO:0032268 | regulation of cellular protein metabolic process |
| 3.54E-08 | 3.96E-07 | GO:0051246 | regulation of protein metabolic process |
| 4.74E-08 | 4.26E-07 | GO:0006955 | immune response |
| 6.29E-08 | 7.87E-07 | GO:0045017 | glycerolipid biosynthetic process |
| 7.11E-08 | 8.56E-07 | GO:0034097 | response to cytokine |
| 9.65E-08 | 1.12E-06 | GO:0008654 | phospholipid biosynthetic process |
| 1.06E-07 | 1.03E-06 | GO:0048519 | negative regulation of biological process |
| 1.12E-07 | 9.10E-07 | GO:0048856 | anatomical structure development |
| 1.13E-07 | 1.05E-06 | GO:0044093 | positive regulation of molecular function |
| 1.44E-07 | 1.54E-06 | GO:0051092 | positive regulation of NF-kappaB transcription factor activity |
| 1.67E-07 | 1.87E-06 | GO:0048523 | negative regulation of cellular process |
| 1.79E-07 | 1.83E-06 | GO:0071345 | cellular response to cytokine stimulus |
| 1.90E-07 | 1.68E-06 | GO:0051090 | regulation of DNA binding transcription factor activity |
| 1.99E-07 | 1.47E-06 | GO:0007275 | multicellular organism development |
| 2.50E-07 | 2.45E-06 | GO:0051347 | positive regulation of transferase activity |
| 3.27E-07 | 3.07E-06 | GO:0051173 | positive regulation of nitrogen compound metabolic process |
| 3.76E-07 | 3.19E-06 | GO:1901564 | organonitrogen compound metabolic process |
| 3.80E-07 | 4.11E-06 | GO:0051338 | regulation of transferase activity |
| 4.39E-07 | 2.96E-06 | GO:0001816 | cytokine production |
| 6.53E-07 | 5.30E-06 | GO:0033554 | cellular response to stress |
| 6.88E-07 | 6.22E-06 | GO:0060047 | heart contraction |
| 7.24E-07 | 6.30E-06 | GO:0010604 | positive regulation of macromolecule metabolic process |
| 7.50E-07 | 7.86E-06 | GO:0003015 | heart process |
| 7.50E-07 | 5.85E-06 | GO:0032879 | regulation of localization |
| 8.24E-07 | 6.92E-06 | GO:0002700 | regulation of production of molecular mediator of immune response |
| 9.04E-07 | 5.63E-06 | GO:0001775 | cell activation |
| 1.10E-06 | 6.35E-06 | GO:0006928 | movement of cell or subcellular component |
| 1.30E-06 | 1.32E-05 | GO:0001817 | regulation of cytokine production |
| 1.35E-06 | 1.01E-05 | GO:0044255 | cellular lipid metabolic process |
| 1.44E-06 | 1.04E-05 | GO:0006810 | transport |
| 1.72E-06 | 1.20E-05 | GO:0050790 | regulation of catalytic activity |
| 2.02E-06 | 1.09E-05 | GO:0065008 | regulation of biological quality |
| 2.34E-06 | 1.18E-05 | GO:0051234 | establishment of localization |
| 2.63E-06 | 2.59E-05 | GO:0009893 | positive regulation of metabolic process |
| 2.68E-06 | 2.17E-05 | GO:0031325 | positive regulation of cellular metabolic process |
| 2.71E-06 | 1.82E-05 | GO:0061337 | cardiac conduction |
| 2.88E-06 | 2.73E-05 | GO:0048732 | gland development |
| 2.94E-06 | 2.73E-05 | GO:0006915 | apoptotic process |
| 4.03E-06 | 3.15E-05 | GO:0008284 | positive regulation of cell proliferation |
| 4.18E-06 | 3.78E-05 | GO:0048872 | homeostasis of number of cells |
| 4.60E-06 | 2.99E-05 | GO:0048513 | animal organ development |
| 4.69E-06 | 3.56E-05 | GO:0048015 | phosphatidylinositol-mediated signaling |
| 5.06E-06 | 4.43E-05 | GO:0042110 | T cell activation |
| 5.24E-06 | 4.43E-05 | GO:0048017 | inositol lipid-mediated signaling |
| 5.31E-06 | 4.43E-05 | GO:0043085 | positive regulation of catalytic activity |
| 5.40E-06 | 2.57E-05 | GO:0002252 | immune effector process |
| 6.27E-06 | 3.94E-05 | GO:0044260 | cellular macromolecule metabolic process |
| 7.28E-06 | 3.28E-05 | GO:0048869 | cellular developmental process |
| 7.39E-06 | 4.50E-05 | GO:0012501 | programmed cell death |
| 8.07E-06 | 6.55E-05 | GO:0007399 | nervous system development |
| 9.50E-06 | 5.61E-05 | GO:0050794 | regulation of cellular process |
| 9.85E-06 | 4.20E-05 | GO:0002440 | production of molecular mediator of immune response |
| 1.15E-05 | 8.86E-05 | GO:0030322 | stabilization of membrane potential |
| 1.15E-05 | 8.86E-05 | GO:0045785 | positive regulation of cell adhesion |
| 1.28E-05 | 9.38E-05 | GO:1903522 | regulation of blood circulation |
| 1.43E-05 | 0.000107868 | GO:0080090 | regulation of primary metabolic process |
| 1.52E-05 | 5.87E-05 | GO:0048870 | cell motility |
| 1.52E-05 | 5.87E-05 | GO:0051674 | localization of cell |
| 1.73E-05 | 0.000128106 | GO:0031323 | regulation of cellular metabolic process |
| 1.74E-05 | 6.40E-05 | GO:0008219 | cell death |
| 1.85E-05 | 6.50E-05 | GO:0035637 | multicellular organismal signaling |
| 2.03E-05 | 0.000116217 | GO:0019637 | organophosphate metabolic process |
| 2.06E-05 | 0.000149051 | GO:0042127 | regulation of cell proliferation |
| 2.19E-05 | 7.39E-05 | GO:0009719 | response to endogenous stimulus |
| 2.19E-05 | 0.00015499 | GO:0090407 | organophosphate biosynthetic process |
| 2.62E-05 | 0.000181192 | GO:0051171 | regulation of nitrogen compound metabolic process |
| 2.66E-05 | 0.000189494 | GO:0010564 | regulation of cell cycle process |
| 2.71E-05 | 0.000183821 | GO:0048534 | hematopoietic or lymphoid organ development |
| 2.97E-05 | 0.000165631 | GO:0002250 | adaptive immune response |
| 3.06E-05 | 0.000165831 | GO:0006629 | lipid metabolic process |
| 3.32E-05 | 0.000229642 | GO:0046686 | response to cadmium ion |
| 3.59E-05 | 0.000237797 | GO:0050865 | regulation of cell activation |
| 3.61E-05 | 0.000190116 | GO:0030154 | cell differentiation |
| 3.62E-05 | 0.00011716 | GO:0019882 | antigen processing and presentation |
| 3.80E-05 | 0.000118467 | GO:0009653 | anatomical structure morphogenesis |
| 3.93E-05 | 0.000201651 | GO:0006935 | chemotaxis |
| 3.98E-05 | 0.000119431 | GO:0042330 | taxis |
| 4.46E-05 | 0.000129035 | GO:0002520 | immune system development |
| 4.60E-05 | 0.000298807 | GO:0006936 | muscle contraction |
| 4.68E-05 | 0.000234136 | GO:0007596 | blood coagulation |
| 4.87E-05 | 0.000310443 | GO:0006954 | inflammatory response |
| 5.04E-05 | 0.000140737 | GO:0050817 | coagulation |
| 5.11E-05 | 0.000314644 | GO:0060255 | regulation of macromolecule metabolic process |
| 5.13E-05 | 0.000314644 | GO:0007599 | hemostasis |
| 6.19E-05 | 0.000167043 | GO:0016043 | cellular component organization |
| 6.47E-05 | 0.000315224 | GO:0071495 | cellular response to endogenous stimulus |
| 6.70E-05 | 0.000402941 | GO:0007411 | axon guidance |
| 6.81E-05 | 0.000457472 | GO:0042590 | antigen processing and presentation of exogenous peptide antigen via MHC class I |
| 7.00E-05 | 0.000332877 | GO:0097485 | neuron projection guidance |
| 7.31E-05 | 0.000477431 | GO:0022409 | positive regulation of cell-cell adhesion |
| 7.64E-05 | 0.000440116 | GO:0051726 | regulation of cell cycle |
| 7.69E-05 | 0.000440116 | GO:0048585 | negative regulation of response to stimulus |
| 7.85E-05 | 0.000440116 | GO:0044057 | regulation of system process |
| 7.85E-05 | 0.000440116 | GO:0008015 | blood circulation |
| 8.30E-05 | 0.000376812 | GO:0003013 | circulatory system process |
| 8.31E-05 | 0.000376812 | GO:0030168 | platelet activation |
| 8.51E-05 | 0.00046856 | GO:0007249 | I-kappaB kinase/NF-kappaB signaling |
| 8.91E-05 | 0.00048275 | GO:0022407 | regulation of cell-cell adhesion |
| 9.16E-05 | 0.000405758 | GO:0019222 | regulation of metabolic process |
| 9.24E-05 | 0.000587181 | GO:0034762 | regulation of transmembrane transport |
| 9.51E-05 | 0.000506619 | GO:0051049 | regulation of transport |
| 9.97E-05 | 0.00061646 | GO:0030097 | hemopoiesis |
| 0.000103112 | 0.000621314 | GO:0050867 | positive regulation of cell activation |
| 0.000104096 | 0.000545667 | GO:0016477 | cell migration |
| 0.000114122 | 0.000588724 | GO:0044772 | mitotic cell cycle phase transition |
| 0.000117071 | 0.000676759 | GO:0007259 | JAK-STAT cascade |
| 0.000118073 | 0.000599589 | GO:0002369 | T cell cytokine production |
| 0.000118073 | 0.000676759 | GO:0097421 | liver regeneration |
| 0.000123279 | 0.000608345 | GO:0006508 | proteolysis |
| 0.000123639 | 0.000608345 | GO:0097696 | STAT cascade |
| 0.000123639 | 0.00069179 | GO:0033209 | tumor necrosis factor-mediated signaling pathway |
| 0.000127027 | 0.000608345 | GO:0002478 | antigen processing and presentation of exogenous peptide antigen |
| 0.000127285 | 0.000608345 | GO:0008610 | lipid biosynthetic process |
| 0.000128167 | 0.000555389 | GO:0002443 | leukocyte mediated immunity |
| 0.000137621 | 0.00073461 | GO:0002699 | positive regulation of immune effector process |
| 0.000140324 | 0.00073461 | GO:0060416 | response to growth hormone |
| 0.00014067 | 0.00073461 | GO:0001819 | positive regulation of cytokine production |
| 0.000144003 | 0.000376267 | GO:0009605 | response to external stimulus |
| 0.000146789 | 0.000686891 | GO:0002474 | antigen processing and presentation of peptide antigen via MHC class I |
| 0.000147946 | 0.000686891 | GO:0048666 | neuron development |
| 0.000151169 | 0.000382646 | GO:0045321 | leukocyte activation |
| 0.000151615 | 0.000694012 | GO:0002697 | regulation of immune effector process |
| 0.000152784 | 0.000647671 | GO:0019884 | antigen processing and presentation of exogenous antigen |
| 0.000155884 | 0.000702683 | GO:0046649 | lymphocyte activation |
| 0.000157833 | 0.000702683 | GO:0031098 | stress-activated protein kinase signaling cascade |
| 0.000158508 | 0.000657638 | GO:0003012 | muscle system process |
| 0.000161362 | 0.000708683 | GO:0002371 | dendritic cell cytokine production |
| 0.000161362 | 0.000819168 | GO:0032762 | mast cell cytokine production |
| 0.000163834 | 0.000819168 | GO:1903037 | regulation of leukocyte cell-cell adhesion |
| 0.0001735 | 0.000849429 | GO:0002221 | pattern recognition receptor signaling pathway |
| 0.000178543 | 0.000773686 | GO:0035872 | nucleotide-binding domain, leucine rich repeat containing receptor signaling pathway |
| 0.000178543 | 0.000856277 | GO:0042088 | T-helper 1 type immune response |
| 0.000182362 | 0.000727914 | GO:0048002 | antigen processing and presentation of peptide antigen |
| 0.000182912 | 0.000727914 | GO:0044770 | cell cycle phase transition |
| 0.000183345 | 0.00078404 | GO:0043434 | response to peptide hormone |
| 0.000199068 | 0.000840224 | GO:0009894 | regulation of catabolic process |
| 0.000206099 | 0.000776588 | GO:0030030 | cell projection organization |
| 0.000206462 | 0.000776588 | GO:0043170 | macromolecule metabolic process |
| 0.00020709 | 0.000776588 | GO:0009888 | tissue development |
| 0.000207382 | 0.000974696 | GO:0034612 | response to tumor necrosis factor |
